# Time- and spatially resolved LNA delivery via thermally controlled SPION technology

**DOI:** 10.1016/j.omtn.2026.102902

**Published:** 2026-03-13

**Authors:** Franziska Kenneweg, Katharina Hempel, Lukas Philipp Joachim Höhne, Gerald Dräger, Jonas Blume, Thilo Viereck, Anastasia Stohwasser, Sonja Groß, Gwen Büchler, Karina Jansen, Malte Juchem, Christian Bär, Angelika Pfanne, Annette Just, Sabrina Thum, Anika Gietz, Andreas Kirschning, Thomas Thum

**Affiliations:** 1 Institute of Molecular and Translational Therapeutic Strategies (IMTTS), Hannover Medical School, 30625 Hannover, Germany; 2 Institute of Organic Chemistry, Leibniz University Hannover, 30167 Hannover, Germany; 3 Institute for Electrical Measurement Science and Fundamental Electrical Engineering (emg), TU Braunschweig, 38106 Braunschweig, Germany; 4 Laboratory for Emerging Nanometrology (LENA), TU Braunschweig, 38106 Braunschweig, Germany; 5 Uppsala Biomedical Center (BMC), Uppsala University, 75237 Uppsala, Sweden

**Keywords:** MT: Delivery Strategies, nanoparticles, drug conjugates, linker, hyperthermia, microRNA, miR-21

## Abstract

Targeted RNA delivery with precise spatial and temporal control marks a significant advancement in therapeutic development, offering the potential to reduce drug dosages while minimizing off-target effects. In this study, we present a novel platform that employs superparamagnetic iron oxide nanoparticles (SPIONs) for externally controlled, thermally triggered, organ-specific locked nucleic acid (LNA) release. Our platform technology leverages a newly designed thermosensitive conjugate, based on a thermosensitive linker system that utilizes the thermal sensitivity of the *tert*-butyloxycarbonyl (Boc) group. This tool ensures stability during systemic circulation while enabling traceless, on-demand drug release at the target site. As a proof of concept, we applied this technology in a disease model of cardiac fibrosis, conjugating SPIONs with an inhibitor of microRNA (miRNA)-21, a key pro-fibrotic regulator. The nanoparticle system was thoroughly characterized for its stability, biocompatibility, and heat-induced release properties *in vitro* and subsequently validated for biodistribution, toxicology, and therapeutic potential in preclinical *in vivo* models. This innovative SPION-based delivery platform provides a versatile and precise framework for RNA-based therapeutics, with broad translational potential across various disease applications.

## Graphical abstract

**Figure:**
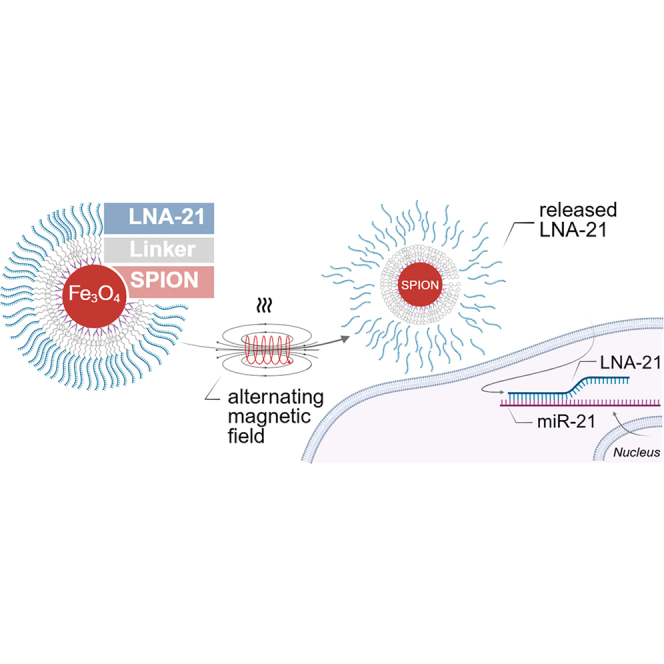

## Teaser

Thum and colleagues report an intriguing, novel tool: an externally controllable SPION-based drug delivery system that enables targeted RNA inhibition with minimized off-target effects and high on-target efficiency. The concept can be readily adapted to other drugs and can target a range of organs and disease sites.

## Introduction

The development of RNA-based therapeutics has opened new frontiers in precision medicine,^1^^,^^2^ yet their clinical translation continues to be limited by challenges in achieving targeted, efficient, and controllable delivery.^3^ Issues such as off-target effects, low cellular uptake, and potential immune responses limit the effectiveness of currently, usually systemically applied RNA delivery platforms.^4^^,^^5^^,^^6^ To address these challenges, we introduce an advanced transport system leveraging superparamagnetic iron oxide nanoparticles (SPIONs)^7^^,^^8^ in combination with a thermosensitive linker system, which is itself bound to a drug,^9^^,^^10^^,^^11^^,^^12^ enabling externally controlled, time- and space-resolved RNA delivery.

SPIONs exhibit distinct magnetic properties that enable precise manipulation, real-time tracking, and remote heat induction through externally applied magnetic fields.^13^^,^^14^^,^^15^^,^^16^ By integrating a thermosensitive conjugate based on the heat sensitivity of the *tert*-butyloxycarbonyl group (Boc),^17^^,^^18^^,^^19^ we have developed a delivery system that remains stable during systemic circulation but enables traceless, thermally triggered release of its therapeutic cargo at the target site. This technology provides an innovative solution for RNA therapeutics by overcoming critical delivery barriers and ensuring localized, controlled drug activation.

As a proof of concept, we conjugated an inhibitor of microRNA (miRNA)-21, demonstrating its effective use in modulating fibrotic pathways.^20^^,^^21^^,^^22^ *In vitro* studies confirmed the system’s stability, biocompatibility, and tunable release properties, followed by validation in preclinical models. However, this approach is not limited to cardiac applications—it represents a broadly adaptable delivery technology with potential across various therapeutic areas. By enabling precise spatial and temporal control over RNA-based therapeutics, SPION-based delivery systems pave the way for a new era of highly selective, minimally invasive treatments.

## Results

### Superparamagnetic nanoparticles are conjugated to LNA-21 via a thermolabile linker conjugate, enabling organ-specific delivery and controlled release

To achieve controlled and targeted RNA inhibition, we developed an innovative approach that covalently binds a well-defined miRNA inhibitor to SPIONs using a newly designed thermosensitive linker conjugate, which, based on the BoC group, allows the liberation of a secondary amine that induces release of the drug cyclization. The system has the advantage that the payload remains inactive during circulation but enables precise, on-demand release upon the application of an alternating magnetic field (AMF). The applied magnetic field induces hysteresis losses, resulting in localized heating of the SPION core and subsequent degradation of the Boc group^17^^,^^18^ and hence cleavage of the linker, enabling traceless release of the inhibitor or other coupled drugs at the target site. For proof of concept, we used a phosphorothioate-modified miRNA inhibitor known as locked nucleic acid (LNA), targeting the well-characterized pro-fibrotic miRNA-21.^20^

**Figure 1:**
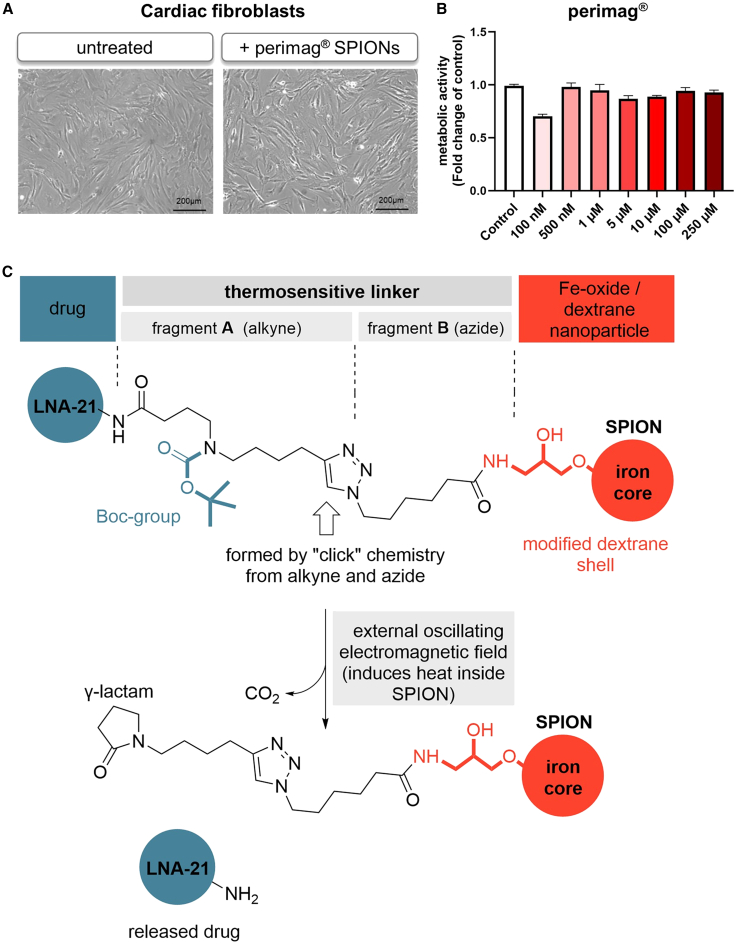
SPION-LNA-21 conjugate as organ-specific delivery system (A) Microscopic bright-field images were taken after treatment of human cardiac fibroblasts with Perimag (250 μM) SPIONs, and cellular morphology was evaluated after 24 h. Representative images from *N* = 3 independent experiments are shown. Scale bars, 200 μm. (B) WST-1 assay was performed after treatment of human cardiac fibroblasts with different doses of Perimag (100 nM, 500 nM, 1 μM, 5 μM, 10 μM, 100 μM, and 250 μM). One-way-ANOVA test. Data are presented as mean ± SEM; *n* = 3 independent experiments (C) Molecular architecture of the LNA-21/linker/perimag (SPION) conjugate and the thermally induced mechanism of LNA-21 release.

For the synthesis of the proposed SPION-LNA conjugate, we first searched for different types of SPIONs that might fulfill the following requirements: a) biocompatibility, b) ability to convert magnetic energy into heat, and c) possibility of functionalization. The surface functionalization of SPIONs provides binding sites for LNA attachment, with higher degrees of functionalization resulting in increased LNA-to-SPION ratios. The most promising SPION candidates were then tested *in vitro* for cell toxicity. The cellular morphology of cardiac fibroblasts remained unaltered, and the metabolic activity, as a measure of cellular integrity, was unaffected after treatment with different doses of Perimag (micromod) SPIONs (Figures 1A and 1B), confirming their biocompatibility. Perimag particles consist of an Fe_3_O_4_ core coated with a modified, amino-functionalized dextran shell, making them suitable for conjugation with LNA-21 via the thermosensitive linker system. Details on amino-modified dextran shells in SPIONs have been reported by Grüttner et al.^23^

To enable thermally triggered release, we modified LNA-21 by introducing a C6 building block with a terminal NH_2_ group at the 5′ end. Two different thermosensitive linker elements, named fragment A and fragment B, were synthesized separately to prevent chemoselectivity issues. These fragments were then individually conjugated to LNA-21 as well as to SPIONs before being fused using classical “click” chemistry (Figure 1C; synthesis is explained in detail in the supplemental methods section). Upon applying an AMF, localized heating triggers the traceless release of LNA-21 through thermal cleavage of the Boc protecting group, followed by intramolecular cyclization and lactam formation.

### SPION-LNA-21 conjugate is non-immunogenic and non-toxic

**Figure 2:**
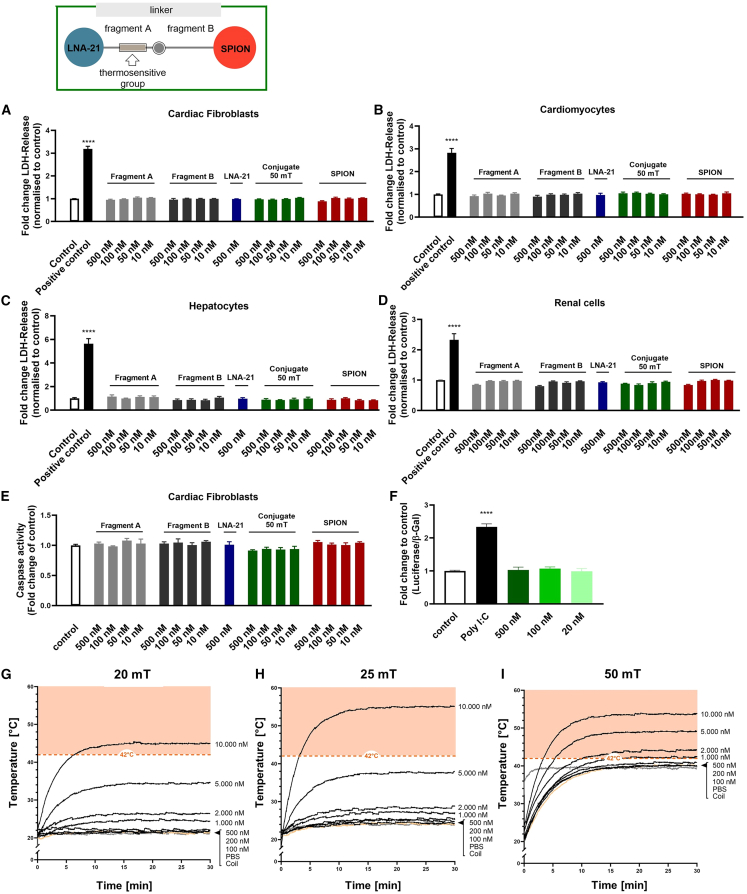
Safety analysis of the SPION-LNA-21 system and its individual components (A) Human cardiac fibroblasts, (B) neonatal mouse cardiomyocytes, (C) human liver cells (HepG2), and (D) rat kidney cells (NRK) were treated with different components of the conjugate system (SPIONs, Fragment A, Fragment B, SPION-LNA-21 conjugate + alternating magnetic field (AMF), 50 mT, 397 Hz) and LDH release and caspase 3/7 activity (E) were determined. For the negative control, cells were treated with PBS. For the positive control, cells were lysed 45 min before LDH release was measured. Data are presented as mean ± SEM; *n* = 3 independent experiments; one-way ANOVA with Tukey's multiple comparison test; (F) HEK293T cells were transfected with a bidirectional NF-κB-responsive reporter and treated with different concentrations of the SPION-LNA-21 conjugate, and luciferase activity was measured and normalized to β-Gal. For the positive control, cells were treated with 3 μg/mL poly I:C. *n* = 3 independent experiments. One-way ANOVA with Tukey's multiple comparison test;∗∗∗∗*p* ≤ 0.0001; ns = not significant. (G–I) The temperature profile of different doses of SPIONs in PBS was evaluated when different AMF field strengths (20, 25, and 50 mT, 397 Hz) were applied. Representative curves from independent experiments are shown.

In the next step, biocompatibility was assessed in cardiac fibroblasts and cardiomyocytes, two main cell types of the myocardium. Our results confirmed that all individual components of the conjugate, including both linker molecules, SPIONs, LNA-21, and the complete LNA-SPION conjugate, were non-toxic even at high concentrations. This was demonstrated by measuring LDH release (Figures 2A and 2B) and caspase activity (Figure 2E), both of which showed no significant increase. Importantly, exposure to an AMF and the resulting heating of the SPION core did not induce cellular damage (green bar). These findings were further validated in key off-target cells, specifically renal and liver cell types, which also exhibited no signs of cytotoxicity (Figures 2C and 2D).

To further evaluate the safety profile of our lead construct, we investigated its immunogenicity in preclinical studies. A key signaling pathway of proinflammatory cytokine expression is the nuclear factor kappa B (NF-κB) pathway. To assess whether our nanoparticle system triggered an immune response, we utilized a bi-directional NF-κB-responsive reporter.^24^ Our findings provided strong evidence that the SPION-based delivery system is not only well tolerated but also non-immunogenic, as indicated by the absence of NF-κB activation (Figure 2F).

For *in vivo* applications, it is essential that the bulk temperature remains within a biosafe range, not exceeding 42°C. To determine thermal safety, we measured the temperature of the construct in PBS at a concentration mimicking physiological conditions in the bloodstream (5,000 nM SPION-LNA-21 conjugate). Our results showed that field strengths up to μ0H = 25 mT maintained the temperature within the biosafe range. However, exposure to a higher SPION concentration or an increased magnetic field strength led to excessive heating, posing a risk of organ damage (Figures 2G–2I). These findings establish a clear framework for safe application parameters, ensuring that our system remains both effective and non-harmful under physiological conditions.

### LNA-21 can be efficiently released after application of an AMF

**Figure 3:**
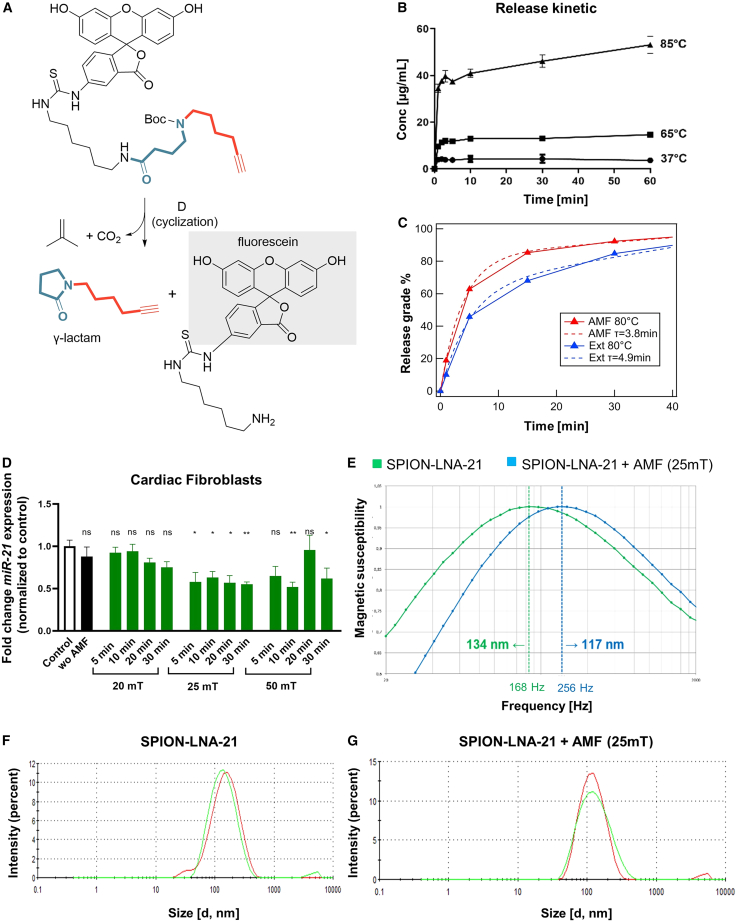
Release kinetics of the SPION-LNA-21 conjugate (A) Cyclization products of the test substrate FITC bound to the 4-(hex-5-yn-1-ylamino)butanoyl group and fragment A, analogous to the SPION-LNA-21 conjugate. After conventional heating, the products were detected by mass spectrometry, and the efficiency of the released fluorescein-bearing fragment (B) was measured at different temperatures (37°C, 65°C, and 85°C) *n* = 3 independent experiments. Data are presented as mean ± SEM. (C) The release grade of LNA-21 from the SPIONs was measured by magnetic particle spectroscopy, comparing magnetic heating (alternating magnetic field [AMF], red) and external heating (Ext, blue). *N* = 5 averages. (D) Human cardiac fibroblasts were treated with SPION-LNA-21 conjugate (200 nM) and subjected to different field strength (μ0H = 20, 25, 50 mT, 397 Hz, green) in an AMF to release the LNA. miRNA-21 expression levels were measured after 48 h via RT-qPCR. Data are presented as mean ± SEM, *n* = 3 independent experiments; one-way ANOVA with Dunnett’s multiple comparison test; ∗*p* ≤ 0.05; ∗∗*p* ≤ 0.01. (E) The hydrodynamic diameter of the SPION-LNA-21 conjugate was estimated from the characteristic frequency (omega∗tau = 1) in AC susceptibility measurements prior to heating (green) or after application of an AMF (μ0H = 25 mT, 397 Hz, blue) *N* = 10. (F and G) DLS (dynamic light scattering) measurements evaluated differences in hydrodynamic diameter without AMF and after application of an AMF (μ0H = 25 mT, 397 Hz). Representative images from *n* = 3 independent experiments are shown.

After validating the safety aspects, we next focused on examining the controlled release of LNA-21 from the SPIONs in more detail. Since direct detection of the released LNA by mass spectrometry was not feasible, we developed a test substrate designed to mimic the original system. This substrate included a C6 building block with a terminal amine and a Boc-protected secondary amine, serving as the “target breakpoint” analogous to the bound LNA (Figure 3A). To enable detection, the test substrate was synthesized with fluoresceine isothiocyanate (FITC). We demonstrated that thermally induced release was most efficient at 85°C, achieving a release efficiency of 81%, while the conjugate system remained stable under physiological conditions (Figure 3B). Importantly, this does not imply that the aqueous suspension of the conjugate is exposed to such elevated temperatures; rather, the localized heating in the immediate vicinity of the SPION core is sufficient to trigger linker activation, while the surrounding medium remains at physiologically safe levels. To further investigate release kinetics, we examined the original SPION-LNA-21 conjugate using magnetic particle spectroscopy (MPS) (Figure 3C), comparing magnetic heating with external heating through the medium. Our results showed that magnetic heating led to a significantly increased and accelerated LNA-21 release, suggesting that the magnetic SPION core undergoes localized overheating, thereby activating the thermo-linker more efficiently than uniform, external heating. To determine the specific field strength required for effective LNA-21 release, cardiac fibroblasts were transfected with varying doses of the SPION-LNA conjugate, followed by the application of an AMF at different field levels (Figure 3D), revealing that a minimum field strength of μ0H = 25 mT was required to successfully release LNA-21 and inhibit miR-21 expression. Additionally, we confirmed that the LNA remained inactive while covalently bound to the SPION. The release of LNA-21 at 25 mT was further validated by AC susceptibility (ACS) data (Figure 3E) and dynamic light scattering measurements (Figures 3F and 3G), which demonstrated a distinct change in (hydrodynamic) diameter before and after AMF exposure. These findings establish that controlled and localized heating via magnetic field application is a highly effective mechanism for precise, on-demand RNA delivery.

### SPION-LNA-21 conjugate is safe in *in vivo* settings

**Figure 4:**
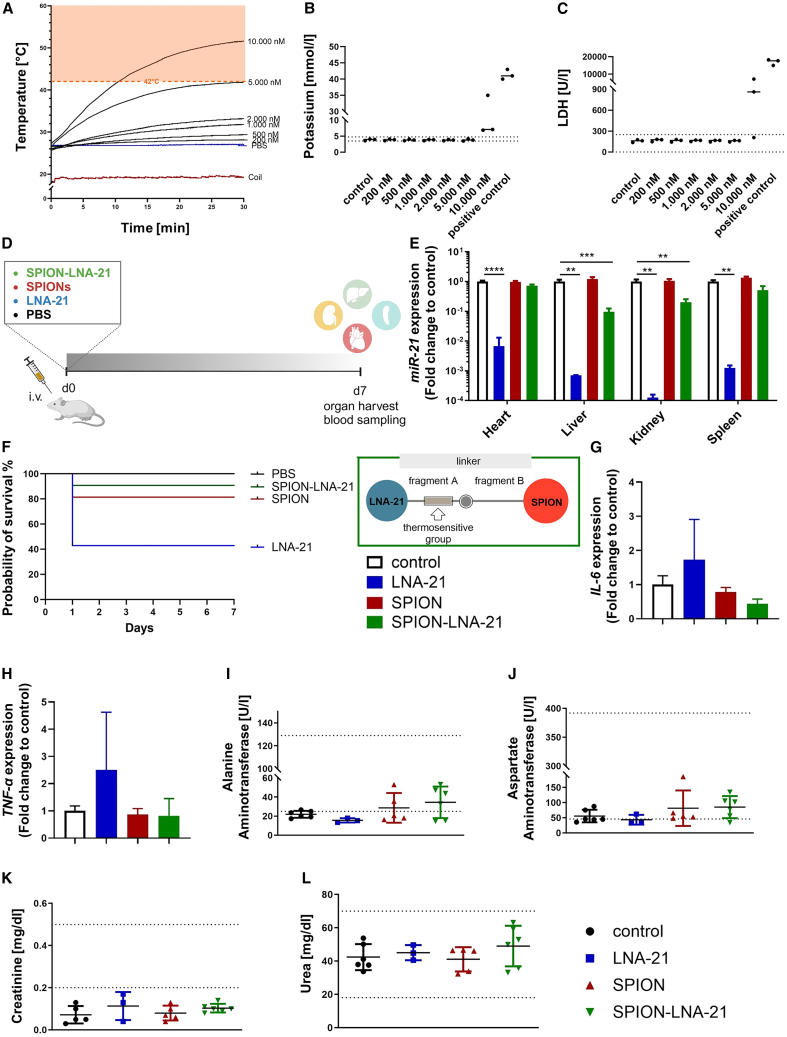
Proof-of-principle *in vivo* study (A) Human blood was spiked with different doses of SPIONs, and an alternating magnetic field (AMF) was applied. The temperature profile and (B and C) serum concentrations of potassium and LDH were evaluated. *N* = 3 independent experiments. (D) Experimental design of the *in vivo* study. Mice were intravenously injected with PBS, LNA-21 (20 mg/kg body weight), SPIONs (5 mg iron), and SPION-LNA-21 conjugate (5 mg iron), and organs were harvested after 7 days. (E) miRNA-21 expression was measured in different organs 7 days post-injection. Data are presented as mean ± SEM, *N* = 7 (PBS group), *n* = 5 (SPION group), *n* = 6 (SPION-LNA-21 group), and *n* = 3 (LNA-21 group); one-way ANOVA with Bonferroni's multiple comparison test. (F) The probability of survival was analyzed via a Kaplan-Meier survival curve. Inflammatory markers such as interleukin-6 (G) and tissue necrosis factor alpha (TNF-α) (H)) were measured via qRT-PCR in heart tissue after 7 days. *N* = 7 (PBS group), *n* = 5 (SPION group), *n* = 6 (SPION-LNA-21 group), and *n* = 3 (LNA-21 group). One-way ANOVA with Dunnett's multiple comparison test. Data are presented as mean ± SEM. Plasma concentrations of liver (ALT = alanine aminotransferase [I], AST = aspartate aminotransferase [J)]) and kidney failure (creatinine [K], urea [L]) were measured via the Cobas 8000 system. *N* = 6 (PBS group), *n* = 5 (SPION group), *n* = 6 (SPION-LNA-21 group), and *n* = 3 (LNA-21 group), Data are presented as mean ± SEM; ∗∗*p* ≤ 0.01, ∗∗∗*p* ≤ 0.001; ∗∗∗∗*p* ≤ 0.0001.

After confirming the tolerability of the individual components of the nanoparticle system *in vitro*, we next investigated their behavior in human blood. To assess safety under physiological conditions, different doses of the SPION-LNA conjugate were mixed with human blood, followed by the application of an AMF for 30 min (Figure 4A). Consistent with our previous findings, a concentration of 5,000 nM of SPION-LNA-21 conjugate remained within the biosafe range, with key blood parameters, such as potassium and LDH levels, remaining stable and within normal limits (Figures 4B and 4C). These results further support the biocompatibility of the system, even under simulated *in vivo* conditions.

The first *in vivo* mouse study served as a proof-of-principle to evaluate the safety and tolerability of our nanoparticle construct at the highest feasible intravenous dose, limited by the lethal threshold of iron oxide in SPIONs.^13^^,^^14^^,^^16^ To assess toxicity, mice were intravenously injected with the SPION-LNA-21 conjugate (5 mg iron), linker-free SPIONs (5 mg iron), LNA-21 at a dose previously published to be effective (20 mg/kg body weight^20^^,^^22^), or PBS as a control, and organs were harvested after 7 days (Figure 4D). While administration of pure LNA-21 resulted in an increased mortality rate, conjugation to SPIONs significantly reduced toxicity, indicating that even at the highest iron content, the nanoparticle construct was well tolerated (Figure 4F). Further assessment of kidney and liver function showed that key blood parameters, including AST (aspartate aminotransferase), ALT (alanine aminotransferase), creatinine, and urea levels, remained within the normal range (Figures 4I–4L). Additionally, cardiac gene expressions of inflammatory markers, such as *IL-6* (interleukin-6) and *TNF-α* (tissue necrosis factor-alpha) were not elevated (Figures 4G and 4H), further supporting the safety of the nanoparticle system.

To evaluate the biodistribution and functional impact of systemic administered LNA-21, *miR-21* expression levels were quantified in all major organs. The results demonstrated that systemic injection of LNA-21 led to widespread suppression of miR-21 across multiple organs, emphasizing the necessity for a more target-oriented delivery approach to enhance cardiac specificity (Figure 4E). As reported in the literature, antisense oligonucleotides like LNAs tend to accumulate primarily in the liver and kidneys,^25^ with only a smaller fraction reaching the heart, posing a challenge for cardiac-targeted therapies. To investigate the biodistribution of our SPION-LNA conjugate, we performed Perl's Prussian blue staining on histological sections, confirming notable off-target accumulation, particularly in the liver and spleen (Figure S1). These findings confirm the safety of the SPION-LNA-21 construct while underscoring the importance of optimizing organ-specific RNA delivery.

### SPION-LNA-21 conjugate enhances drug accumulation at the target site while reducing systemic off-target effects

**Figure 5:**
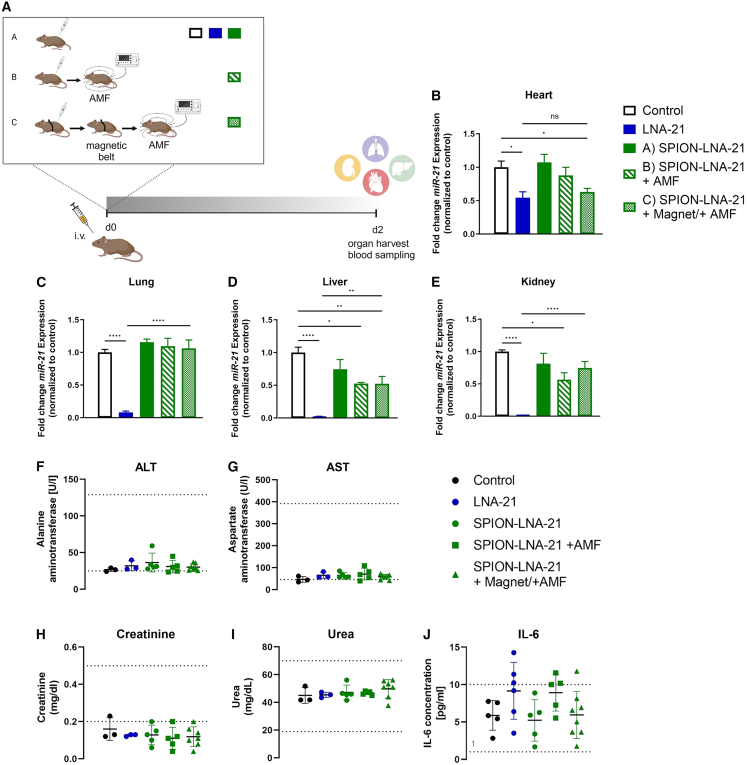
*In vivo* efficacy study (A) Study design of the *in vivo* study. Mice were either intravenously injected with PBS, LNA-21 (2.5 mg/kg body weight), or SPION-LNA-21 conjugate (2.5 mg/kg body weight) (group A); injected with SPION-LNA-21 conjugate (2.5 mg/kg body weight) followed by application of an alternating magnetic field (AMF, 25 mT, 397 Hz; group B); or injected with SPION-LNA-21 conjugate (2.5 mg/kg body weight) and treated with a combination of an external magnetic belt on the heart during injection and subsequent application of an AMF (group C). Organs were harvested after 2 days, and miRNA-21 expression levels were measured in the heart (B), lung (C), liver (D), and kidney (E). *N* = 6 animals in the PBS group, *n* = 6 animals in the LNA-21 group, *n* = 5 animals in the SPION-LNA-21 group, *n* = 5 animals in the SPION-LNA-21 + AMF group, and *n* = 8 animals in the SPION-LNA-21 +magnet +AMF group. Data are presented as mean ± SEM. One-way ANOVA with Tukey multiple comparison test. Plasma concentrations of liver markers (ALT = alanine aminotransferase [F], AST = aspartate aminotransferase [G]), kidney function markers (creatinine [H] and urea [I]), as well as the inflammatory marker interleukin-6 (Il-6) (J), were measured*. N* = 3–4 animals in the PBS group, *n* = 3 animals in the LNA-21 group, *n* = 5 animals in the SPION-LNA-21 group, *n* = 5 animals in the SPION-LNA-21 + AMF group, and *n* = 7 animals in the SPION-LNA-21 +magnet +AMF group. ∗*p* ≤ 0.05; ∗∗*p* ≤ 0.01; ∗∗∗*p* ≤ 0.001; ∗∗∗∗*p* ≤ 0.0001; ns = not significant.

Building on our previous findings, we next aimed to validate the efficient release of LNA-21 from the SPIONs *in vivo* and assess its effect on *miR-21* expression across different organs. To improve targeted delivery and evaluate release efficacy, we designed three experimental groups for the study: (A) intravenous injection of the SPION-LNA-21 construct without applying an AMF, ensuring that the LNA remained bound and inactive; (B) intravenous injection followed by AMF application to induce the release of LNA-21; and (C) a combination approach in which a magnetic belt was placed over the beating heart during injection and for an additional 30 min to attract the SPION-LNA-21 construct to the cardiac region, followed by AMF-triggered release (Figure 5A). Two days post-injection, organs were harvested, and *miR-21* expression was quantified as an indicator of successful LNA-21 release. Consistent with our previous findings, systemic injection of pure LNA-21 (blue column, Figures 5B–5E) resulted in widespread off-target effects across multiple organs, with *miR-21* expression in the heart reduced by only 50%. Notably, the combination of the external magnet and AMF (group C, green columns) significantly minimized off-target accumulation, particularly in the lungs, liver, and kidneys, while maintaining miR-21 suppression in the heart at levels comparable to pure LNA injection. These findings highlight the potential of magnetic field-assisted targeting as a promising strategy for cardiac-specific RNA delivery, while reducing systemic distribution and off-target effects.

To assess the biocompatibility of AMF for *in vivo* applications, we monitored survival rates and key blood parameters. All mice survived the procedure, and blood markers for kidney and liver function remained within the biosafe range (Figures 5F–5I), confirming that AMF exposure did not induce systemic toxicity. Interestingly, plasma IL-6 concentrations were slightly elevated following the injection of pure LNA-21. However, this effect was completely abolished when LNA-21 was conjugated to SPIONs and remained absent even after its release via AMF (Figure 5J). In line with these results, cardiac levels of inflammatory markers *Il1b* and *TNF-a* were not elevated after application of an AMF (Figures S2A and S2B).

### SPION-LNA-21 conjugate efficiently reduced cardiac fibrosis and hypertrophy

**Figure 6:**
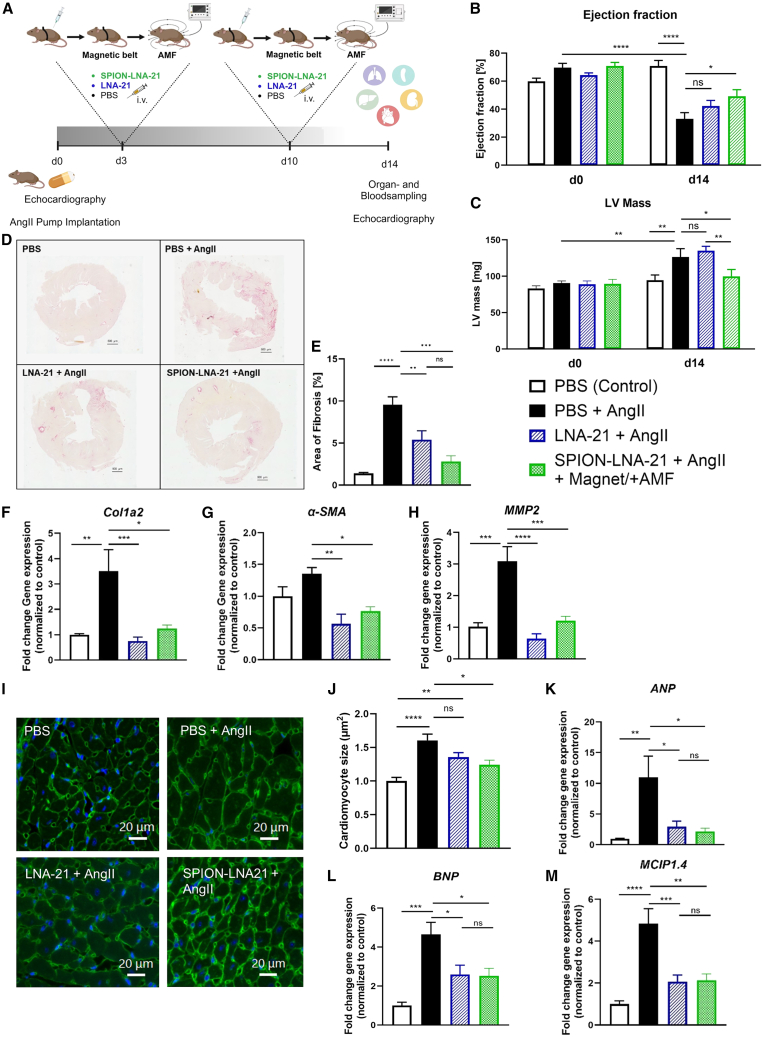
Evaluation of therapeutic effects in the angiotensin-II mouse model (A) Study design of the *in vivo* study. On day 0, an osmotic pump was subcutaneously implanted to constantly release angiotensin-II (3 mg/kg/day) over a period of 14 days. Control mice were sham-operated. On day 3 and 10, mice were intravenously injected with PBS, LNA-21 (2.5 mg/kg body weight), or SPION-LNA-21 conjugate (2.5 mg/kg body weight), with a magnetic belt placed on the heart during injection and subsequent application of an alternating magnetic field (AMF, 25 mT, 397 Hz) to release the LNA. Echocardiographic parameter, s ejection fraction (B) and left ventricular (LV) mass (C), were measured on day 0 and day 14 and then calculated. Data are presented as mean ± SEM; *N* = 6 in the PBS group, *n* = 7 in the PBS+AngII group, *n* = 9 in the LNA-21+AngII group, and *n* = 5 in the SPION-LNA-21 + AngII group. One-way-ANOVA with Sidak’s multiple comparison test. (D) Microscopic images of Picro-Sirius Red staining (PSR) on heart sections. Representative images from *n* = 5–9 animals per group. (E) Quantification of fibrosis levels in heart sections stained with PSR. Data are presented as mean ± SEM; one-way ANOVA with Tukey's multiple comparison test; *N* = 6 in the PBS group, *n* = 7 in the PBS+AngII group, *n* = 9 in the LNA-21+AngII group, and *n* = 5 in the SPION-LNA-21 + AngII group. Gene expression of fibrosis-associated genes, collagen 1a2 (Col1a2) (F), α-smooth muscle actin (a-SMA) (G), and matrix metalloproteinase 2 (MMP2) (H), was measured. Data are presented as mean ± SEM; one-way ANOVA with Tukey's multiple comparison test; *N* = 6 in the PBS group, *n* = 7 in the PBS+AngII group, *n* = 9 in the LNA-21+AngII group, and *n* = 5 in the SPION-LNA-21 + AngII group. (I) Confocal images of wheat-agglutinin (WGA) staining on heart sections. Representative images from *n* = 5–9 animals per group. (J) Quantification of cardiomyocyte size in heart sections stained with WGA. Data are presented as mean ± SEM; one-way ANOVA with Tukey's multiple comparison test; *N* = 5–9 animals per group. Gene expression of hypertrophy-associated genes, ANP (atrial natriuretic peptide (K), BNP (brain natriuretic peptide) (L), and MCIP (myocyte-enriched calcineurin-interacting protein (M), was analyzed in heart tissue. Data are presented as mean ± SEM; one-way ANOVA with Tukey's multiple comparison test; *N* = 6 in the PBS group, *n* = 7 in the PBS+AngII group, *n* = 9 in the LNA-21+AngII group, and *n* = 5 in the SPION-LNA-21 + AngII group. ∗*p* ≤ 0.05; ∗∗*p* ≤ 0.01; ∗∗∗*p* ≤ 0.001; ∗∗∗∗*p* ≤ 0.0001; ns = not significant.

To assess whether the significant reduction in cardiac *miR-21* levels achieved with the SPION-LNA-21 construct translated into therapeutic anti-fibrotic efficacy, we examined biochemical and functional improvements in a mouse model of early heart failure with reduced ejection fraction (HFrEF). The disease model was established by pharmacologically inducing hypertension through subcutaneous implantation of an osmotic pump, which continuously released angiotensin II (Ang II) over 14 days. This chronic stimulation of the renin-Ang-aldosterone system (RAAS) progressively led to myocardial hypertrophy and fibrosis, mimicking pathological cardiac remodeling. On the third and tenth days after pump implantation, mice were either treated with pure LNA-21 or with the SPION-LNA-21 conjugate in combination with AMF and a magnetic belt (Figure 6A).

After 14 days, organs were harvested to evaluate target engagement (Figures S3A–S3D). In the heart, both the SPION-LNA-21 construct and native LNA-21 significantly reduced *miR-21* expression to physiological levels. However, animals treated with pure LNA-21 exhibited a drastic reduction of *miR-21* in off-target organs, resembling a systemic miR-21 knockdown effect. In contrast, the magnetically targeted nanoparticle delivery system significantly reduced *miR-21* expression in the heart while limiting off-target effects. No significant reduction was observed in the spleen (Figure S3B), and only minor decreases were detected in the liver (Figure S3C) and kidneys (Figure S3D), likely due to the pharmacokinetic degradation of residual nanoparticles in these organs. Overall, intracellular *miR-21* expression in off-target organs was reduced by 60.7% in the liver, 67.1% in the kidney, and 98.8% in the spleen, while cardiac *miR-21* expression was completely normalized, demonstrating the efficacy of this magnetic drug delivery system in minimizing off-target effects. Echocardiographic assessment revealed a significant improvement in cardiac function (ejection fraction) following treatment with the SPION-LNA-21 conjugate (Figure 6B). Additionally, left ventricular hypertrophy was prevented only by the nanoparticle conjugate (Figure 6C).

To evaluate the therapeutic impact of this targeted approach, histological analysis of heart tissue revealed that treatment with the SPION-LNA-21 conjugate resulted in an even greater reduction in fibrosis, restoring it to physiological levels more effectively than native LNA-21 treatment (Figures 6D and 6E). This was supported by a significant reduction in fibrosis-associated genes, including *Col1a2, α-SMA*, and *MMP2* (Figures 6F–6H). Myocyte diameter measurements further confirmed that the SPION-LNA-21 conjugate significantly reduced cardiac hypertrophy, whereas native LNA-21 treatment had only a limited effect (Figures 6I and 6J). Consistently, hypertrophy markers *MCIP1.4, BNP*, and *ANP* were also significantly reduced in the SPION-LNA-21-treated group (Figures 6K–6M).

To rule out potential systemic toxicity, plasma markers of organ damage were analyzed, including ALT, AST, creatinine, and urea (Figures S4A–S4D), which remained within the normal range across all treatment groups. Immune response analysis revealed no elevation in C-reactive protein (CRP) levels in treated animals compared to controls or the reference range (Figure S4E), suggesting a low likelihood of chronic inflammation. Whereas all animals survived in the SPION-LNA-21 conjugate group, the probability of survival was reduced in the LNA-21 group (Figure S4F), underlining the safety of this nanoparticle system.

Our findings suggest that the key therapeutic advantage of the SPION platform lies in its ability to spatially control drug release while minimizing systemic exposure, rather than in achieving higher levels of cellular uptake in the target tissue. Moreover, this targeted approach significantly minimizes off-target effects while maintaining an excellent safety profile, highlighting its potential as a novel therapeutic strategy for cardiac fibrosis and other diseases requiring precise RNA-based interventions.

## Discussion

In this study, we successfully developed a novel, stable, and functional drug delivery system based on an SPION-linker-LNA conjugate. This system addresses key challenges that have hindered broader clinical translation of RNA therapeutics, including organ specificity, off-target effects, and immune responses. By coupling a therapeutic LNA of a well-characterized miRNA to the surface of SPIONs via a thermocleavable linker, we achieved active, externally controlled drug release. The application of an AMF induces localized heating, cleaving the linker and enabling on-demand release of LNA-21.

Previous studies have shown that pharmacological inhibition of miRNA-21 reduces fibrosis in multiple organs, such as the heart,^20^^,^^22^^,^^26^ lung,^27^ liver,^28^ or kidney.^29^^,^^30^^,^^31^ One key advantage of LNAs is their stability and ability to function without the need for transfection reagents, as demonstrated by Buntz et al.^32^ However, systemic administration of miRNA-21 inhibitors is not organ-specific and, like most antisense oligonucleotides, predominantly accumulates in metabolic organs such as the liver and kidneys.^25^ This highlights the urgent need for a targeted drug delivery system to overcome these limitations.

Here we demonstrated that AMF application efficiently triggers LNA release and that all components of the system, including the end products after cleavage, are well tolerated within therapeutic dose ranges in both *in vitro* and *in vivo* settings. Although the maximum permissible iron dose for *in vivo* applications remains a limiting factor, given the currently achieved quantity of bound RNA on the SPIONs, we observed no signs of iron-related toxicity at the dose levels used in this study.^13^^,^^14^^,^^16^ Importantly, SPION-mediated localized heating remained below the biosafe threshold of 42°C at the maximum applicable iron concentration. This moderate temperature increase exhibited no cytotoxic effects on blood cells *in vitro*. Notably, while the SPION core reached localized temperatures of approximately 80°C to enable linker cleavage, efficient heat dissipation into the surrounding aqueous environment effectively mitigated thermal risk to adjacent tissues.

A key advantage of our system is its ability to enhance drug accumulation at the target site while reducing systemic off-target effects or progressive accumulation in organs such as the liver or kidneys. Indeed, systemic injection of unbound LNA-21 led to broad suppression of miR-21 across multiple organs, reinforcing the need for a more targeted approach. The use of an external magnet to guide the SPION-LNA-21 conjugate to the heart, followed by AMF application, significantly improved cardiac specificity and minimized off-target effects. This method significantly decreased the reduction of miR-21 expression in non-cardiac organs, while achieving complete normalization of miR-21 levels in the heart.

In a therapeutic *in vivo* setting, the SPION-based approach and naked LNA resulted in comparable levels of miR-21 knockdown in the heart, while the SPION system led to improved physiological outcomes, including reduced cardiac fibrosis and hypertrophy. This apparent discrepancy may be explained by the distinct pharmacokinetic profiles and the precise control of LNA release afforded by the SPION platform. The SPION system minimizes systemic exposure and off-target knockdown, ensuring that LNA-21 is predominantly active in the myocardium, where it can exert its therapeutic effects. In contrast, systemic LNA administration leads to widespread miR-21 inhibition across multiple organs, which, while effective in the heart, may also elicit off-target effects in non-cardiac tissues, potentially blunting beneficial adaptive responses. The ability of the SPION system to focus therapeutic activity in the target tissue while minimizing adverse effects in other organs could therefore explain the observed improvements in cardiac function and structural remodeling. This highlights the importance of not only achieving target knockdown but also optimizing the spatiotemporal control of drug delivery for improved therapeutic efficacy.

The SPION-based system offers a promising advancement in controlled RNA delivery, particularly through its ability to reduce systemic exposure and enhance targeted release. While it may not yet outperform lipid-based systems in terms of on-target uptake, its precise control over drug activation could provide a unique therapeutic advantage, particularly for conditions requiring spatiotemporal precision. Further studies, including comparisons with established lipid-based systems, will be essential to fully characterize the advantages and limitations of this novel delivery platform.

Despite the advantages of this system, precise quantification of the LNA payload per SPION remains a methodological challenge due to the particle size of the conjugate, which precludes direct mass spectrometry analysis. In addition, the exact determination of the percentage of released drug in relation to the overall functionalization of the particle surface is still a general problem in the field. Further research is needed to explore whether fine-tuning the magnetic field strength and duration of AMF exposure could enable partial LNA release, allowing for more precise secondary dosing beyond the initial intravenous application.

The feasibility of magnetically guided nanoparticle heating has already been demonstrated in a mouse model by Tay et al.,^33^ who achieved selective heating of target regions while preserving adjacent healthy tissues. Future studies could incorporate magnetic particle imaging (MPI)^34^^,^^35^ to enable real-time visualization and control of localized heating, thereby ensuring highly precise LNA release within the region of interest.

While miRNA expression levels vary across tissues, miR-21 was intentionally chosen as a disease-relevant proof-of-concept target with well-established functional and translational significance in cardiac fibrosis. The focus of this study was therefore on demonstrating controlled, therapeutically effective target engagement rather than on transcript-agnostic biodistribution benchmarking. We acknowledge that ubiquitously expressed reference transcripts, such as the long non-coding RNA Malat1, are commonly used in the antisense field to assess tissue distribution independent of target biology. Future studies incorporating such benchmark targets will be important to further delineate the biodistribution characteristics and generalizability of the SPION-based delivery platform. Beyond cardiac applications, our actively controllable, SPION-based drug delivery platform holds broad therapeutic potential. By modulating the positioning of the external magnetic field, the concept can be readily adapted to target a range of organs and disease sites. Additionally, as long as the active agent possesses an amino or, in principle, an alcohol functional group, alternative therapeutics could be conjugated to the SPION-linker system, expanding its use beyond ncRNA inhibitors.

In summary, we have developed a first-of-its-kind, externally controllable, SPION-based drug delivery system that enables targeted RNA inhibition with minimized off-target effects. This innovative approach not only enhances therapeutic precision but also reduces systemic immune activation and toxicity, making it a promising candidate for clinical translation in RNA-based therapies.

## Materials and methods

### Magnetic characterization and drug release monitoring

#### ACS

ACS measurements were conducted to analyze the magnetic relaxation properties of the SPION conjugate. In ACS, an AMF is applied over a range of frequencies, and the complex susceptibility (*χ* = *χ*′+*iχ*″) is recorded. The real part (*χ*′) reflects the in-phase response, while the imaginary part (*χ*″) represents energy dissipation and peaks at the characteristic relaxation frequency$,\;f=\frac{\omega }{2\pi }$, of the particles (*ωτ* = 1). For particles primarily undergoing Brownian relaxation, the peak position in *χ*″ is linked to the Brownian relaxation time constant, *τ*_*B*_, which is given by $\tau_{B}=\frac{3\eta V_{h}}{k_{T}T}$, where η represents the dynamic viscosity of the medium, *V*_*h*_ is the hydrodynamic volume of the particle, *k*_*B*_ is the Boltzmann constant, and *T* is the absolute temperature. From this relation, the hydrodynamic diameter can be derived.

#### MPS

MPS was employed as a complementary technique to monitor changes in the magnetic relaxation behavior associated with drug release. MPS measures the nonlinear magnetization response of magnetic nanoparticles exposed to a high-amplitude AMF. In the low-frequency (LF) regime, typically below 1 kHz, MPS is particularly sensitive to changes in hydrodynamic size, similar to ACS. This makes MPS a suitable tool for monitoring drug release dynamics via relaxation-based signal changes, as described by Draack et al. *Drug-release study with MPS:* Samples were initially heated externally to 80°C using a Peltier-tempered sample holder directly in the MPS. Subsequently, an AMF of 25 kHz frequency and 25 mT amplitude was applied to induce hysteresis heating in the nanoparticles while keeping the sample solution at a constant temperature. This hysteresis heating mechanism is due to the irreversible rotation of magnetic moments in response to the AMF. At predetermined intervals, the AMF was briefly paused to perform LF-MPS measurements, enabling real-time tracking of changes in relaxation behavior to monitor drug release kinetics over time.

#### AMF

The AMF was generated using the TruHeat HF of the AXIO 10/450 series (Trumpf Hüttinger). The system is supported by a booster pump to ensure constant water pressure during operation. The following inductive coils are used to generate the magnetic field: (1) a coil with four windings and an internal diameter of 5.5 cm, for *in vivo* studies, and (2) a coil with six windings and an internal diameter of 3.0 cm, for *in vitro* studies.

Field strengths of 20, 25, and 50 mT (397 Hz) were used, as stated in the text.

### *In vitro* and *in vivo* experiments

#### Cell culture, transfection, and treatment

Human cardiac fibroblasts (HCFs, Promega) were cultured in fibroblast basal medium (FBM)-3 supplemented with 10% fetal bovine serum (FBS), supplements (Promega), 100 μg/mL penicillin, and 100 μg/mL streptomycin under standard cell culture conditions (37°C, 5% CO_2_). For analysis of release kinetics, the SPION-LNA-21 conjugate was subjected to an AMF at different field strengths for different durations, and HCF cells were afterward transfected with different doses of the nanoparticle construct (100 and 200 nM), the respective amount of unbound LNA-21 and unheated SPION-LNA-21 for 48 h.

NRK49F cells were cultivated in low-glucose DMEM (Lonza, Basel, Switzerland) supplemented with 10% FBS, 100 μg/mL penicillin, and 100 μg/mL streptomycin under standard cell culture conditions (37°C, 5% CO_2_). Neonatal mouse cardiomyocytes (NMCMs) were kept in MEM with 5% FBS at 37°C and 1% CO_2_. HepG2 cells were cultivated in RPMI medium (Gibco) with 10% FBS under standard cell culture conditions (37°C, 5% CO_2_).

#### Cytotoxicity assessment

The WST-1 assay (Roche) was used to measure the metabolic activity of HCF cells after treatment with Perimag SPIONs, according to the manufacturer’s instructions.

The CytoTox 96 Non-Radioactive Cytotoxicity Kit (Promega) was used to analyze LDH release in HCF, NRK49F, NMCM, and HepG2 cells after treatment with different doses of SPIONs, linker fragments, and SPION-LNA-21 conjugate (+AMF) for 48 h, according to the manufacturer’s instructions.

Using the Caspase-Glo 3/7 Assay (Promega), the apoptosis rate was assessed in HCF cells after treatment with different doses of SPIONs, linker fragments, and SPION-LNA-21 conjugate (+AMF) for 48 h, according to the manufacturer’s instructions.

#### RNA isolation

Total RNA from tissues and cultured cells was isolated using the RNeasy Mini Kit (Qiagen) or the Qiazol method (Qiagen), according to the manufacturer’s instructions. Subsequent quantification and quality control were performed with the Synergy HT Reader (BioTek).

#### Quantitative real-time PCR

For cDNA synthesis, the TaqMan MicroRNA Reverse Transcription Kit (Applied Biosystems) was used according to the manufacturer’s instructions. Quantification of specific miRNA levels was performed by quantitative real-time PCR using the TaqMan MicroRNA Assay (Applied Biosystems). qPCR was performed in a 384-well PCR plate with the ViiA 7 Real-Time PCR System (Thermo Fisher Scientific). The analysis was performed using the ΔΔct method. miRNA levels were normalized to the small RNA molecule snoRNA-202 and U6 for mouse samples and to RNU48 for human samples. The Taqman assays used are listed in Table 1.

**Table 1:** Sequences of LNA, primers, and TaqMan assays

**LNA Sequence**	

LNA-21	5′-/5AmMC6/TCAGTCTGATAAGCT-3′

**TaqMan Primer assays**	

*Rnu48*	ID: 001006
*U6 snRNA*	ID: 001973
*Sno202*	ID: 001232
*miR-21*	ID: 000397

**Primer Sequences**	

*mmu_GAPDH*	Forward:5′TTCACCACCATGGAGAAGGC3′; Reverse:5′GGCATGGACTGTGGTCATGA3′
*mmu_18S*	Forward: 5′ GTAACCCGTTGAACCCCATT 3′; Reverse: 5′CCATCCAATCGGTAGTAGCG 3′
*mmu_aSMA*	Forward:5′ACTACTGCCGAGCGTGAGAT3′; Reverse:5′AAGGTAGACAGCGAAGCCAG3′
*mmu_ANP*	Forward:5′CCTGTGTACAGTGCGGTGTC3′; Reverse:5′CCTAGAAGCACTGCCGTCTC3′
*mmu_BNP*	Forward:5′CTGAAGGTGCTGTCCCAGAT3′; Reverse:5′GTTCTTTTGTGAGGCCTTGG3′
*mmu_col1a2*	Forward:5′CAGAACATCACCTACCACTGCA3′; Reverse:5′TTCAACATCGTTGGAACCCTG3′
*mmu_Il-1b*	Forward:5′TGCCACCTTTTGACAGTGATG3′; Reverse:5′ATGTGCTGCTGCGAGATTTG3′
*mmu_Il-6*	Forward: 5′ AGCCAGAGTCCTTCAGAGAGAT 3′; Reverse: 5′ GAGAGCATTGGAAATTGGGGT 3′
*mmu_MCIP*	Forward:5′AGGGACTTTAGCTACAATTT 3′; Reverse:5′TATGTTCTGAAGAGGGATTC 3′
*mmu_MMP2*	Forward:5′GCCTCATACACAGCGTCAATCTT3′; Reverse:5′CGGTTTATTTGGCGGACAGT3′
*mmu_TNF-a*	Forward:5′TACTGAACTTCGGGGTGATTGGTCC3′; Reverse:5′CAGCCTTGTCCCTTGAAGAGAACC3′

For quantitative detection of mRNAs, reverse transcription of total RNA prior to real-time qPCR was performed using the iScript Select cDNA synthesis kit (Bio-Rad), according to the manufacturer’s instructions. Real-time qPCR was performed in a CFX96 Touch Real-Time PCR Detection System (Bio-Rad) using specific primers (Table 1) and the iQ SYBR Green Mix (Bio-Rad), according to the manufacturer’s protocol. Glyceraldehyde-3-phosphate dehydrogenase (GAPDH) or 18S ribosomal RNA (18S rRNA) was used as a housekeeping control for gene-specific expression levels.

#### IL-6 ELISA

For the quantification of IL-6 levels in mouse plasma, the Mouse IL-6 high sensitivity ELISA (Invitrogen) was performed according to the manufacturer’s instructions.

#### Dynamic light scattering

The hydrodynamic diameter of SPION-LNA-21 conjugates was characterized using dynamic light scattering (Zetasizer Nano ZS, Malvern Panalytical).

#### Statistics

All *in vitro* experiments were performed as indicated in the corresponding figure legends. If not labeled separately, three biological replicates were used for each independent experiment. Of these, three technical replicates were performed. Data are expressed as the mean of the independent samples ±standard error of the mean (SEM). Statistical analysis was performed using GraphPad Prism. For the statistical comparison of two groups, an unpaired, two-tailed Student’s *t* test was performed. For the comparison of three or more groups, a one-way ANOVA followed by the stated post-test was performed.

#### Animal experiments

All animal studies involving mice were performed in accordance with the relevant guidelines and regulations and with the approval of the Niedersächsisches Landesamt für Verbraucherschutz und Lebensmittelsicherheit (LAVES, Germany, TVA-ID 21/03636 and 2022/294). For all *in vivo* experiments, male C57BL/6NCrl wild-type mice (Charles River Laboratories, Germany) aged 8–10 weeks were used.

For the proof-of-principle study, mice were intravenously injected with PBS, 5 mg of Perimag SPIONs, 5 mg of SPION-LNA-21 conjugate, or LNA-21 (20 mg/kg body weight), and organs were harvested after 7 days.

For the efficacy study, mice were either intravenously injected with PBS, LNA-21 (2.5 mg/kg body weight), or SPION-LNA-21 conjugate (2.5 mg/kg body weight with respect to LNA-21 concentration) (group A); intravenously injected with SPION-LNA-21 conjugate (2.5 mg/kg body weight with respect to LNA-21 concentration), followed by application of an AMF (25 mT, 397 Hz for 30 min; group B); or injected with SPION-LNA-21 conjugate (2.5 mg/kg body weight with respect to LNA-21 concentration) and treated with a combination of an external magnetic belt placed on the heart during injection, and for a further 30 min, followed by subsequent application of an AMF (25 mT, 397 Hz for 30 min; group C). Organs were harvested after 2 days.

To induce systemic hypertension for the therapeutic study, subcutaneous implantation of osmotic minipumps (ALZET) delivering Ang II at 3 mg/kg BW/day was performed. The operation was performed under general anesthesia with isoflurane (2%–4% isoflurane in 0.8 L/min oxygen). After subcutaneous infiltration analgesia with lidocain and bupivacain (each 2 mg/kg BW), the minipump (ALZET micro-osmotic pump model 1002) was implanted in a subcutaneous pouch on the back. Analgesia was complemented with four carprofen injections (10 mg/kg BW) before surgery and 12, 24, and 48 h after operation. On the third and tenth day after pump implantation, mice were either treated with pure LNA-21 (2.5 mg/kg body weight) or with the SPION-LNA-21 conjugate (2.5 mg/kg body weight with respect to LNA-21 concentration). To enhance cardiac targeting, a neodymium permanent magnet was placed over the heart for 30 min during and after injection to enrich the nanoparticles in the myocardium before exposure to an AMF (25 mT, 397 Hz) for 30 min. Control animals received PBS injections, as well as sham-operated controls.

Cardiac function was assessed by echocardiography (Vevo2100, Fujifilm/VisualSonics, Canada) under general anesthesia with isoflurane (see above). Echocardiography data were analyzed using standard imaging protocols (M-mode and B-mode) for global cardiac volumes and function using Vevo LAB 3.2.0 (Fujifilm Visualsonics, Inc).

#### Isolation of neonatal cardiomyocytes from mice

To isolate cardiomyocytes, hearts from 0.5- to 2-day-old mice were removed and washed twice in PBS (with added 100 U/mL Pen/Strep). After transferring up to 40 minced mouse hearts into a gentleMACS C tube (Miltenyi Biotec), two additional washes with PBS were performed. Two enzyme mixtures, enzyme mixture 1: 125 μL enzyme P + 4600 μL buffer X, and enzyme mixture 2: 50 μL buffer Y + 25 μL enzyme A + 200 μL enzyme D, were mixed together and transferred to the gentleMACS C Tube, and the tissue was dissociated via the gentleMACS Octo Dissociator (Miltenyi Biotec). Next, the tubes were removed, and culture medium (MEM [Bioconcept], 5% FBS [Gibco], 292 mg/L L-glutamine [Sigma-Aldrich], 350 mg/L NaHCO_3_ [Sigma-Aldrich], 1 mL/L vitamin B12 [Sigma-Aldrich], 5 mL BrdU [Sigma-Aldrich], and 10 mL P/S [Promocell]) was added. The suspension was homogenized and transferred to a 70 μm MACS SmartStrainer. After washing, the cells were centrifuged at 600 × g for 5 min at room temperature. The supernatant was removed, and the cell pellet was resuspended in medium. The suspension was placed in a Petri dish and incubated for 90 min at 37°C and 1% CO_2_. During the incubation time, the required cell culture plates were coated with 0.1% gelatin (Sigma-Aldrich) and incubated for 45 min. Next, the cardiomyocytes were obtained from the supernatant. After further washing the Petri dish with medium, the cells were seeded onto the plates.

#### Histology

For histological assessment of cardiac fibrosis in mice, paraffin-embedded sections of the LV were stained with Picro-Sirius Red, and the collagen content was calculated as the percentage of fibrotic areas in the heart. Quantifications of microscopic images were performed with the BZ-X800 Analyzer (Keyence).

For murine cardiomyocyte size measurement, cardiomyocyte cell membranes in the myocardium were visualized by wheat germ agglutinin staining coupled to Alexa Fluor 488 (Invitrogen). The area of cardiomyocytes was calculated using ImageJ Fiji.

For biodistribution analysis of SPIONs, iron content was stainend in liver, spleen, and heart tissue via Perl's Prussian blue staining according to the manufacturer's instructions (Abcam).

#### Plasma sampling and biochemical analysis

EDTA-plasma samples were drawn from the mice and centrifuged at 3000 × g for 10 min. The supernatant was stored at −80°C until analysis. Laboratory parameters were determined on a Cobas 8000 Modul c701and Cobas c111 using standard methods (Roche): creatinine, urea, ALT, and AST.

## Data and code availability

The data underlying this article are available in the article and in its online supplemental information.

## Acknowledgments

The authors acknowledge valuable discussions with Frank Ludwig and measurement support provided by Deike Hicken. Furthermore, the authors acknowledge DLS measurement support from Sedef Ersoy, WGA measurement support from Nila Stieber, NMR measurements by Linn Müggenburg and Jörg Fohrer, and and laboratory parameter measurement support from Lichtinghagen, Leifheit-Nestler, and MartinaThiele. This project received funding from the 10.13039/501100007601European Union’s Horizon 2020 research and innovation program under grant agreement no. 825670, named Cardioregenix (to T.T.) and financial support from the 10.13039/501100001659German Research Foundation under grant no. VI 892/4-1 (to T.V.). A patent application regarding the RNA delivery technology has been filed. T.T. is founder and CSO/CMO of Cardior Pharmaceuticals GmbH, a wholly-owned subsidiary of Novo Nordisk Europe A/S.

## Author contributions

T.T. and A.K. conceived the project. F.K. and T.T. designed the biological part of the experiments, and A.K. and G.D. designed the chemical part. The synthesis of the SPION-LNA-21 conjugate system was developed and performed throughout the project by K.H., whereas synthesis for the angiotensin study was performed by A.S. *In vitro/ex vivo* experiments were performed and analyzed by F.K. together with L.P.J.H., K.H., and S.G. F.K., L.P.J.H., K.H., J.B., G.B., K.J., and C.B. designed and performed the *in vivo* studies. F.K., L.P.J.H., and A.J. carried out the histological analysis. T.V. examined the magnetic properties of the SPION system (ACS/MPS) and provided the expertise to design the SPION subtype for the synthesis. A.G., S.T., and A.P. performed RNA isolations and qPCRs throughout the project. F.K., G.D., A.K., and T.T. supervised the research. F.K., K.H., L.P.J.H., and T.T. wrote the manuscript. All authors read and approved the final paper.

## Declaration of interests

T.T. is founder and CSO/CMO of Cardior Pharmaceuticals GmbH, a wholly owned subsidiary of Novo Nordisk Europe A/S.

## Supplemental information

Document S1. Figures S1–S14 and Supplemental methods

Document S2. Article plus supplemental information
