# Supplementary material for: Time- and spatially resolved LNA delivery via thermally controlled SPION technology
Source: Mol Ther Nucleic Acids. 2026 Mar 13;37(2):102902. doi: 10.1016/j.omtn.2026.102902 (PMC13068855; doi:10.1016/j.omtn.2026.102902)
Supplement: Document S1. Figures S1–S14 and Supplemental methods [file mmc1.pdf]

OMTN, Volume *37*

Supplemental information

Time- and spatially resolved LNA delivery via thermally controlled SPION technology

Franziska Kenneweg, Katharina Hempel, Lukas Philipp Joachim Höhne, Gerald Dräger, Jonas Blume, Thilo Viereck, Anastasia Stohwasser, Sonja Groß, Gwen Büchler, Karina Jansen, Malte Juchem, Christian Bär, Angelika Pfanne, Annette Just, Sabrina Thum, Anika Gietz, Andreas Kirschning, and Thomas Thum

Supplemental methods

Chemical syntheses:

General information

All materials and reagents were purchased from commercial distributors (Sigma Aldrich, tci, Alfa Aesar, Acros Organics, Carl Roth) and were not further purified unless otherwise stated. Specially prepared reagents were labelled as such. For LC-MS as well as preparative and semi-preparative HPLC, double-distilled water and 0.1 % formic acid were used. Reactions that had to take place in the absence of air and moisture were carried out using the Schlenk technique.

Dialysis

Dialysis of the aqueous reaction solutions was carried out in dialysis tubes of the Membra-Cel™ dialysis membrane type with an exclusion limit of 14,000 Daltons from Carl Roth. The dialysis took place over four to five days, depending on the test specification, at room temperature and with gentle stirring in 5 litre beakers.

Chromatography

Silica gel 60M (particle size 40-63 µm) was purchased from Macherey-Nagel for column chromatography and LiChroprep® RP-18 (40-63 µm) from Merck for reversed-phase chromatography. The respective eluents were purchased in HPLC quality. For thin-layer chromatography, ALUGRAM® Xtra SIL G/UV254 ready-to-use DC films from Macherey-Nagel and ALUGRAM® RP-18W/UV254 ready-to-use DC films from Macherey-Nagel were used. UV-active compounds were visualized at a wavelength of 254 nm using a Macherey-Nagel type 60 fluorescent indicator. Fluorescent compounds

were visualized with the above-mentioned fluorescence indicator at a wavelength of 366 nm. Self-prepared coloring reagents such as potassium permanganate, bromocresol green, vanillin and ninhydrin were used for UV-inactive compounds.

Nuclear magnetic resonance spectroscopy

^{1}H and ^{13}C NMR spectra were recorded at room temperature using the DPX 400 (Bruker), AMX 400 (Bruker), Ascend 400 Avance III HD (Bruker), DRX 500 (Bruker) or Ascend 600 (Bruker) devices. For the ^{1}H NMR spectra, calibration was performed using the residual proton signal of the respective solvent used [δ($CDCl_3$) = 7.26 ppm, δ(CD_3OD) = 3.31 ppm, δ(DMSO-d6) = 2.50 ppm]. The chemical shift δ is given in ppm and the coupling constant J in Hz. The following abbreviations or combinations of these were used for the multiplicities of the signals: s = singlet, d = doublet, t = triplet, q = quartet, m = multiplet, b = broad signal. The ^{13}C NMR spectra were calibrated using the chemical shift of the respective solvent used [δ($CDCl_3$) = 77.16 ppm, δ(CD_3OD) = 49.00 ppm, δ(DMSO-d6) = 39.53 ppm]. The chemical shift is given in ppm. The NMR spectra were analyzed using TopSpin 4.0.8 from Bruker.

High-resolution mass spectrometry

High-resolution mass spectrometry was carried out on the following devices:

- HR UPLC-MS: Waters QTof Premier (ESI- and APCI-MS/MS) with UPLC system (Waters Acquity™ incl. TUV UV-detector)

- HR HPLC-MS: Micromass LCT Premier with HPLC system (Waters Alliance 2695)

- UPLC-MS: Xevo™ QTof MS (Waters Zspray™, ESI) with UPLC system, (Waters Acquity™, incl. photodiode array detector)

The calculated and detected masses are shown.

Aldehyde synthesis

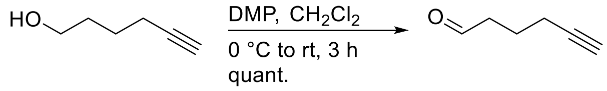

The Dess-Martin-periodinane reagent (2.16 g, 5.10 mmol, 1.00 eq.) in dichloromethane (27.6 mL) was cooled to 0 °C. 5-Hexin-1-ol (0.50 g, 5.10 mmol, 1.00 eq.) was added dropwise and the reaction mixture was warmed up to room temperature after 5 minutes. After 18 hours, the reaction mixture was filtered through a pad of Celite™ (ethyl acetate) and the solvent was removed under reduced pressure. After purification by column chromatography (pentane:diethyl ether 6:1), the aldehyde (0.48 g, 5.00 mmol, 98 %) was obtained as a colorless oil and directly employed in the next step.

Rf = 0.28 (pentane:diethyl ether 10:1);

ESI-HRMS: m/z calculated for C6H8ONa [M+Na]+: 119.1188, found: 119.1197.

Ester synthesis

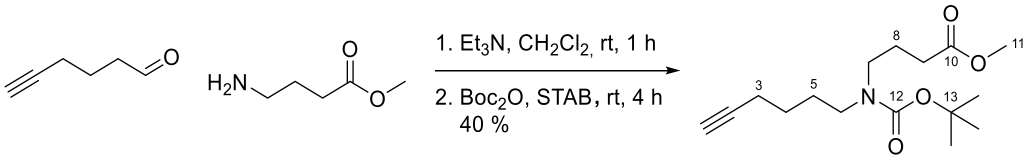

4-Aminobutyric acid methyl ester (585 mg, 5.00 mmol, 1.00 eq.) and triethylamine (1.7 mL, 12.50 mmol, 2.50 eq.) were added to a solution of the aldehyde from above (0.48 g, 5.00 mmol, 1.00 eq.) dissolved in dichloromethane (50 mL). The reaction mixture was stirred for 2 hours before di-tert-butyl dicarbonate (1.28 mL, 6.00 mmol, 1.20 eq.) and sodium triacetoxy-borohydride (STAB) (2.65 g, 12.50 mmol, 2.50 eq.) were added. After stirring for 18 hours at rt, the reaction was terminated by adding a saturated sodium hydrogen carbonate solution (50 mL). After phase separation, the aqueous phase was extracted with dichloromethane (3 x 50 mL). The combined organic phases were dried over Na2SO4, filtered and the solvent removed under reduced pressure. After purification by column chromatography (petroleum ether:ethyl acetate 10:1), the desired ester (668 mg, 2.25 mmol, 45 %) was obtained as a colorless oil.

$\boldsymbol{R}_f$ = 0.33 (Petroleum ether/ethyl acetate 6:1);

^{1}H-NMR (400 MHz, $CDCl_3$) δ = 3.67 (s, 3H, 11-*H*), 3.19-3.16 (m, 4H, 6-*H*, 7-*H*), 2.31 (t, *J* = 7.39 Hz, 2H, 9-*H*), 2.23-2.19 (m, 2H, 3-*H*), 1.94 (s, 1H, 1-*H*), 1.87-1.80 (m 2H, 8-*H*), 1.66-1.59 (m, 2H, 5-*H*), 1.53-1.48 (m, 2H, 4-*H*), 1.44 (s, 9H, 14-*H*, 15-*H*, 16-*H*) ppm (see figure S1).

^{13}C-NMR (100 MHz, $CDCl_3$) δ = 173.8 (C10), 155.7 (C12), 84.3 (C2), 79.5 (C13), 68.7 (C1), 51.7 (C11), 46.6 (C7), 46.3 (C6), 31.4 (C9), 28.6 (C14, C15, C16), 27.6 (C5), 25.80 (C4), 23.9 (C8) 18.3 (C3) ppm (see figure S2).

ESI-HRMS: *m/z* calculated for $C_{16}H_{27}NO_4$ $[M+Na]^+$: 320.1838, found: 320.1831.

Fragment A synthesis

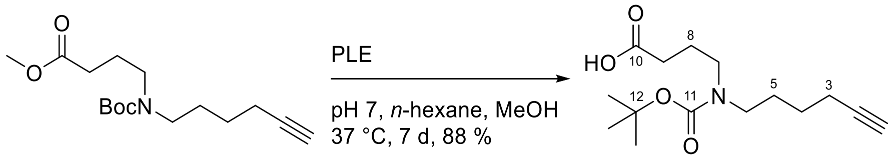

The ester from above (300 mg, 1.01 mmol, 1.00 eq.) was placed in hexane (5.05 mL) and methanol (0.51 mL) before a phosphate buffer solution (pH = 7.4, 50.5 mL) and PLE (48 mg, 720 U) was added. The reaction mixture was heated to 37 °C and cooled to room temperature after seven days. The pH was adjusted to 6 by adding hydrochloric acid (6 M) and the reaction mixture was extracted with ethyl acetate (3 x 60 mL). The combined organic phases were washed with an aqueous sodium hydrogen carbonate solution (5 %, 3 x 200 mL), dried over Na2SO4, filtered and the solvent removed under reduced pressure. Fragment A (252 mg, 0.89 mmol, 88 %) was obtained as a colorless oil.

R_f = 0.64 (Petroleum ether/ethyl acetate 1:1);

^{1}H-NMR (400 MHz, $CDCl_3$) *δ* = 3.26-3.18 (m, 4H, 6-*H*, 7-*H*), 2.35 (t, *J* = 7.30 Hz, 2H, 9-*H*), 2.23-2.20 (m, 2H, 3-*H*), 1.95 (s, 1H, 1-*H*), 1.86-1.84 (m, 2H, 8-*H*) 1.66-1.61 (m, 2H, 5-*H*) 1.54–1.48 (m, 2H, 4-*H*), 1.45 (s, 9H, 13-*H*, 14-*H*, 15-*H*) ppm (see figure S3).

^{13}C-NMR (100 MHz, $CDCl_3$) *δ* = 178.7 (C10), 156.0 (C11), 53.7 (C2), 51.9 (C12), 51.4 (C1), 33.9 (C7), 33.7 (C6), 31.4 (C9), 28.8 (C5), 28.7 (C13, C14, C15), 26.4 (C4), 24.4 (C8), 24.0 (C3) ppm (see figure S4).

ESI-HRMS: *m/z* calculated for $C_{15}H_{24}NO_4$ [M-H]: 282.1705, found: 282.1701.

Fragment B synthesis

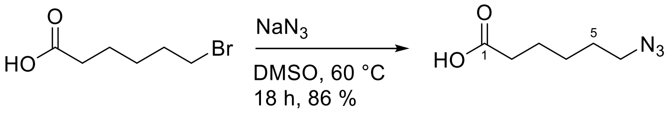

6-Bromohexanoic acid (1.00 g, 5.13 mmol, 1.0 eq.) was dissolved in DMSO (10.3 mL) at room temperature. Sodium azide (1.67 g, 25.63 mmol, 5.0 eq.) was added slowly and the reaction mixture was heated to 60 °C. After 18 hours, the reaction was terminated by adding dist. water (10 mL). After addition of dichloromethane (10 mL), the phases were separated and the aqueous phase was extracted with dichloromethane (3 x 15 mL). The combined organic phases were dried over Na_2SO4, filtered and the solvent removed under reduced pressure. After purification by column chromatography (CH2Cl2:MeOH 20:1), fragment B (0.66 g, 4.20 mmol, 82 %) was obtained as a colorless oil.

***R*f** = 0.50 (dichloromethane :methanol 6:1);

^{1}H-NMR (400 MHz, $CDCl_3$) δ = 11.37 (bs, 1H, 1-O*H*), 3.27 (t, *J* = 6.84 Hz, 2H, 2-*H*), 2.36 (t, *J* = 7.40 Hz, 2H, 6-*H*), 1.64 (m, 4H, 4-*H*, 5-*H*), 1.42 (m, 2H, 3-*H*) ppm (see figure S5).

^{13}C-NMR (100 MHz, $CDCl_3$) δ = 180.2 (C1), 51.3 (C6), 33.9 (C2), 28.6 (C5), 26.2 (C4), 24.2 (C3) ppm (see figure S6).

ESI-HRMS: *m/z* calculated for $C_6H_{10}N_3O_2$ $[M+H]^+$: 156.0773, found: 156.0769.

LNA-Fragment A conjugate synthesis

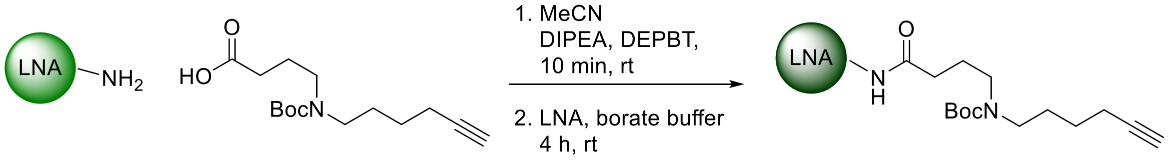

Fragment A (200 mM in acetonitrile, 5 µL), diisopropylethyl amine (DIPEA; 200 mM in acetonitrile, 5 µL) and 3- (diethoxyphosphoryloxy)-1,2,3-benzotriazine-4 (3H)-one (DEPBT; 200 mM in acetonitrile, 5 µL) were mixed in a thermal shaker for 10 minutes at room temperature. Borate buffer (0.5 M, pH = 9.5, 8 µL), LNA-21 (10 nmol in 5 µL H_2O) and dist. H_2O (5 µL) were added and the reaction mixture was left at room temperature for 4 hours without mixing. After that time the reaction was terminated by addition of ethanol (91 µL) and an aqueous sodium chloride solution (5 M, 2 µL) and the solution was incubated overnight at -20 °C. After centrifugation (14.8 rpm, 4 °C, 30 min), the supernatant was decanted, the pellet of the LNA-bearing Fragment A was taken up in ethanol (100 µL) and centrifuged again (14.8 rpm, 4 °C, 15 min). The decantation, take up in ethanol (100 µL) and centrifugation (14.8 rpm, 4 °C, 15 min) was repeated once more before the pellet was dried under vacuum.

SPION-Fragment B conjugate synthesis

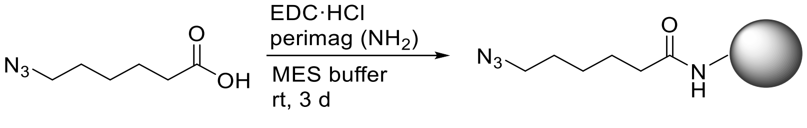

Fragment B (1.2 mg, 7.64 µmol) and (3-dimethylamino-propyl)-ethyl-carbodiimide hydrochloride (EDC-HCl; 1.5 mg, 7.82 µmol) were placed in aqueous MES buffer (0.5 M, 0.3 mL) and mixed in a thermal shaker for 10 minutes at 50 °C. Perimag®-NH_2 (10 mg iron/mL, 1 mL) was added and the reaction solution was mixed in a thermal shaker

for three days at room temperature. The solution was then transferred to a dialysis tube and dialyzed against deionized water for five days. The water was changed three times a day. After completion of dialysis, SPION-Fragment B conjugate (10 mg iron/5.5 mL H_2O) was transferred to Sarstedt tubes and stored at 4 °C.

SPION-LNA -21 conjugate synthesis

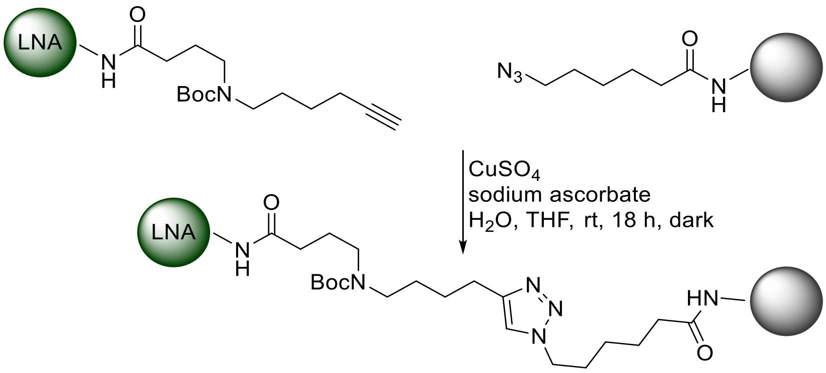

LNA conjugate (25 nmol) was dissolved in H_2O (75 µL) and added to the SPION bearing fragment (10 mg iron/5.5 mL H_2O). THF (2 µL), $CuSO_4$ (cat.) and sodium ascorbate (cat.) were added successively before the reaction mixture was shaken for 18 hours in the absence of light. The solution was then transferred to a dialysis tube and dialyzed against deionized water for four days. The water was exchanged three times a day. After completion of dialysis, SPION-LNA conjugate (10 mg iron/7.5 mL H_2O) was transferred to Sarstedt tubes and stored at 4 °C.

FITC-C6 synthesis

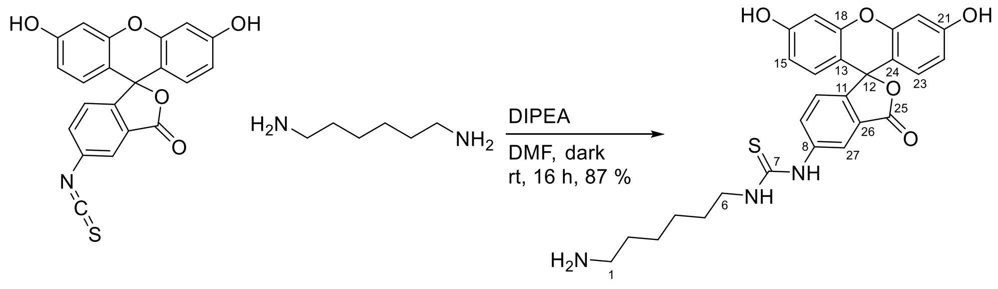

1.6-Diaminohexane (126 µL, 0.96 mmol, 5.00 eq.) was dissolved in dimethylformamide (4.82 mL). N,N-Di*iso*propylethylamine (328 µL, 1.93 mmol, 10.00 eq.) and a solution of fluorescein isothiocyanate (75 mg, 0.19 mmol, 1.00 eq.) in dimethylformamide (1.93 mL) was added slowly. The reaction mixture was stirred for 16 hours at room temperature in the absence of light. The reaction was terminated by removal of the solvent under reduced pressure. FITC-C6 (97 mg, 0.19 mmol, 87 %) was obtained as a reddish oil.

***R*f** = 0.20 (Reversed phase chromatography, dist. water:methanol 3:7);

^{1}H-NMR (400 MHz, CD_3OD) *δ* =8.50-8.44 (s, 1H, 27-*H*), 8.09-8.05 (s, 1H, 9-*H*), 7.20-7.16 (d, *J* = 6.71 Hz, 1H, 10-*H*), 6.84-6.78 (m, 1H, N*H*), 6.71-6.66 (d, *J* = 2.63 Hz, 2H, 14-*H*, 23-*H*), 6.60-6.56 (dd, *J* = 9.51, 2.96 Hz, 15-*H*, 17-*H*, 20-*H*, 22-*H*), 3.69-3.61 (bs, 2H, 6-*H*), 2.66-2.65 (s, 2H, 1-*H*), 1.76-1.65 (s, 2H, N*H*$_2$), 1.53-1.42 (m, 4H, 2-*H*, 5-*H*), 1.39-1.25 (m, 4H, 3-*H*, 4-*H*) ppm (see figure S7).

^{13}C-NMR (100 MHz, CD_3OD) *δ* = 182.7 (C7), 179.3 (C25), 176.0 (C16, C21),171.7 (C18, C19), 169.4 (C11), 155.3 (C8), 151.6 (C8), 143.1 (C14, C23), 142.0 (C10), 130.4 (C26), 121.1 (C27), 115.5 (C15, C22), 112.6 (C13, C24), 103.6 (C17, C20), 103.4 (C12), 45.8 (C6), 40.7 (C1), 36.5 (C2), 33.1 (C5), 29.7 (C4), 23.7 (C3) ppm (see figure S8).

ESI-HRMS: *m/z* calculated for $C_{27}H_{28}N_3O_5S$ $[M+H]^+$: 506.5968, found: 506.5969.

Test system synthesis (with fluorescein-label)

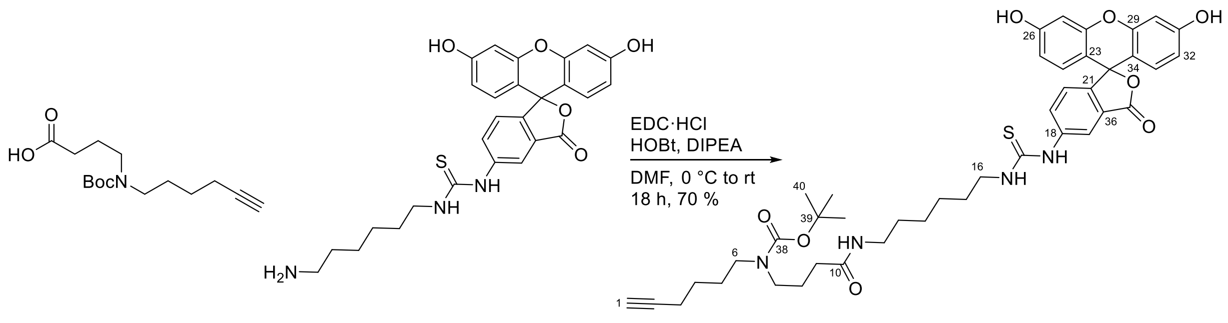

FITC-C6 (30 mg, 0.06 mmol, 1.00 eq.) was dissolved in dimethylformamide (594 µL). Element A (252.3 mg, 0.89 mmol, 15.00 eq.) was placed in dimethylformamide (8.91 mL) and cooled to 0 °C. EDC-HCl (34 mg, 0.18 mmol, 1.50 eq.), HOBt-H_2O (19.3 mg, 0.14 mmol, 1.20 eq.) and *N,N*-diisopropylethylamine (60.6 µL, 0.36 mmol, 3.00 eq.) were added and the solution was stirred for 30 minutes before it was slowly dropped into the FITC-C6 solution. Stirring was continued at room temperature for 18 hours. Then, the solvent was removed under reduced pressure and test conjugate (46 mg, 0.06 mmol, 70 %) was obtained as a reddish oil.

***R*$_f$** = 0.40 (Reversed phase chromatography, dist. water:methanol 3:7);

^{1}H-NMR (400 MHz, $(CD_3)_2SO$) *δ* = 8.50-8.49 (s, 1H,37-*H*), 8.30-8.20 (s, 1H, N*H*), 7.80-7.71 (s, 1H, 19-*H*), 7.18-7.09 (d, *J* = 2.92 Hz, 20-*H*), 6.68-6.65 (m, 1H, N*H*), 6.65-6.60 (m, 2H, 24-*H*, 33-*H*), 6.60-6.54 (m, 2H, 27-*H*, 30-*H*), 6.52-6.45 (m, 2H, 25-*H*, 32-*H*), 3.53-3.42 (m, 2H, 16-*H*), 2.79-2.58 (m, 7H, 1-*H*, 6-*H*, 7-*H*, 11-*H*), 2.42-2.36 (s, 4H, 3-*H*; 9-*H*), 2.04-1.95 (m, 2H, 8-*H*), 1.65-1.40 (m, 6H, 4-*H*, 5-*H*, 15-*H*), 1.38-1.28 (bs, 9H, 40-*H*, 41-*H*, 42-*H*), 1.28-1.20 (bs, 6H, 12-*H*, 13-*H*, 14-*H*) ppm (see figure S9).

^{13}C-NMR (100 MHz, $(CD_3)_2SO$) *δ* = 180.2 (C17), 168.9 (C10), 166.3 (C35), 153.4 (C26, C31, C38), 141.3 (C28, C29), 129.5 (C21), 110.5 (C18), 102.3 (C19), 78.8 (C24, C33), 43.6 (C36, C20), 35.1 (C37), 31.3 (C25, C32), 29.1 (C23, C34), 28.9 (C27, C30), 28.7

(C22), 28.6 (C2), 28.0 (C12, C15), 27.9 (C39, C1), 26.6 (C6, C7), 26.2 (C16), 26.1 (C40, C41, C42), 25.7 (C11), 25.6 (C4, C5, C13, C14), 25.1 (C9), 22.1 (C8), 14.0 (C3) ppm (see figure S10).

ESI-HRMS: *m/z* calculated for $C_{42}H_{49}N_4O_8S$ $[M+H]^+$: 769.3271, found: 769.3271.

Quantification of SPION conjugate

The native perimag® particles carry an unmodified dextran layer(OH). The perimag®-NH2 used in this work were subsequently functionalized by the manufacturer and contained 5-10 nmol amino groups per 1 mg iron/mL.In order to determine the exact degree of functionalization of these commercial batches, an SPDP assay was performed. Perimag®-NH_2 (10 mg iron/mL) was stirred in the presence of a phosphate buffer (pH = 7.4, 3.02 mL) and SPDP (20 mM, 11 mg) at room temperature for 18 hours. The particles were then washed with phosphate buffer (3 x 1 mL) via a MACS® column on a QuadroMACS™ separator. 100 µL of the SPDP-modified solution was removed and DTT (50 mM, 771 µg) was added. Then the solution was stirred for 15 minutes at room temperature, the particles were centrifuged at constant temperature and at 14.8 rpm over a period of 15 minutes. The absorbance at 343 nm was measured from the supernatant. The difference between the absorbance of the supernatants of SPDP-modified particles and the perimag®-NH_2 was used to calculate the degree of functionalization.

The same method was used to quantify the degree of functionalization of SPION conjugate. Here, the sites to which the conjugate has not bound are reacted with SPDP, thus indirectly determining the degree of functionalization.

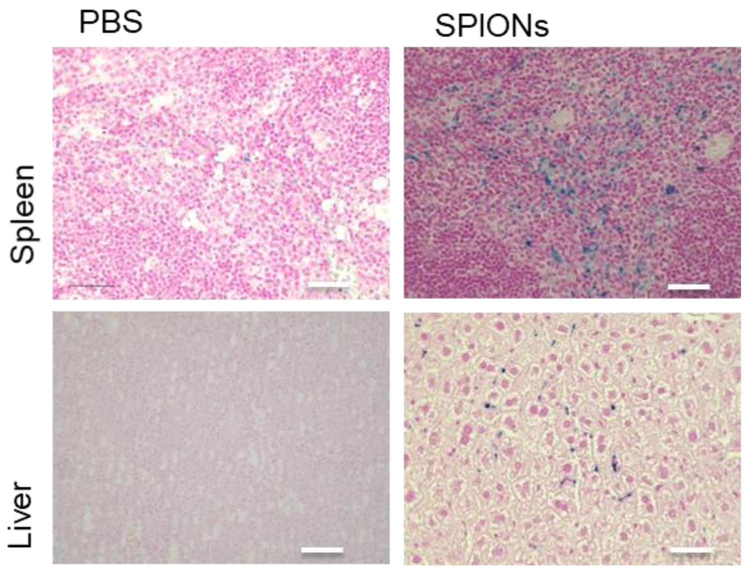

Figure S1: Biodistribution of SPIONs. Mice were intravenously injected with PBS or SPIONS (5 mg iron) and organs were harvested after 7 days. Microscopic images were taken of liver and spleen tissue that were stained with perl´s prussian blue. Representative images of n= 5-7 animals per group. Scale bar=50µm

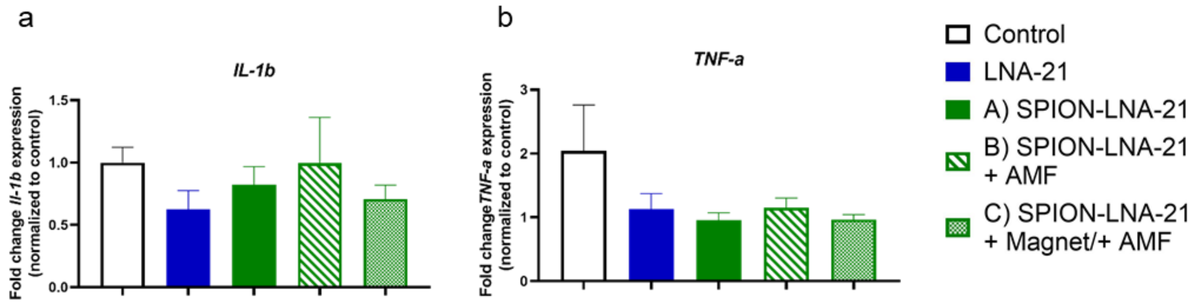

Figure S2: Evaluation of immune response. Mice were either intravenously injected with PBS (N=6), LNA-21 (2.5 mg/kg body weight, N=6) or SPION-LNA-21 conjugate (2.5 mg/kg body weigh, n=5) (group A) or injected with SPION-LNA-21 conjugate (2.5 mg/kg body weight, n=5) and an alternating magnetic field (AMF, 25 mT, 397 Hz) was

applied (group B) or injected with SPION-LNA-21 conjugate (2.5 mg/kg body weight, n=8) and a combination of an external magnetic belt on the heart during the injection and subsequent application of an AMF (group C) was used. Organs were harvested after two days and gene expression of inflammatory markers interleukin-1b (IL-1b) and tissue necrosis factor alpha (TNF-a) were measured in heart tissue**.** Data are presented as mean ±SEM; One-way ANOVA with Tukey´s multiple comparison test

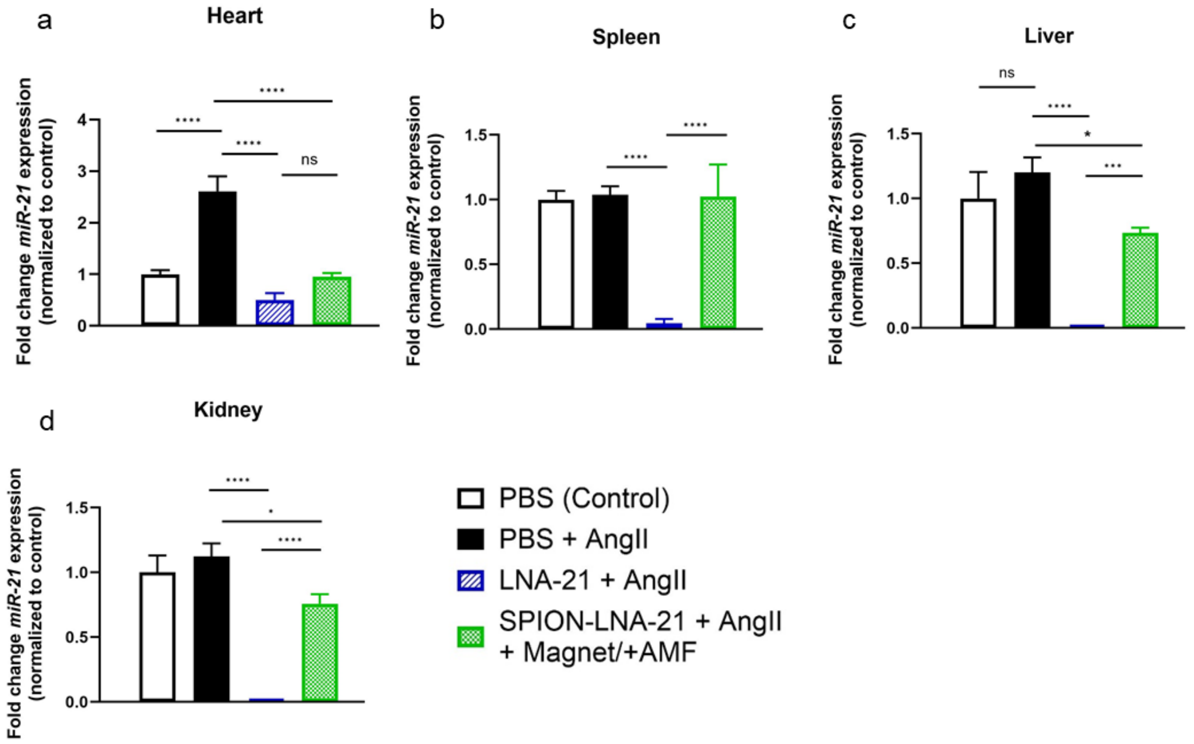

Figure S3: Evaluation of heart-specific delivery of LNA-21 in a therapeutic mouse model. On day 0 an osmotic pump was subcutaneously implanted that constantly releases angiotensin-II (3 mg/kg BW/day) over a time period of 14 days. Control mice were sham-operated. On day three and day ten mice were intravenously injected with PBS, LNA-21 (2.5 mg/kg body weight) or SPION-LNA-21 conjugate (2.5 mg/kg body weight) with a magnetic belt on the heart during the injection and subsequent application of an alternating magnetic field (AMF, 25 mT, 397 Hz) to release the LNA.

Organs were harvested after 14 days and miRNA-21 expression levels were measured in heart (**a**), spleen (**b**), liver (**c**) and kidney (**d**). Data are presented as mean ±SEM; one-way ANOVA with Tukey multiple comparison test; N= 6 in the PBS group, n=7 in the PBS+AngII group, n=9 in the LNA-21+AngII group and n=5 in the SPION-LNA-21 + AngII group

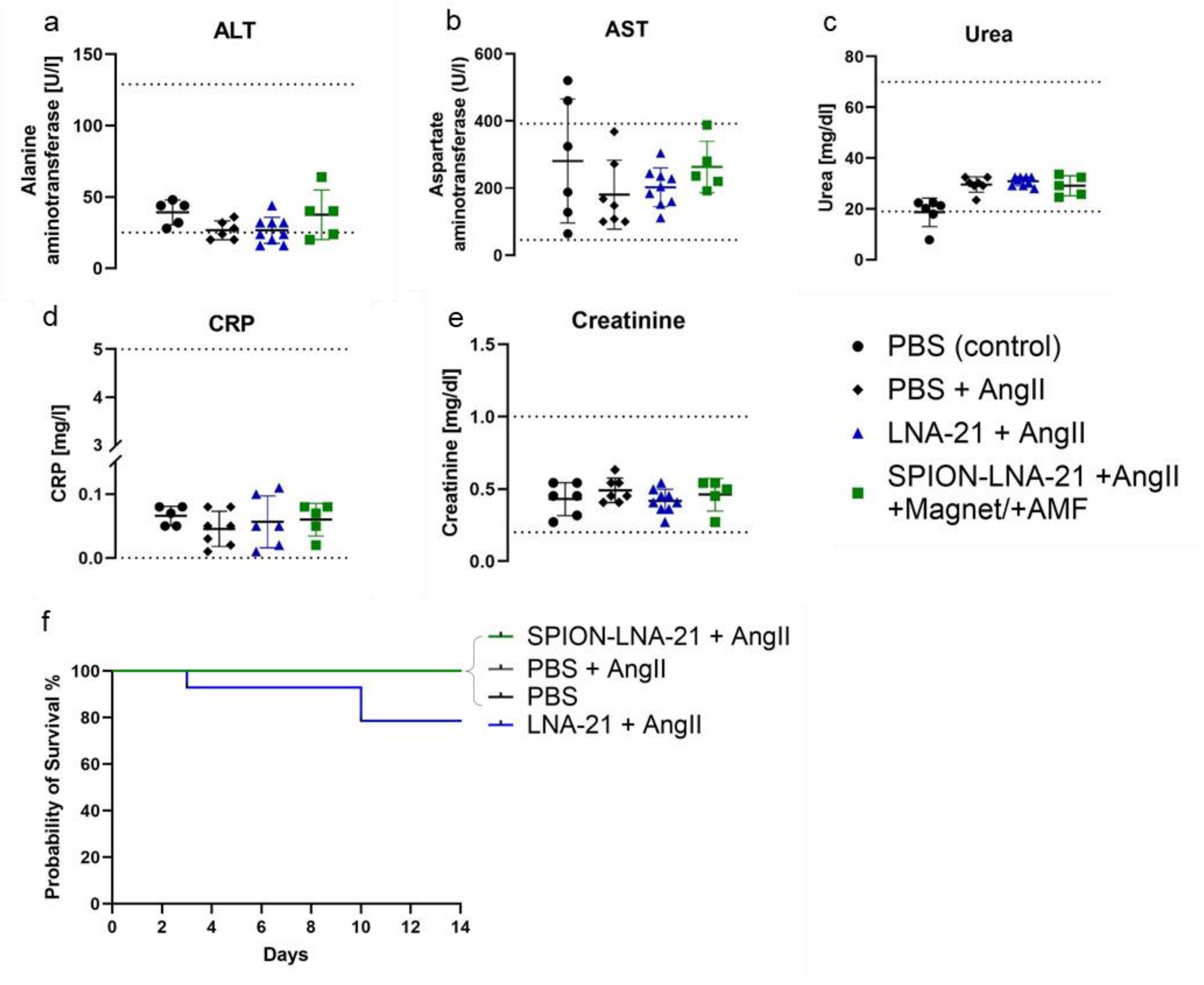

Figure S4: Analysis of plasma markers for renal and liver cytotoxicity. On day 0 an osmotic pump was subcutaneously implanted that constantly releases angiotensin-II (3 mg/kg/day) over a time period of 14 days. Control mice were sham-operated. On day three and day ten mice were intravenously injected with PBS, LNA-21 (2.5 mg/kg body weight) or SPION-LNA-21 conjugate (2.5 mg/kg body weight) with a magnetic belt on the heart during and post injection for 30 min and subsequent application of an

alternating magnetic field (AMF, 25 mT, 397 Hz) for 30 min to release the LNA. Mice were sacrificed after 14 days. Plasma concentrations of liver (ALT= Alanine aminotransferase (**a**), AST = Aspartate aminotransferase (**b**),) and kidney damage (creatinine (**c**) and urea (**d**)) as well as C-reactive protein (CRP; (**e**)) as a marker for inflammation were measured via ELISA. Data are presented as mean ±SEM; N= 5-9 animals per group. (**f**) The probability of survival was analyzed vie Kaplan-Meier-curve. N= 5-9 animals per group

NMR Spectra:

Ester

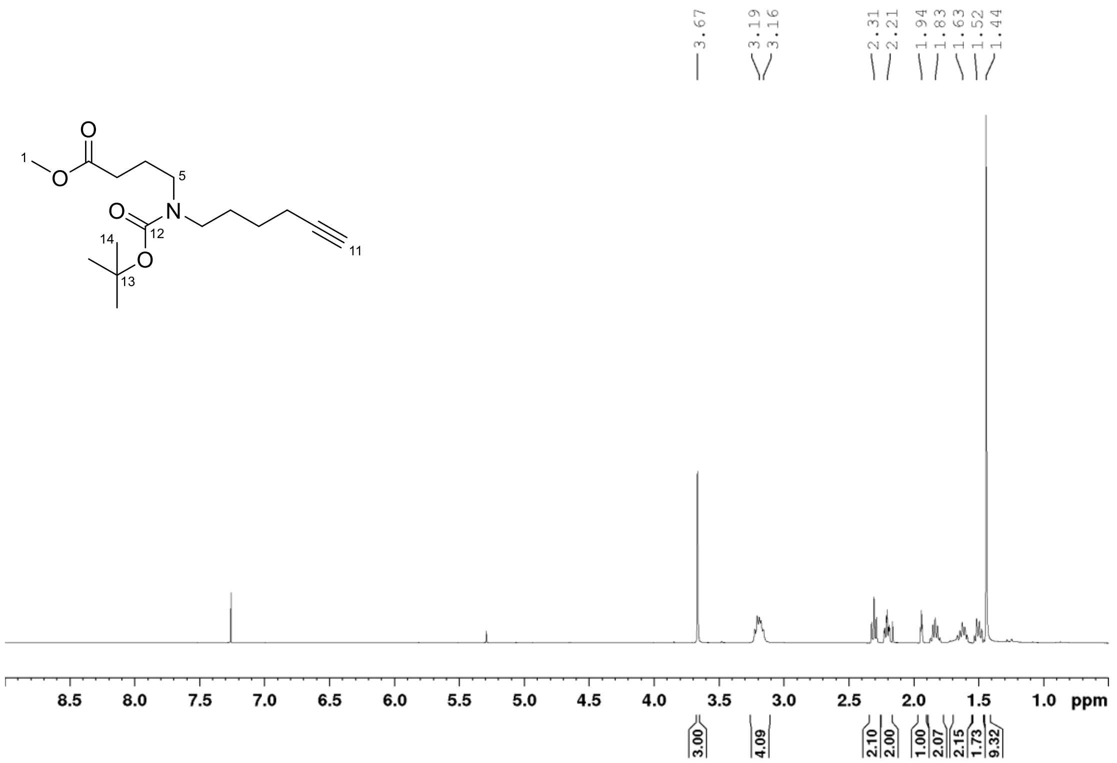

Figure S5: ^{1}H-NMR spectrum (400 MHz) of ester in $CDCl_3$.

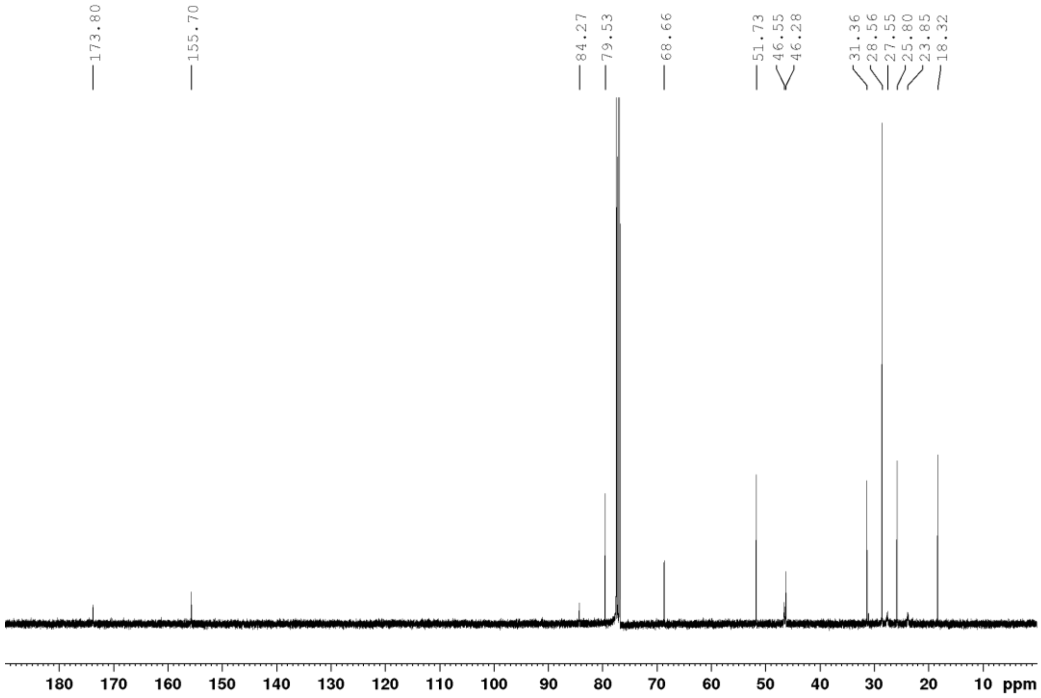

Figure S6: ^{13}C-NMR spectrum (100 MHz) of ester in $CDCl_3$.

Fragment A

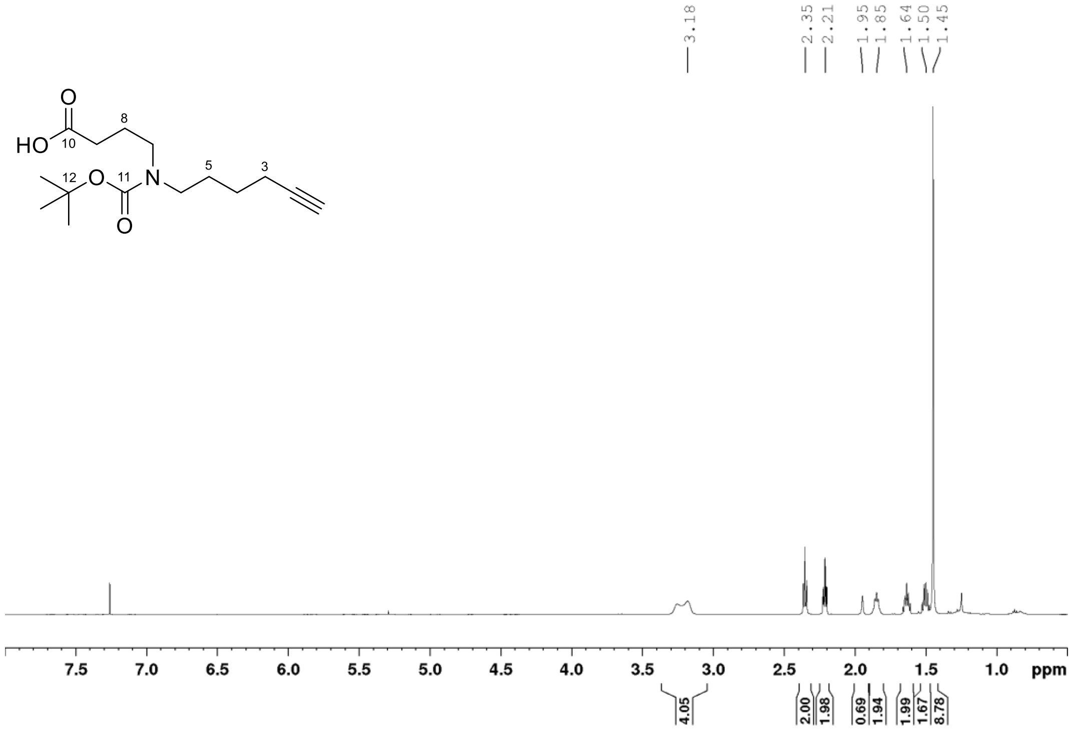

Figure S7: ^{1}H-NMR spectrum (400 MHz) of fragment A in $CDCl_3$.

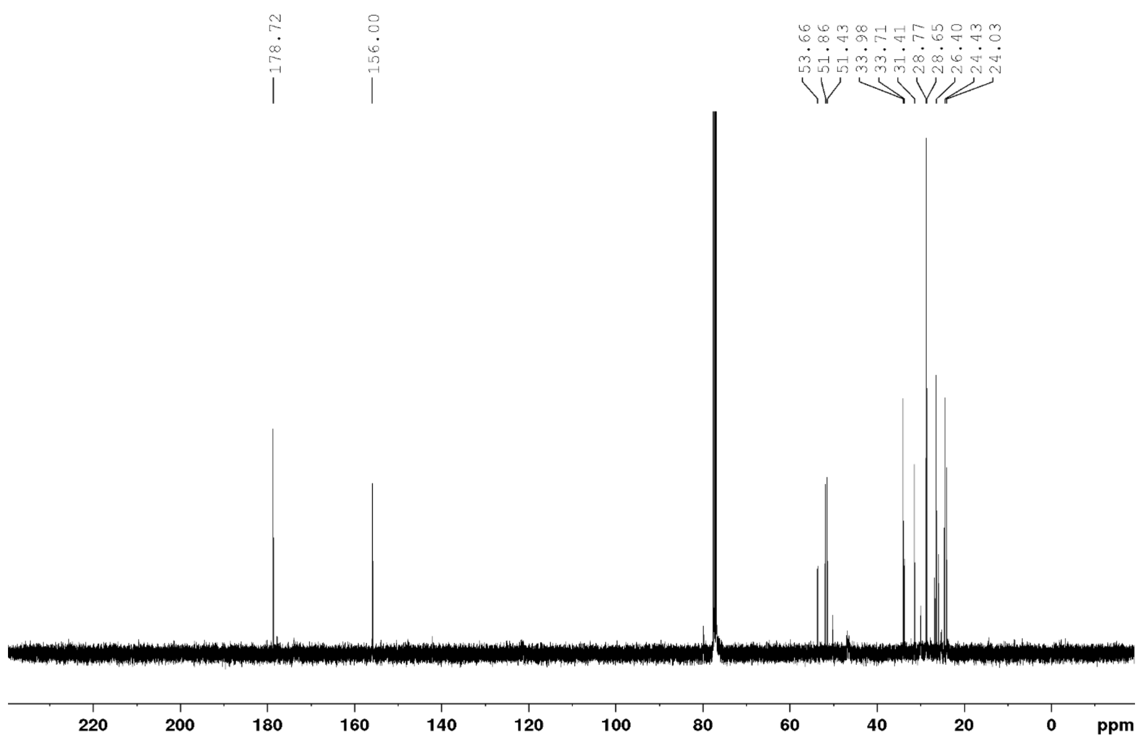

Figure S8: ^{13}C-NMR spectrum (100 MHz) of fragment A in $CDCl_3$.

Fragment B

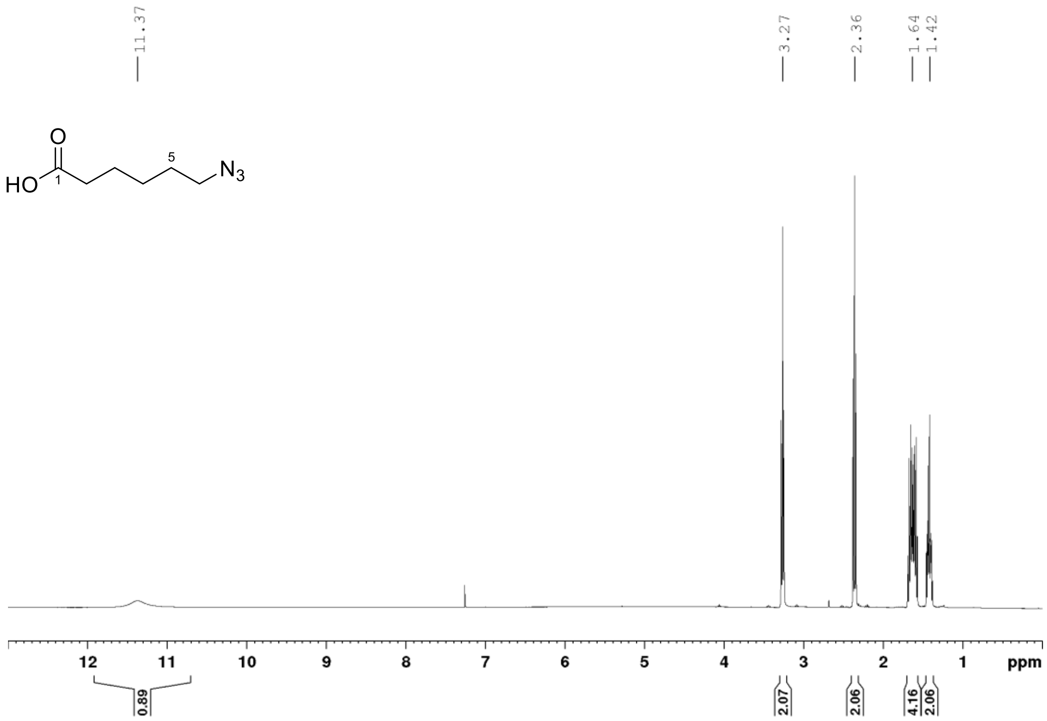

Figure S9: ^{1}H-NMR spectrum (400 MHz) of fragment B in $CDCl_3$.

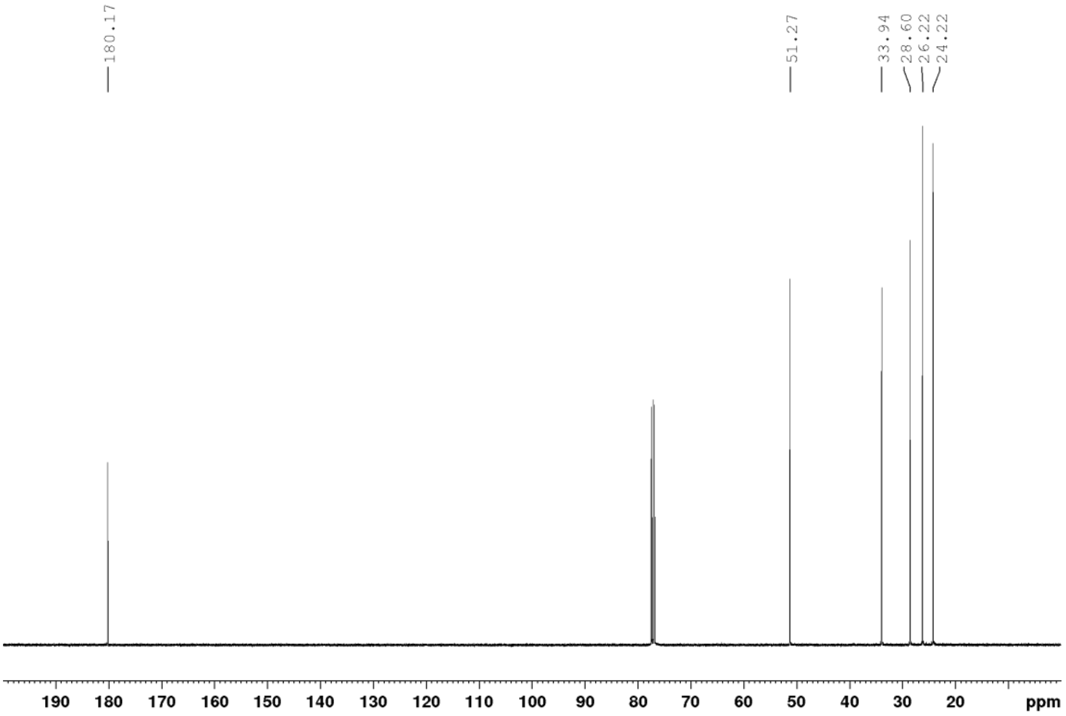

Figure S10: ^{1}H-NMR spectrum (100 MHz) of fragment B in $CDCl_3$.

FITC-C6

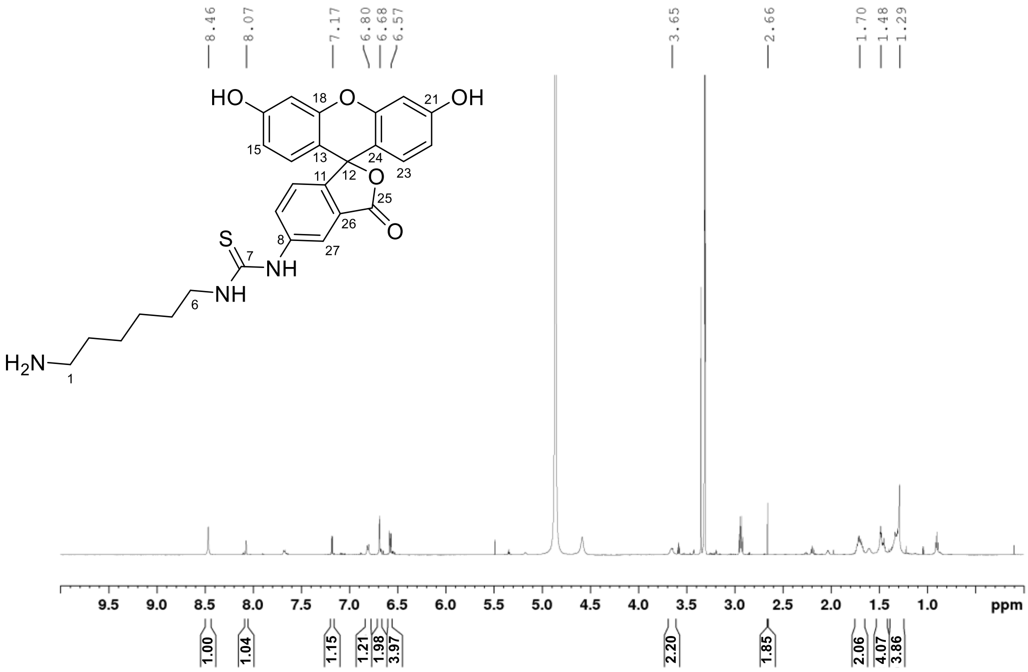

Figure S11: ^{1}H-NMR spectrum (400 MHz) of FITC-C6 in D_3COD.

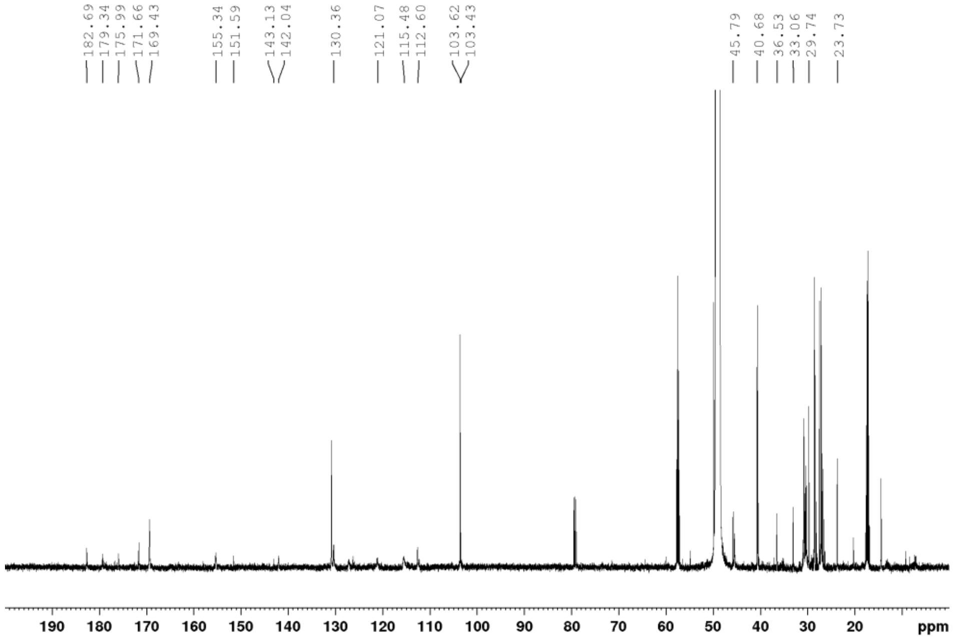

Figure S12: ^{13}C-NMR spectrum (100 MHz) of FITC-C6 in D_3COD.

Test substrate

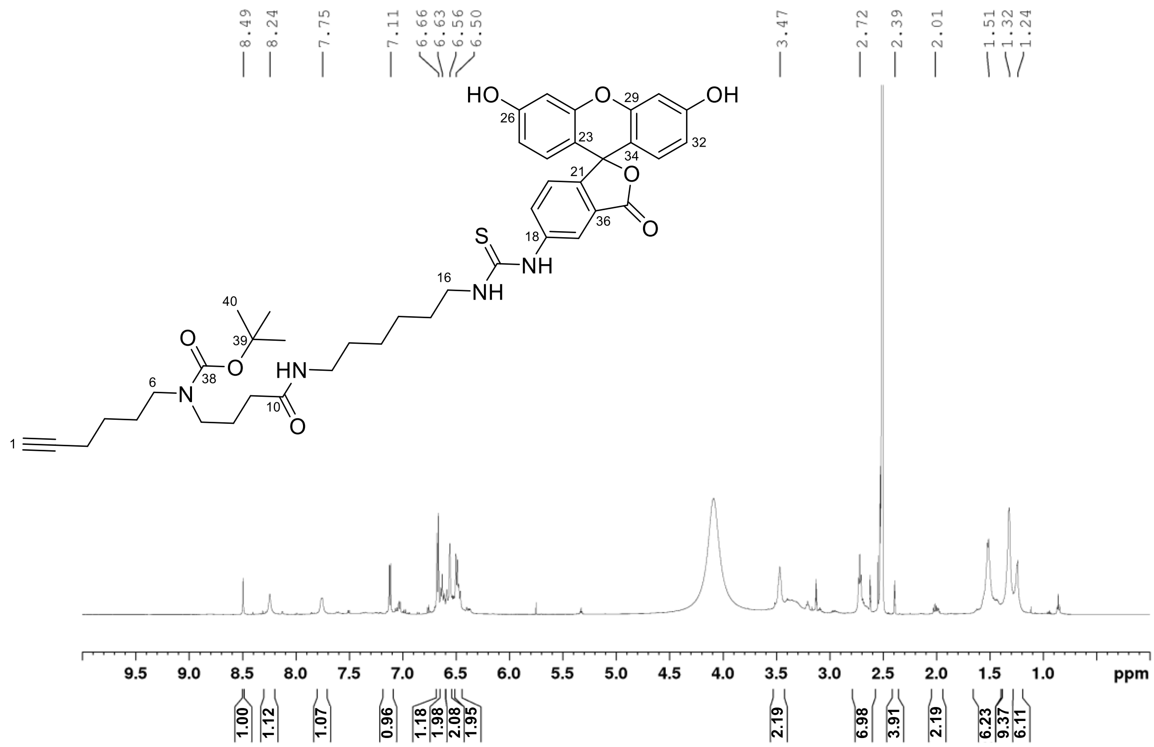

Figure S13: ^{1}H-NMR spectrum (400 MHz) of test substrate $(D_3C)_2SO$.

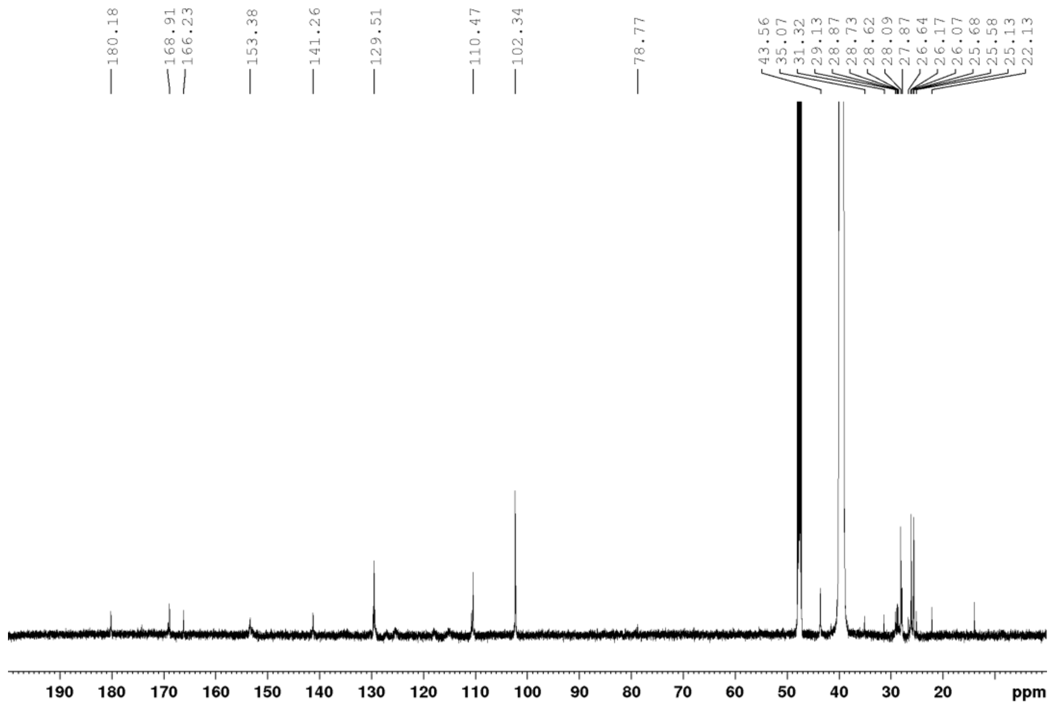

Figure S14: ^{13}C-NMR spectrum (100 MHz) of test substrate in $(D_3C)_2SO$.
