# Supplementary material for: Time- and spatially resolved LNA delivery via thermally controlled SPION technology
Source: Mol Ther Nucleic Acids. 2026 Mar 13;37(2):102902. doi: 10.1016/j.omtn.2026.102902 (PMC13068855; doi:10.1016/j.omtn.2026.102902)
Supplement: Document S2. Article plus supplemental information [file mmc2.pdf]

Molecular Therapy
Nucleic Acids
Original Article

Time- and spatially resolved LNA delivery via thermally controlled SPION technology

Franziska Kenneweg,[1,6] Katharina Hempel,[1,2,6] Lukas Philipp Joachim Höhne,[1,6] Gerald Dräger,[2] Jonas Blume,[1] Thilo Viereck,[3,4] Anastasia Stohwasser,[2] Sonja Groß,[1] Gwen Büchler,[1] Karina Jansen,[1] Malte Juchem,[1] Christian Bär,[1] Angelika Pfanne,[1] Annette Just,[1] Sabrina Thum,[1] Anika Gietz,[1] Andreas Kirschning,[2,5] and Thomas Thum[1]

[1]Institute of Molecular and Translational Therapeutic Strategies (IMTTS), Hannover Medical School, 30625 Hannover, Germany; [2]Institute of Organic Chemistry, Leibniz University Hannover, 30167 Hannover, Germany; [3]Institute for Electrical Measurement Science and Fundamental Electrical Engineering (emg), TU Braunschweig, 38106 Braunschweig, Germany; [4]Laboratory for Emerging Nanometrology (LENA), TU Braunschweig, 38106 Braunschweig, Germany; [5]Uppsala Biomedical Center (BMC), Uppsala University, 75237 Uppsala, Sweden

Targeted RNA delivery with precise spatial and temporal control marks a significant advancement in therapeutic development, offering the potential to reduce drug dosages while minimizing off-target effects. In this study, we present a novel platform that employs superparamagnetic iron oxide nanoparticles (SPIONs) for externally controlled, thermally triggered, organ-specific locked nucleic acid (LNA) release. Our platform technology leverages a newly designed thermosensitive conjugate, based on a thermosensitive linker system that utilizes the thermal sensitivity of the *tert*-butyloxycarbonyl (Boc) group. This tool ensures stability during systemic circulation while enabling traceless, on-demand drug release at the target site. As a proof of concept, we applied this technology in a disease model of cardiac fibrosis, conjugating SPIONs with an inhibitor of microRNA (miRNA)-21, a key pro-fibrotic regulator. The nanoparticle system was thoroughly characterized for its stability, biocompatibility, and heat-induced release properties *in vitro* and subsequently validated for biodistribution, toxicology, and therapeutic potential in preclinical *in vivo* models. This innovative SPION-based delivery platform provides a versatile and precise framework for RNA-based therapeutics, with broad translational potential across various disease applications.

INTRODUCTION

The development of RNA-based therapeutics has opened new frontiers in precision medicine,[1,2] yet their clinical translation continues to be limited by challenges in achieving targeted, efficient, and controllable delivery.[3] Issues such as off-target effects, low cellular uptake, and potential immune responses limit the effectiveness of currently, usually systemically applied RNA delivery platforms.[4–6] To address these challenges, we introduce an advanced transport system leveraging superparamagnetic iron oxide nanoparticles (SPIONs)[7,8] in combination with a thermosensitive linker system, which is itself bound to a drug,[9–12] enabling externally controlled, time- and space-resolved RNA delivery.

SPIONs exhibit distinct magnetic properties that enable precise manipulation, real-time tracking, and remote heat induction through externally applied magnetic fields.[13–16] By integrating a thermosensitive conjugate based on the heat sensitivity of the *tert*-butyloxycarbonyl group (Boc),[17–19] we have developed a delivery system that remains stable during systemic circulation but enables traceless, thermally triggered release of its therapeutic cargo at the target site. This technology provides an innovative solution for RNA therapeutics by overcoming critical delivery barriers and ensuring localized, controlled drug activation.

As a proof of concept, we conjugated an inhibitor of microRNA (miRNA)-21, demonstrating its effective use in modulating fibrotic pathways.[20–22] *In vitro* studies confirmed the system's stability, biocompatibility, and tunable release properties, followed by validation in preclinical models. However, this approach is not limited to cardiac applications—it represents a broadly adaptable delivery technology with potential across various therapeutic areas. By enabling precise spatial and temporal control over RNA-based therapeutics, SPION-based delivery systems pave the way for a new era of highly selective, minimally invasive treatments.

Received 5 June 2025; accepted 11 March 2026;
https://doi.org/10.1016/j.omtn.2026.102902.

[6]These authors contributed equally

Correspondence: Thomas Thum, Institute of Molecular and Translational Therapeutic Strategies (IMTTS), Hannover Medical School, 30625 Hannover, Germany.
E-mail: thum.thomas@mh-hannover.de

© 2026 The Author(s).
Published by Elsevier Inc. on behalf of The American Society of Gene and Cell Therapy.
This is an open access article under the CC BY license (http://creativecommons.org/licenses/by/4.0/).

RESULTS

Superparamagnetic nanoparticles are conjugated to LNA-21 via a thermolabile linker conjugate, enabling organ-specific delivery and controlled release

To achieve controlled and targeted RNA inhibition, we developed an innovative approach that covalently binds a well-defined miRNA inhibitor to SPIONs using a newly designed thermosensitive linker conjugate, which, based on the BoC group, allows the liberation of a secondary amine that induces release of the drug cyclization. The system has the advantage that the payload remains inactive during circulation but enables precise, on-demand release upon the application of an alternating magnetic field (AMF). The applied magnetic field induces hysteresis losses, resulting in localized heating of the SPION core and subsequent degradation of the Boc group[17,18] and hence cleavage of the linker, enabling traceless release of the inhibitor or other coupled drugs at the target site. For proof of concept, we used a phosphorothioate-modified miRNA inhibitor known as locked nucleic acid (LNA), targeting the well-characterized pro-fibrotic miRNA-21.[20]

For the synthesis of the proposed SPION-LNA conjugate, we first searched for different types of SPIONs that might fulfill the following requirements: a) biocompatibility, b) ability to convert magnetic energy into heat, and c) possibility of functionalization. The surface functionalization of SPIONs provides binding sites for LNA attachment, with higher degrees of functionalization resulting in increased LNA-to-SPION ratios. The most promising SPION candidates were then tested *in vitro* for cell toxicity. The cellular morphology of cardiac fibroblasts remained unaltered, and the metabolic activity, as a measure of cellular integrity, was unaffected after treatment with different doses of Perimag (micromod) SPIONs (Figures 1A and 1B), confirming their biocompatibility. Perimag particles consist of an Fe_3O_4 core coated with a modified, amino-functionalized dextran shell, making them suitable for conjugation with LNA-21 via the thermosensitive linker system. Details on amino-modified dextran shells in SPIONs have been reported by Grüttner et al.[23]

To enable thermally triggered release, we modified LNA-21 by introducing a C6 building block with a terminal NH_2 group at the 5′ end. Two different thermosensitive linker elements, named fragment A and fragment B, were synthesized separately to prevent chemoselectivity issues. These fragments were then individually conjugated to LNA-21 as well as to SPIONs before being fused using classical "click" chemistry (Figure 1C; synthesis is explained in detail in the supplemental methods section). Upon applying an AMF, localized heating triggers the traceless release of LNA-21 through thermal cleavage of the Boc protecting group, followed by intramolecular cyclization and lactam formation.

SPION-LNA-21 conjugate is non-immunogenic and non-toxic

In the next step, biocompatibility was assessed in cardiac fibroblasts and cardiomyocytes, two main cell types of the myocardium. Our results confirmed that all individual components of the conjugate, including both linker molecules, SPIONs, LNA-21, and the complete LNA-SPION conjugate, were non-toxic even at high concentrations. This was demonstrated by measuring LDH release (Figures 2A and 2B) and caspase activity (Figure 2E), both of which showed no significant increase. Importantly, exposure to an AMF and the resulting heating of the SPION core did not induce cellular damage (green bar). These findings were further validated in key off-target cells, specifically renal and liver cell types, which also exhibited no signs of cytotoxicity (Figures 2C and 2D).

To further evaluate the safety profile of our lead construct, we investigated its immunogenicity in preclinical studies. A key signaling pathway of proinflammatory cytokine expression is the nuclear factor kappa B (NF-κB) pathway. To assess whether our nanoparticle system triggered an immune response, we utilized a bi-directional NF-κB-responsive reporter.[24] Our findings provided strong evidence that the SPION-based delivery system is not only well tolerated but also non-immunogenic, as indicated by the absence of NF-κB activation (Figure 2F).

For *in vivo* applications, it is essential that the bulk temperature remains within a biosafe range, not exceeding 42°C. To determine thermal safety, we measured the temperature of the construct in PBS at a concentration mimicking physiological conditions in the bloodstream (5,000 nM SPION-LNA-21 conjugate). Our results showed that field strengths up to μ0H = 25 mT maintained the temperature within the biosafe range. However, exposure to a higher SPION concentration or an increased magnetic field strength led to excessive heating, posing a risk of organ damage (Figures 2G–2I). These findings establish a clear framework for safe application parameters, ensuring that our system remains both effective and non-harmful under physiological conditions.

LNA-21 can be efficiently released after application of an AMF

After validating the safety aspects, we next focused on examining the controlled release of LNA-21 from the SPIONs in more detail. Since direct detection of the released LNA by mass spectrometry was not feasible, we developed a test substrate designed to mimic the original system. This substrate included a C6 building block with a terminal amine and a Boc-protected secondary amine, serving as the "target breakpoint" analogous to the bound LNA (Figure 3A). To enable detection, the test substrate was synthesized with fluoresceine isothiocyanate (FITC). We demonstrated that thermally induced release was most efficient at 85°C, achieving a release efficiency of 81%, while the conjugate system remained stable under physiological conditions (Figure 3B). Importantly, this does not imply that the aqueous suspension of the conjugate is exposed to such elevated temperatures; rather, the localized heating in the immediate vicinity of the SPION core is sufficient to trigger linker activation, while the surrounding medium remains at physiologically safe levels. To further investigate release kinetics, we examined the original SPION-LNA-21 conjugate using magnetic particle spectroscopy (MPS) (Figure 3C), comparing magnetic heating with external heating through the medium. Our results showed that magnetic heating

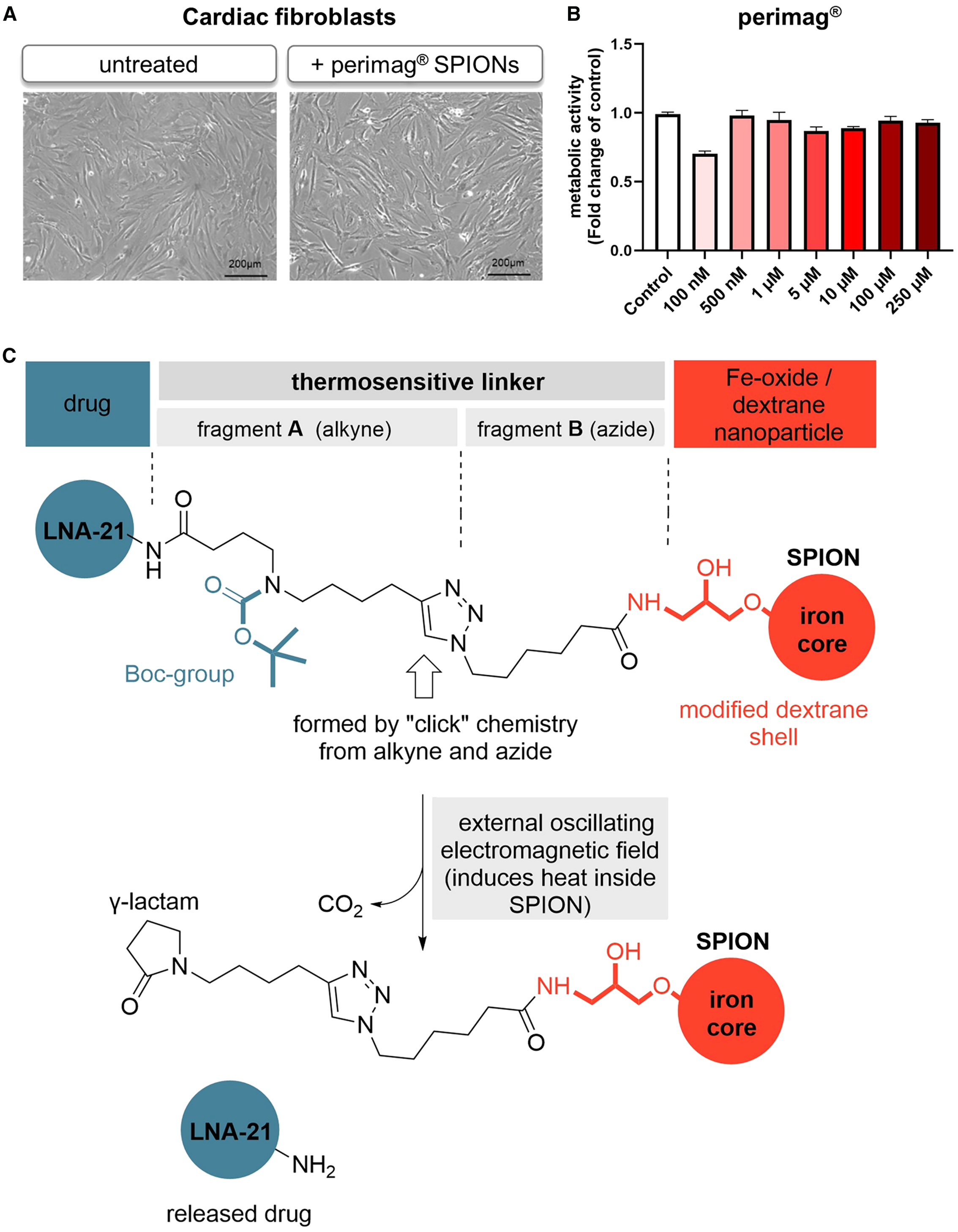

Figure 1. SPION-LNA-21 conjugate as organ-specific delivery system

(A) Microscopic bright-field images were taken after treatment of human cardiac fibroblasts with Perimag (250 μM) SPIONs, and cellular morphology was evaluated after 24 h. Representative images from *N* = 3 independent experiments are shown. Scale bars, 200 μm. (B) WST-1 assay was performed after treatment of human cardiac fibroblasts with different doses of Perimag (100 nM, 500 nM, 1 μM, 5 μM, 10 μM, 100 μM, and 250 μM). One-way-ANOVA test. Data are presented as mean ± SEM; *n* = 3 independent experiments (C) Molecular architecture of the LNA-21/linker/perimag (SPION) conjugate and the thermally induced mechanism of LNA-21 release.

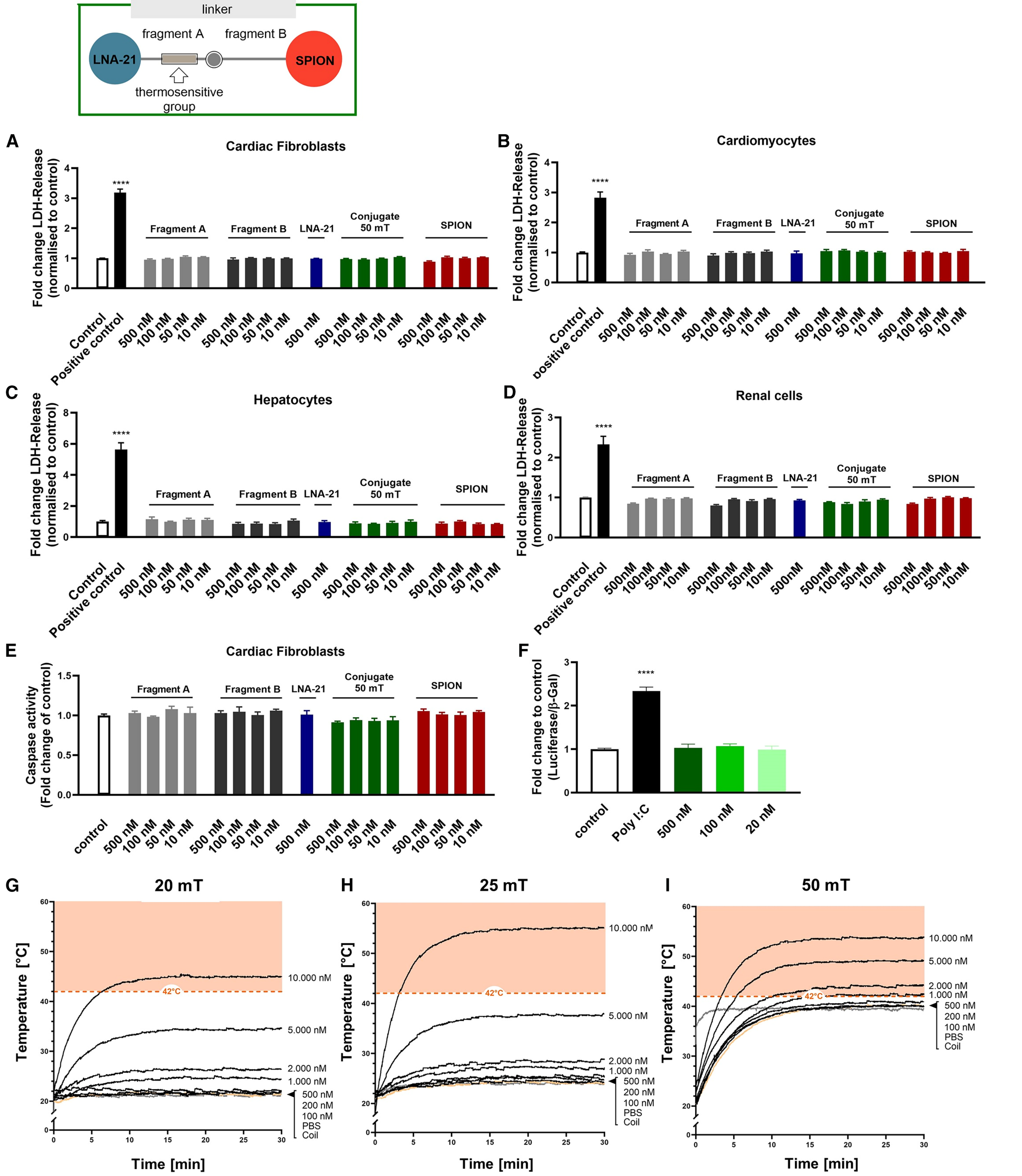

(legend on next page)

led to a significantly increased and accelerated LNA-21 release, suggesting that the magnetic SPION core undergoes localized overheating, thereby activating the thermo-linker more efficiently than uniform, external heating. To determine the specific field strength required for effective LNA-21 release, cardiac fibroblasts were transfected with varying doses of the SPION-LNA conjugate, followed by the application of an AMF at different field levels (Figure 3D), revealing that a minimum field strength of μ0H = 25 mT was required to successfully release LNA-21 and inhibit miR-21 expression. Additionally, we confirmed that the LNA remained inactive while covalently bound to the SPION. The release of LNA-21 at 25 mT was further validated by AC susceptibility (ACS) data (Figure 3E) and dynamic light scattering measurements (Figures 3F and 3G), which demonstrated a distinct change in (hydrodynamic) diameter before and after AMF exposure. These findings establish that controlled and localized heating via magnetic field application is a highly effective mechanism for precise, on-demand RNA delivery.

SPION-LNA-21 conjugate is safe in *in vivo* settings

After confirming the tolerability of the individual components of the nanoparticle system *in vitro*, we next investigated their behavior in human blood. To assess safety under physiological conditions, different doses of the SPION-LNA conjugate were mixed with human blood, followed by the application of an AMF for 30 min (Figure 4A). Consistent with our previous findings, a concentration of 5,000 nM of SPION-LNA-21 conjugate remained within the biosafe range, with key blood parameters, such as potassium and LDH levels, remaining stable and within normal limits (Figures 4B and 4C). These results further support the biocompatibility of the system, even under simulated *in vivo* conditions.

The first *in vivo* mouse study served as a proof-of-principle to evaluate the safety and tolerability of our nanoparticle construct at the highest feasible intravenous dose, limited by the lethal threshold of iron oxide in SPIONs.[13,14,16] To assess toxicity, mice were intravenously injected with the SPION-LNA-21 conjugate (5 mg iron), linker-free SPIONs (5 mg iron), LNA-21 at a dose previously published to be effective (20 mg/kg body weight[20,22]), or PBS as a control, and organs were harvested after 7 days (Figure 4D). While administration of pure LNA-21 resulted in an increased mortality rate, conjugation to SPIONs significantly reduced toxicity, indicating that even at the highest iron content, the nanoparticle construct was well tolerated (Figure 4F). Further assessment of kidney and liver function showed that key blood parameters, including AST (aspartate aminotransferase), ALT (alanine aminotransferase), creatinine, and urea levels, remained within the normal range (Figures 4I–4L). Additionally, cardiac gene expressions of inflammatory markers, such as *IL-6* (interleukin-6) and *TNF-α* (tissue necrosis factor-alpha) were not elevated (Figures 4G and 4H), further supporting the safety of the nanoparticle system.

To evaluate the biodistribution and functional impact of systemic administered LNA-21, *miR-21* expression levels were quantified in all major organs. The results demonstrated that systemic injection of LNA-21 led to widespread suppression of miR-21 across multiple organs, emphasizing the necessity for a more target-oriented delivery approach to enhance cardiac specificity (Figure 4E). As reported in the literature, antisense oligonucleotides like LNAs tend to accumulate primarily in the liver and kidneys,[25] with only a smaller fraction reaching the heart, posing a challenge for cardiac-targeted therapies. To investigate the biodistribution of our SPION-LNA conjugate, we performed Perl's Prussian blue staining on histological sections, confirming notable off-target accumulation, particularly in the liver and spleen (Figure S1). These findings confirm the safety of the SPION-LNA-21 construct while underscoring the importance of optimizing organ-specific RNA delivery.

SPION-LNA-21 conjugate enhances drug accumulation at the target site while reducing systemic off-target effects

Building on our previous findings, we next aimed to validate the efficient release of LNA-21 from the SPIONs *in vivo* and assess its effect on *miR-21* expression across different organs. To improve targeted delivery and evaluate release efficacy, we designed three experimental groups for the study: (A) intravenous injection of the SPION-LNA-21 construct without applying an AMF, ensuring that the LNA remained bound and inactive; (B) intravenous injection followed by AMF application to induce the release of LNA-21; and (C) a combination approach in which a magnetic belt was placed over the beating heart during injection and for an additional 30 min to attract the SPION-LNA-21 construct to the cardiac region, followed by AMF-triggered release (Figure 5A). Two days post-injection, organs were harvested, and *miR-21* expression was quantified as an indicator of successful LNA-21 release. Consistent with our previous findings, systemic injection of pure LNA-21 (blue column, Figures 5B–5E) resulted in widespread off-target effects across multiple organs, with *miR-21* expression in the heart reduced by only 50%. Notably, the combination of the external magnet and AMF (group C, green

Figure 2. Safety analysis of the SPION-LNA-21 system and its individual components

(A) Human cardiac fibroblasts, (B) neonatal mouse cardiomyocytes, (C) human liver cells (HepG2), and (D) rat kidney cells (NRK) were treated with different components of the conjugate system (SPIONs, Fragment A, Fragment B, SPION-LNA-21 conjugate + alternating magnetic field (AMF), 50 mT, 397 Hz) and LDH release and caspase 3/7 activity (E) were determined. For the negative control, cells were treated with PBS. For the positive control, cells were lysed 45 min before LDH release was measured. Data are presented as mean ± SEM; $n = 3$ independent experiments; one-way ANOVA with Tukey's multiple comparison test; (F) HEK293T cells were transfected with a bidirectional NF-κB-responsive reporter and treated with different concentrations of the SPION-LNA-21 conjugate, and luciferase activity was measured and normalized to β-Gal. For the positive control, cells were treated with 3 μg/mL poly I:C. $n = 3$ independent experiments. One-way ANOVA with Tukey's multiple comparison test;****$p \leq 0.0001$; ns = not significant. (G–I) The temperature profile of different doses of SPIONs in PBS was evaluated when different AMF field strengths (20, 25, and 50 mT, 397 Hz) were applied. Representative curves from independent experiments are shown.

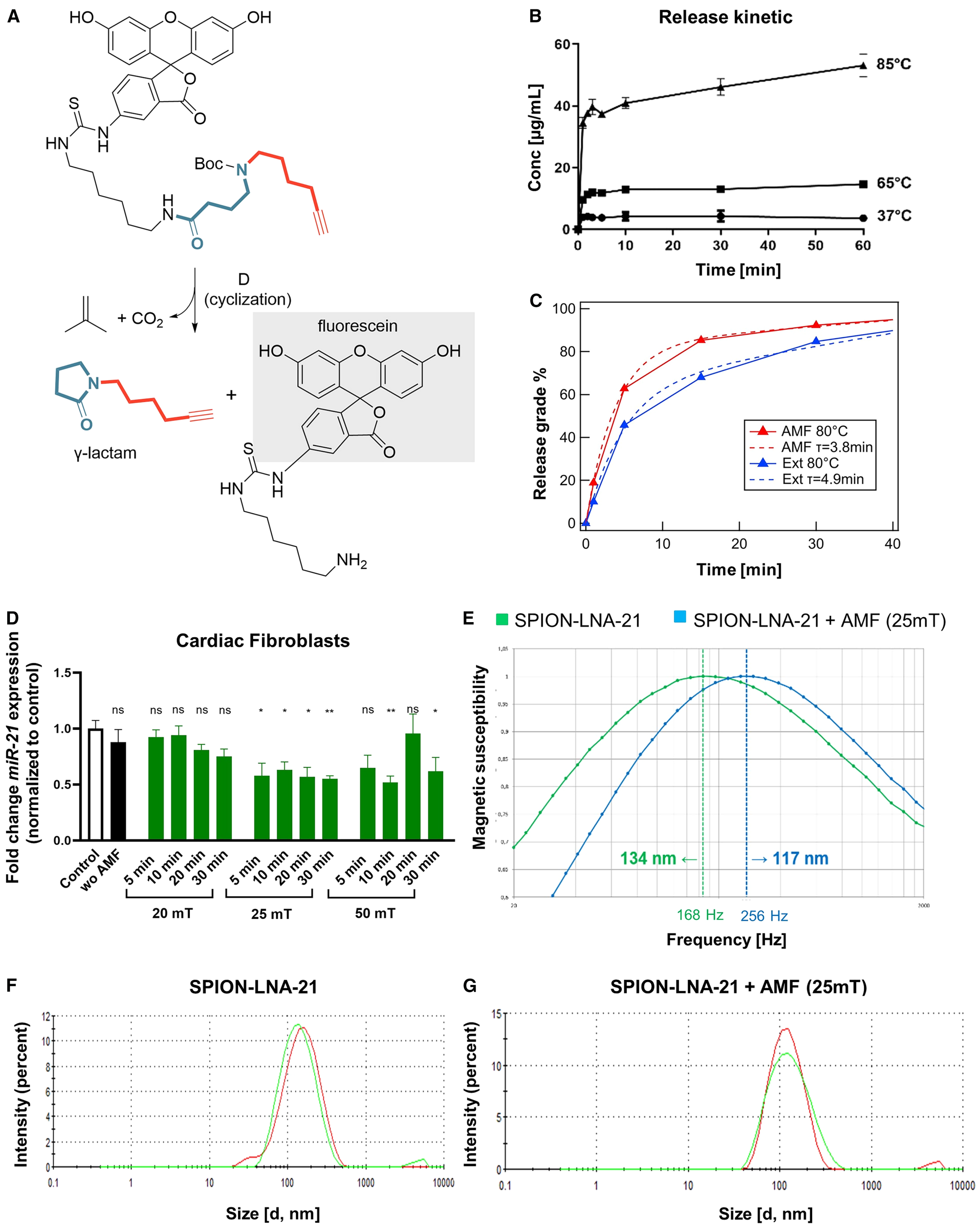

(legend on next page)

columns) significantly minimized off-target accumulation, particularly in the lungs, liver, and kidneys, while maintaining miR-21 suppression in the heart at levels comparable to pure LNA injection. These findings highlight the potential of magnetic field-assisted targeting as a promising strategy for cardiac-specific RNA delivery, while reducing systemic distribution and off-target effects.

To assess the biocompatibility of AMF for *in vivo* applications, we monitored survival rates and key blood parameters. All mice survived the procedure, and blood markers for kidney and liver function remained within the biosafe range (Figures 5F–5I), confirming that AMF exposure did not induce systemic toxicity. Interestingly, plasma IL-6 concentrations were slightly elevated following the injection of pure LNA-21. However, this effect was completely abolished when LNA-21 was conjugated to SPIONs and remained absent even after its release via AMF (Figure 5J). In line with these results, cardiac levels of inflammatory markers *Il1b* and *TNF-a* were not elevated after application of an AMF (Figures S2A and S2B).

SPION-LNA-21 conjugate efficiently reduced cardiac fibrosis and hypertrophy

To assess whether the significant reduction in cardiac *miR-21* levels achieved with the SPION-LNA-21 construct translated into therapeutic anti-fibrotic efficacy, we examined biochemical and functional improvements in a mouse model of early heart failure with reduced ejection fraction (HFrEF). The disease model was established by pharmacologically inducing hypertension through subcutaneous implantation of an osmotic pump, which continuously released angiotensin II (Ang II) over 14 days. This chronic stimulation of the renin-Ang-aldosterone system (RAAS) progressively led to myocardial hypertrophy and fibrosis, mimicking pathological cardiac remodeling. On the third and tenth days after pump implantation, mice were either treated with pure LNA-21 or with the SPION-LNA-21 conjugate in combination with AMF and a magnetic belt (Figure 6A).

After 14 days, organs were harvested to evaluate target engagement (Figures S3A–S3D). In the heart, both the SPION-LNA-21 construct and native LNA-21 significantly reduced *miR-21* expression to physiological levels. However, animals treated with pure LNA-21 exhibited a drastic reduction of *miR-21* in off-target organs, resembling a systemic miR-21 knockdown effect. In contrast, the magnetically targeted nanoparticle delivery system significantly reduced *miR-21* expression in the heart while limiting off-target effects. No significant reduction was observed in the spleen (Figure S3B), and only minor decreases were detected in the liver (Figure S3C) and kidneys (Figure S3D), likely due to the pharmacokinetic degradation of residual nanoparticles in these organs. Overall, intracellular *miR-21* expression in off-target organs was reduced by 60.7% in the liver, 67.1% in the kidney, and 98.8% in the spleen, while cardiac *miR-21* expression was completely normalized, demonstrating the efficacy of this magnetic drug delivery system in minimizing off-target effects. Echocardiographic assessment revealed a significant improvement in cardiac function (ejection fraction) following treatment with the SPION-LNA-21 conjugate (Figure 6B). Additionally, left ventricular hypertrophy was prevented only by the nanoparticle conjugate (Figure 6C).

To evaluate the therapeutic impact of this targeted approach, histological analysis of heart tissue revealed that treatment with the SPION-LNA-21 conjugate resulted in an even greater reduction in fibrosis, restoring it to physiological levels more effectively than native LNA-21 treatment (Figures 6D and 6E). This was supported by a significant reduction in fibrosis-associated genes, including *Col1a2, α-SMA*, and *MMP2* (Figures 6F–6H). Myocyte diameter measurements further confirmed that the SPION-LNA-21 conjugate significantly reduced cardiac hypertrophy, whereas native LNA-21 treatment had only a limited effect (Figures 6I and 6J). Consistently, hypertrophy markers *MCIP1.4, BNP*, and *ANP* were also significantly reduced in the SPION-LNA-21-treated group (Figures 6K–6M).

To rule out potential systemic toxicity, plasma markers of organ damage were analyzed, including ALT, AST, creatinine, and urea (Figures S4A–S4D), which remained within the normal range across all treatment groups. Immune response analysis revealed no elevation in C-reactive protein (CRP) levels in treated animals compared to controls or the reference range (Figure S4E), suggesting a low likelihood of chronic inflammation. Whereas all animals survived in the SPION-LNA-21 conjugate group, the probability of survival was reduced in the LNA-21 group (Figure S4F), underlining the safety of this nanoparticle system.

Our findings suggest that the key therapeutic advantage of the SPION platform lies in its ability to spatially control drug release while minimizing systemic exposure, rather than in achieving higher

Figure 3. Release kinetics of the SPION-LNA-21 conjugate

(A) Cyclization products of the test substrate FITC bound to the 4-(hex-5-yn-1-ylamino)butanoyl group and fragment A, analogous to the SPION-LNA-21 conjugate. After conventional heating, the products were detected by mass spectrometry, and the efficiency of the released fluorescein-bearing fragment (B) was measured at different temperatures (37°C, 65°C, and 85°C) $n = 3$ independent experiments. Data are presented as mean ± SEM. (C) The release grade of LNA-21 from the SPIONs was measured by magnetic particle spectroscopy, comparing magnetic heating (alternating magnetic field [AMF], red) and external heating (Ext, blue). $N = 5$ averages. (D) Human cardiac fibroblasts were treated with SPION-LNA-21 conjugate (200 nM) and subjected to different field strength (μ0H = 20, 25, 50 mT, 397 Hz, green) in an AMF to release the LNA. miRNA-21 expression levels were measured after 48 h via RT-qPCR. Data are presented as mean ± SEM, $n = 3$ independent experiments; one-way ANOVA with Dunnett's multiple comparison test; $*p \leq 0.05$; $**p \leq 0.01$. (E) The hydrodynamic diameter of the SPION-LNA-21 conjugate was estimated from the characteristic frequency (omega*tau = 1) in AC susceptibility measurements prior to heating (green) or after application of an AMF (μ0H = 25 mT, 397 Hz, blue) $N = 10$. (F and G) DLS (dynamic light scattering) measurements evaluated differences in hydrodynamic diameter without AMF and after application of an AMF (μ0H = 25 mT, 397 Hz). Representative images from $n = 3$ independent experiments are shown.

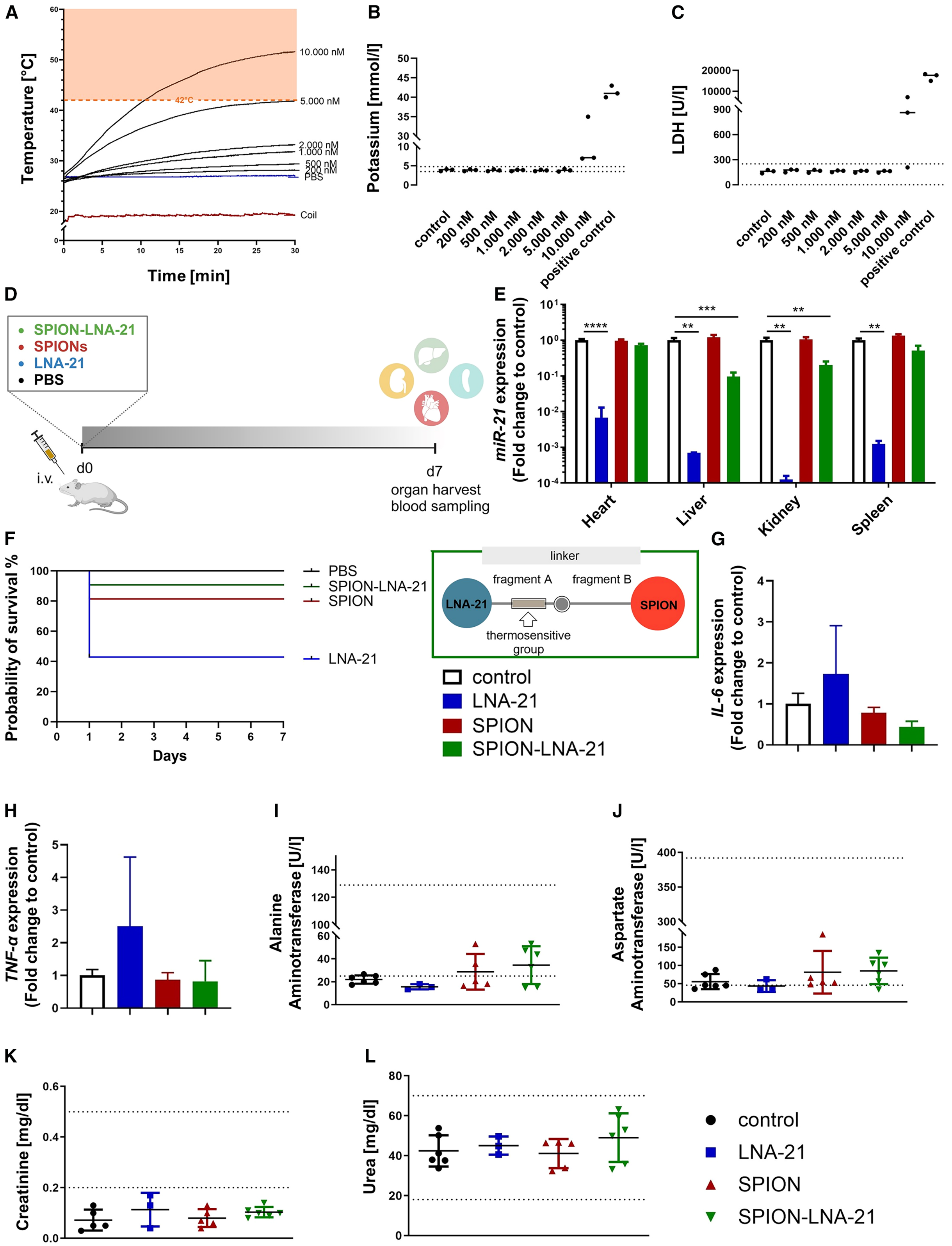

(legend on next page)

levels of cellular uptake in the target tissue. Moreover, this targeted approach significantly minimizes off-target effects while maintaining an excellent safety profile, highlighting its potential as a novel therapeutic strategy for cardiac fibrosis and other diseases requiring precise RNA-based interventions.

DISCUSSION

In this study, we successfully developed a novel, stable, and functional drug delivery system based on an SPION-linker-LNA conjugate. This system addresses key challenges that have hindered broader clinical translation of RNA therapeutics, including organ specificity, off-target effects, and immune responses. By coupling a therapeutic LNA of a well-characterized miRNA to the surface of SPIONs via a thermocleavable linker, we achieved active, externally controlled drug release. The application of an AMF induces localized heating, cleaving the linker and enabling on-demand release of LNA-21.

Previous studies have shown that pharmacological inhibition of miRNA-21 reduces fibrosis in multiple organs, such as the heart,[20,22,26] lung,[27] liver,[28] or kidney.[29–31] One key advantage of LNAs is their stability and ability to function without the need for transfection reagents, as demonstrated by Buntz et al.[32] However, systemic administration of miRNA-21 inhibitors is not organ-specific and, like most antisense oligonucleotides, predominantly accumulates in metabolic organs such as the liver and kidneys.[25] This highlights the urgent need for a targeted drug delivery system to overcome these limitations.

Here we demonstrated that AMF application efficiently triggers LNA release and that all components of the system, including the end products after cleavage, are well tolerated within therapeutic dose ranges in both *in vitro* and *in vivo* settings. Although the maximum permissible iron dose for *in vivo* applications remains a limiting factor, given the currently achieved quantity of bound RNA on the SPIONs, we observed no signs of iron-related toxicity at the dose levels used in this study.[13,14,16] Importantly, SPION-mediated localized heating remained below the biosafe threshold of 42°C at the maximum applicable iron concentration. This moderate temperature increase exhibited no cytotoxic effects on blood cells *in vitro*. Notably, while the SPION core reached localized temperatures of approximately 80°C to enable linker cleavage, efficient heat dissipation into the surrounding aqueous environment effectively mitigated thermal risk to adjacent tissues.

A key advantage of our system is its ability to enhance drug accumulation at the target site while reducing systemic off-target effects or progressive accumulation in organs such as the liver or kidneys. Indeed, systemic injection of unbound LNA-21 led to broad suppression of miR-21 across multiple organs, reinforcing the need for a more targeted approach. The use of an external magnet to guide the SPION-LNA-21 conjugate to the heart, followed by AMF application, significantly improved cardiac specificity and minimized off-target effects. This method significantly decreased the reduction of miR-21 expression in non-cardiac organs, while achieving complete normalization of miR-21 levels in the heart.

In a therapeutic *in vivo* setting, the SPION-based approach and naked LNA resulted in comparable levels of miR-21 knockdown in the heart, while the SPION system led to improved physiological outcomes, including reduced cardiac fibrosis and hypertrophy. This apparent discrepancy may be explained by the distinct pharmacokinetic profiles and the precise control of LNA release afforded by the SPION platform. The SPION system minimizes systemic exposure and off-target knockdown, ensuring that LNA-21 is predominantly active in the myocardium, where it can exert its therapeutic effects. In contrast, systemic LNA administration leads to widespread miR-21 inhibition across multiple organs, which, while effective in the heart, may also elicit off-target effects in non-cardiac tissues, potentially blunting beneficial adaptive responses. The ability of the SPION system to focus therapeutic activity in the target tissue while minimizing adverse effects in other organs could therefore explain the observed improvements in cardiac function and structural remodeling. This highlights the importance of not only achieving target knockdown but also optimizing the spatiotemporal control of drug delivery for improved therapeutic efficacy.

The SPION-based system offers a promising advancement in controlled RNA delivery, particularly through its ability to reduce systemic exposure and enhance targeted release. While it may not yet outperform lipid-based systems in terms of on-target uptake, its precise control over drug activation could provide a unique therapeutic advantage, particularly for conditions requiring spatiotemporal precision. Further studies, including comparisons with

Figure 4. Proof-of-principle *in vivo* study

(A) Human blood was spiked with different doses of SPIONs, and an alternating magnetic field (AMF) was applied. The temperature profile and (B and C) serum concentrations of potassium and LDH were evaluated. $N = 3$ independent experiments. (D) Experimental design of the *in vivo* study. Mice were intravenously injected with PBS, LNA-21 (20 mg/kg body weight), SPIONs (5 mg iron), and SPION-LNA-21 conjugate (5 mg iron), and organs were harvested after 7 days. (E) miRNA-21 expression was measured in different organs 7 days post-injection. Data are presented as mean ± SEM, $N = 7$ (PBS group), $n = 5$ (SPION group), $n = 6$ (SPION-LNA-21 group), and $n = 3$ (LNA-21 group); one-way ANOVA with Bonferroni's multiple comparison test. (F) The probability of survival was analyzed via a Kaplan-Meier survival curve. Inflammatory markers such as interleukin-6 (G) and tissue necrosis factor alpha (TNF-α) (H)) were measured via qRT-PCR in heart tissue after 7 days. $N = 7$ (PBS group), $n = 5$ (SPION group), $n = 6$ (SPION-LNA-21 group), and $n = 3$ (LNA-21 group). One-way ANOVA with Dunnett's multiple comparison test. Data are presented as mean ± SEM. Plasma concentrations of liver (ALT = alanine aminotransferase [I], AST = aspartate aminotransferase [J])) and kidney failure (creatinine [K], urea [L]) were measured via the Cobas 8000 system. $N = 6$ (PBS group), $n = 5$ (SPION group), $n = 6$ (SPION-LNA-21 group), and $n = 3$ (LNA-21 group), Data are presented as mean ± SEM; $^{**}p \leq 0.01$, $^{***}p \leq 0.001$; $^{****}p \leq 0.0001$.

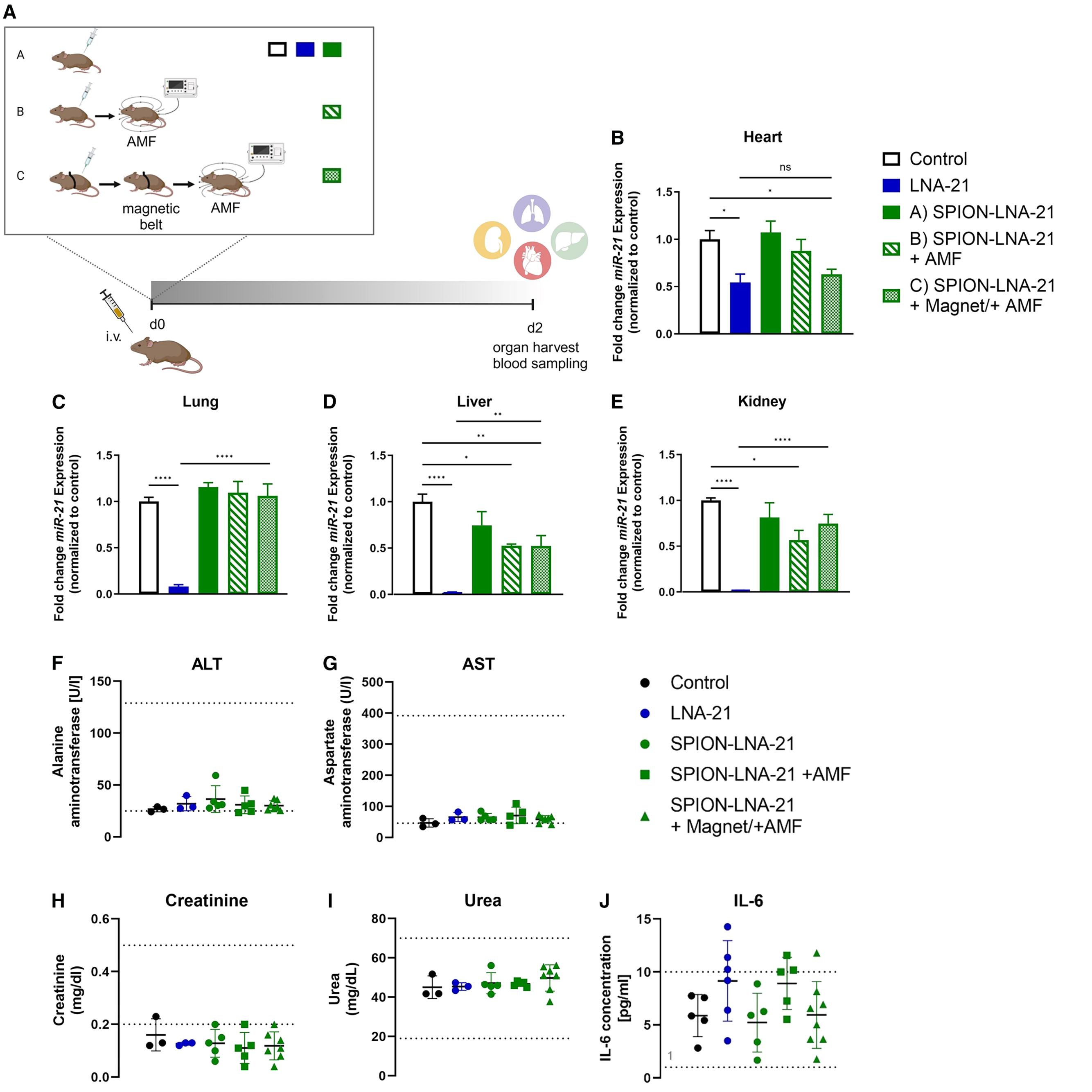

Figure 5. *In vivo* efficacy study

(A) Study design of the *in vivo* study. Mice were either intravenously injected with PBS, LNA-21 (2.5 mg/kg body weight), or SPION-LNA-21 conjugate (2.5 mg/kg body weight) (group A); injected with SPION-LNA-21 conjugate (2.5 mg/kg body weight) followed by application of an alternating magnetic field (AMF, 25 mT, 397 Hz; group B); or injected with SPION-LNA-21 conjugate (2.5 mg/kg body weight) and treated with a combination of an external magnetic belt on the heart during injection and subsequent application of an AMF (group C). Organs were harvested after 2 days, and miRNA-21 expression levels were measured in the heart (B), lung (C), liver (D), and kidney (E). *N* = 6 animals in the PBS group, *n* = 6 animals in the LNA-21 group, *n* = 5 animals in the SPION-LNA-21 group, *n* = 5 animals in the SPION-LNA-21 + AMF group, and *n* = 8 animals in the SPION-LNA-21 +magnet +AMF group. Data are presented as mean ± SEM. One-way ANOVA with Tukey multiple comparison test. Plasma concentrations of liver markers (ALT = alanine aminotransferase [F], AST = aspartate aminotransferase [G]), kidney function markers (creatinine [H] and urea [I]), as well as the inflammatory marker interleukin-6 (Il-6) (J), were measured. *N* = 3–4 animals in the PBS group, *n* = 3 animals in the LNA-21 group, *n* = 5 animals in the SPION-LNA-21 group, *n* = 5 animals in the SPION-LNA-21 + AMF group, and *n* = 7 animals in the SPION-LNA-21 +magnet +AMF group. $^{*}p \leq 0.05$; $^{**}p \leq 0.01$; $^{***}p \leq 0.001$; $^{****}p \leq 0.0001$; ns = not significant.

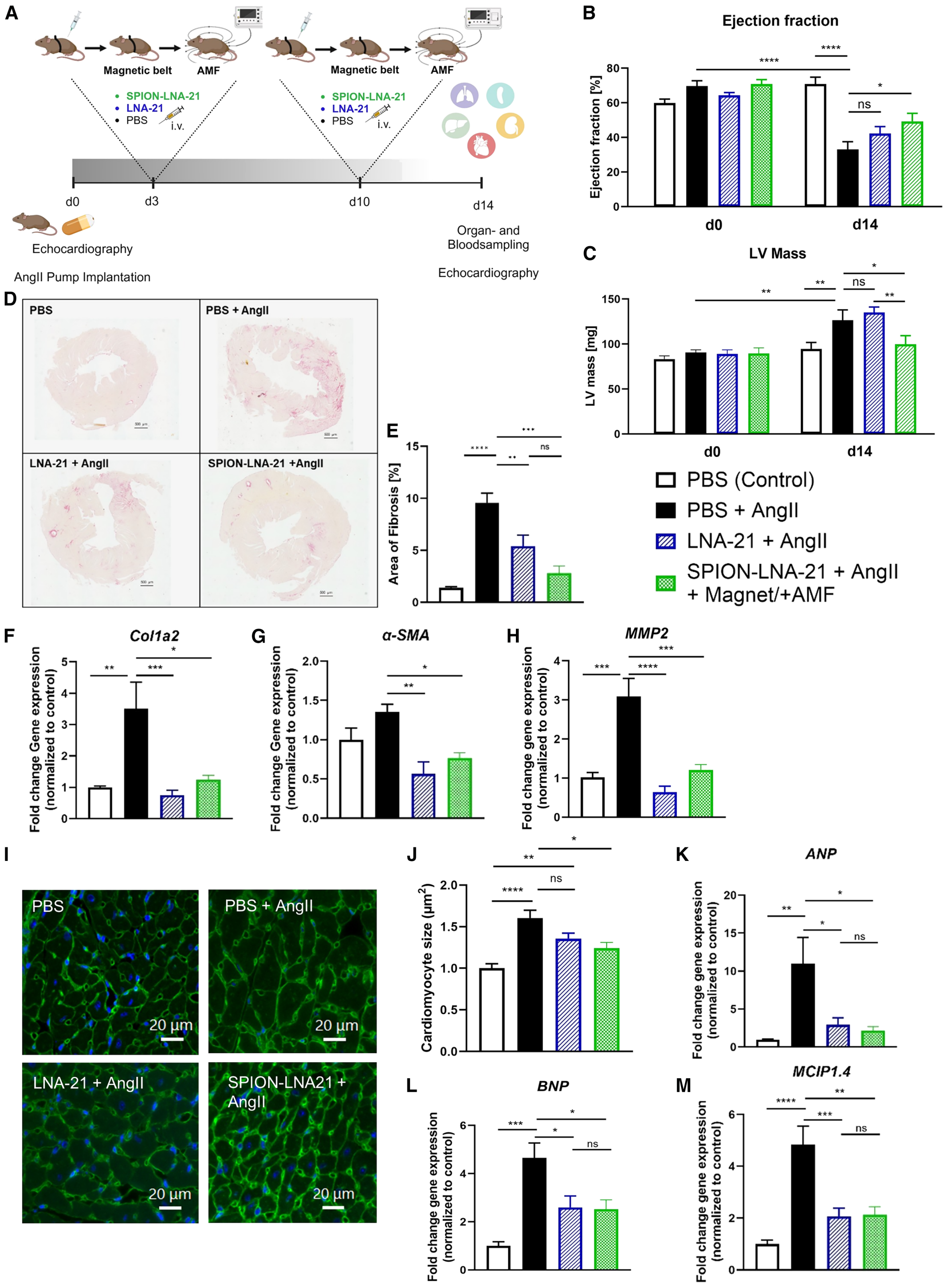

(legend on next page)

established lipid-based systems, will be essential to fully characterize the advantages and limitations of this novel delivery platform.

Despite the advantages of this system, precise quantification of the LNA payload per SPION remains a methodological challenge due to the particle size of the conjugate, which precludes direct mass spectrometry analysis. In addition, the exact determination of the percentage of released drug in relation to the overall functionalization of the particle surface is still a general problem in the field. Further research is needed to explore whether fine-tuning the magnetic field strength and duration of AMF exposure could enable partial LNA release, allowing for more precise secondary dosing beyond the initial intravenous application.

The feasibility of magnetically guided nanoparticle heating has already been demonstrated in a mouse model by Tay et al.,[33] who achieved selective heating of target regions while preserving adjacent healthy tissues. Future studies could incorporate magnetic particle imaging (MPI)[34,35] to enable real-time visualization and control of localized heating, thereby ensuring highly precise LNA release within the region of interest.

While miRNA expression levels vary across tissues, miR-21 was intentionally chosen as a disease-relevant proof-of-concept target with well-established functional and translational significance in cardiac fibrosis. The focus of this study was therefore on demonstrating controlled, therapeutically effective target engagement rather than on transcript-agnostic biodistribution benchmarking. We acknowledge that ubiquitously expressed reference transcripts, such as the long non-coding RNA Malat1, are commonly used in the antisense field to assess tissue distribution independent of target biology. Future studies incorporating such benchmark targets will be important to further delineate the biodistribution characteristics and generalizability of the SPION-based delivery platform. Beyond cardiac applications, our actively controllable, SPION-based drug delivery platform holds broad therapeutic potential. By modulating the positioning of the external magnetic field, the concept can be readily adapted to target a range of organs and disease sites. Additionally, as long as the active agent possesses an amino or, in principle, an alcohol functional group, alternative therapeutics could be conjugated to the SPION-linker system, expanding its use beyond ncRNA inhibitors.

In summary, we have developed a first-of-its-kind, externally controllable, SPION-based drug delivery system that enables targeted RNA inhibition with minimized off-target effects. This innovative approach not only enhances therapeutic precision but also reduces systemic immune activation and toxicity, making it a promising candidate for clinical translation in RNA-based therapies.

MATERIALS AND METHODS

Magnetic characterization and drug release monitoring

ACS

ACS measurements were conducted to analyze the magnetic relaxation properties of the SPION conjugate. In ACS, an AMF is applied over a range of frequencies, and the complex susceptibility ($\chi = \chi'$-$+i\chi''$) is recorded. The real part (χ') reflects the in-phase response, while the imaginary part (χ'') represents energy dissipation and peaks at the characteristic relaxation frequency, $f = \frac{\omega}{2\pi}$, of the particles ($\omega\tau = 1$). For particles primarily undergoing Brownian relaxation, the peak position in χ'' is linked to the Brownian relaxation time constant, τ_B, which is given by $\tau_B = \frac{3\eta V_h}{k_T T}$, where η represents the dynamic viscosity of the medium, V_h is the hydrodynamic volume of the particle, k_B is the Boltzmann constant, and T is the absolute temperature. From this relation, the hydrodynamic diameter can be derived.

MPS

MPS was employed as a complementary technique to monitor changes in the magnetic relaxation behavior associated with drug release. MPS measures the nonlinear magnetization response of magnetic nanoparticles exposed to a high-amplitude AMF. In the low-frequency (LF) regime, typically below 1 kHz, MPS is particularly sensitive to changes in hydrodynamic size, similar to ACS. This makes MPS a suitable tool for monitoring drug release dynamics via relaxation-based signal changes, as described by Draack

Figure 6. Evaluation of therapeutic effects in the angiotensin-II mouse model

(A) Study design of the *in vivo* study. On day 0, an osmotic pump was subcutaneously implanted to constantly release angiotensin-II (3 mg/kg/day) over a period of 14 days. Control mice were sham-operated. On day 3 and 10, mice were intravenously injected with PBS, LNA-21 (2.5 mg/kg body weight), or SPION-LNA-21 conjugate (2.5 mg/kg body weight), with a magnetic belt placed on the heart during injection and subsequent application of an alternating magnetic field (AMF, 25 mT, 397 Hz) to release the LNA. Echocardiographic parameter, s ejection fraction (B) and left ventricular (LV) mass (C), were measured on day 0 and day 14 and then calculated. Data are presented as mean ± SEM; $N = 6$ in the PBS group, $n = 7$ in the PBS+AngII group, $n = 9$ in the LNA-21+AngII group, and $n = 5$ in the SPION-LNA-21 + AngII group. One-way-ANOVA with Sidak's multiple comparison test. (D) Microscopic images of Picro-Sirius Red staining (PSR) on heart sections. Representative images from $n = 5$–9 animals per group. (E) Quantification of fibrosis levels in heart sections stained with PSR. Data are presented as mean ± SEM; one-way ANOVA with Tukey's multiple comparison test; $N = 6$ in the PBS group, $n = 7$ in the PBS+AngII group, $n = 9$ in the LNA-21+AngII group, and $n = 5$ in the SPION-LNA-21 + AngII group. Gene expression of fibrosis-associated genes, collagen 1a2 (Col1a2) (F), α-smooth muscle actin (a-SMA) (G), and matrix metalloproteinase 2 (MMP2) (H), was measured. Data are presented as mean ± SEM; one-way ANOVA with Tukey's multiple comparison test; $N = 6$ in the PBS group, $n = 7$ in the PBS+AngII group, $n = 9$ in the LNA-21+AngII group, and $n = 5$ in the SPION-LNA-21 + AngII group. (I) Confocal images of wheat-agglutinin (WGA) staining on heart sections. Representative images from $n = 5$–9 animals per group. (J) Quantification of cardiomyocyte size in heart sections stained with WGA. Data are presented as mean ± SEM; one-way ANOVA with Tukey's multiple comparison test; $N = 5$–9 animals per group. Gene expression of hypertrophy-associated genes, ANP (atrial natriuretic peptide (K), BNP (brain natriuretic peptide) (L), and MCIP (myocyte-enriched calcineurin-interacting protein (M), was analyzed in heart tissue. Data are presented as mean ± SEM; one-way ANOVA with Tukey's multiple comparison test; $N = 6$ in the PBS group, $n = 7$ in the PBS+AngII group, $n = 9$ in the LNA-21+AngII group, and $n = 5$ in the SPION-LNA-21 + AngII group. *$p \leq 0.05$; **$p \leq 0.01$; ***$p \leq 0.001$; ****$p \leq 0.0001$; ns = not significant.

et al. *Drug-release study with MPS:* Samples were initially heated externally to 80°C using a Peltier-tempered sample holder directly in the MPS. Subsequently, an AMF of 25 kHz frequency and 25 mT amplitude was applied to induce hysteresis heating in the nanoparticles while keeping the sample solution at a constant temperature. This hysteresis heating mechanism is due to the irreversible rotation of magnetic moments in response to the AMF. At predetermined intervals, the AMF was briefly paused to perform LF-MPS measurements, enabling real-time tracking of changes in relaxation behavior to monitor drug release kinetics over time.

AMF

The AMF was generated using the TruHeat HF of the AXIO 10/450 series (Trumpf Hüttinger). The system is supported by a booster pump to ensure constant water pressure during operation. The following inductive coils are used to generate the magnetic field: (1) a coil with four windings and an internal diameter of 5.5 cm, for *in vivo* studies, and (2) a coil with six windings and an internal diameter of 3.0 cm, for *in vitro* studies.

Field strengths of 20, 25, and 50 mT (397 Hz) were used, as stated in the text.

In vitro and *in vivo* experiments

Cell culture, transfection, and treatment

Human cardiac fibroblasts (HCFs, Promega) were cultured in fibroblast basal medium (FBM)-3 supplemented with 10% fetal bovine serum (FBS), supplements (Promega), 100 μg/mL penicillin, and 100 μg/mL streptomycin under standard cell culture conditions (37°C, 5% CO_2). For analysis of release kinetics, the SPION-LNA-21 conjugate was subjected to an AMF at different field strengths for different durations, and HCF cells were afterward transfected with different doses of the nanoparticle construct (100 and 200 nM), the respective amount of unbound LNA-21 and unheated SPION-LNA-21 for 48 h.

NRK49F cells were cultivated in low-glucose DMEM (Lonza, Basel, Switzerland) supplemented with 10% FBS, 100 μg/mL penicillin, and 100 μg/mL streptomycin under standard cell culture conditions (37°C, 5% CO_2). Neonatal mouse cardiomyocytes (NMCMs) were kept in MEM with 5% FBS at 37°C and 1% CO_2. HepG2 cells were cultivated in RPMI medium (Gibco) with 10% FBS under standard cell culture conditions (37°C, 5% CO_2).

Cytotoxicity assessment

The WST-1 assay (Roche) was used to measure the metabolic activity of HCF cells after treatment with Perimag SPIONs, according to the manufacturer's instructions.

The CytoTox 96 Non-Radioactive Cytotoxicity Kit (Promega) was used to analyze LDH release in HCF, NRK49F, NMCM, and HepG2 cells after treatment with different doses of SPIONs, linker fragments, and SPION-LNA-21 conjugate (+AMF) for 48 h, according to the manufacturer's instructions.

Using the Caspase-Glo 3/7 Assay (Promega), the apoptosis rate was assessed in HCF cells after treatment with different doses of SPIONs, linker fragments, and SPION-LNA-21 conjugate (+AMF) for 48 h, according to the manufacturer's instructions.

RNA isolation

Total RNA from tissues and cultured cells was isolated using the RNeasy Mini Kit (Qiagen) or the Qiazol method (Qiagen), according to the manufacturer's instructions. Subsequent quantification and quality control were performed with the Synergy HT Reader (BioTek).

Quantitative real-time PCR

For cDNA synthesis, the TaqMan MicroRNA Reverse Transcription Kit (Applied Biosystems) was used according to the manufacturer's instructions. Quantification of specific miRNA levels was performed by quantitative real-time PCR using the TaqMan MicroRNA Assay (Applied Biosystems). qPCR was performed in a 384-well PCR plate with the ViiA 7 Real-Time PCR System (Thermo Fisher Scientific). The analysis was performed using the $\Delta\Delta$ct method. miRNA levels were normalized to the small RNA molecule snoRNA-202 and U6 for mouse samples and to RNU48 for human samples. The Taqman assays used are listed in Table 1.

For quantitative detection of mRNAs, reverse transcription of total RNA prior to real-time qPCR was performed using the iScript Select cDNA synthesis kit (Bio-Rad), according to the manufacturer's instructions. Real-time qPCR was performed in a CFX96 Touch Real-Time PCR Detection System (Bio-Rad) using specific primers (Table 1) and the iQ SYBR Green Mix (Bio-Rad), according to the manufacturer's protocol. Glyceraldehyde-3-phosphate dehydrogenase (GAPDH) or 18S ribosomal RNA (18S rRNA) was used as a housekeeping control for gene-specific expression levels.

IL-6 ELISA

For the quantification of IL-6 levels in mouse plasma, the Mouse IL-6 high sensitivity ELISA (Invitrogen) was performed according to the manufacturer's instructions.

Dynamic light scattering

The hydrodynamic diameter of SPION-LNA-21 conjugates was characterized using dynamic light scattering (Zetasizer Nano ZS, Malvern Panalytical).

Statistics

All *in vitro* experiments were performed as indicated in the corresponding figure legends. If not labeled separately, three biological replicates were used for each independent experiment. Of these, three technical replicates were performed. Data are expressed as the mean of the independent samples ±standard error of the mean (SEM). Statistical analysis was performed using GraphPad Prism. For the statistical comparison of two groups, an unpaired, two-tailed Student's *t* test was performed. For the comparison of three or more groups, a one-way ANOVA followed by the stated post-test was performed.

Table 1. Sequences of LNA, primers, and TaqMan assays

LNA Sequence	
LNA-21	5′-/5AmMC6/TCAGTCTGATAAGCT-3′
TaqMan Primer assays	
Rnu48	ID: 001006
U6 snRNA	ID: 001973
Sno202	ID: 001232
miR-21	ID: 000397
Primer Sequences	
mmu_GAPDH	Forward:5′TTCACCACCATGGAGAAGGC3′; Reverse:5′GGCATGGACTGTGGTCATGA3′
mmu_18S	Forward: 5′ GTAACCCGTTGAACCCCATT 3′; Reverse: 5′CCATCCAATCGGTAGTAGCG 3′
mmu_aSMA	Forward:5′ACTACTGCCGAGCGTGAGAT3′; Reverse:5′AAGGTAGACAGCGAAGCCAG3′
mmu_ANP	Forward:5′CCTGTGTACAGTGCGGTGTC3′; Reverse:5′CCTAGAAGCACTGCCGTCTC3′
mmu_BNP	Forward:5′CTGAAGGTGCTGTCCCAGAT3′; Reverse:5′GTTCTTTTGTGAGGCCTTGG3′
mmu_col1a2	Forward:5′CAGAACATCACCTACCACTGCA3′; Reverse:5′TTCAACATCGTTGGAACCCTG3′
mmu_Il-1b	Forward:5′TGCCACCTTTTGACAGTGATG3′; Reverse:5′ATGTGCTGCTGCGAGATTTG3′
mmu_Il-6	Forward: 5′ AGCCAGAGTCCTTCAGAGAGAT 3′; Reverse: 5′ GAGAGCATTGGAAATTGGGGT 3′
mmu_MCIP	Forward:5′AGGGACTTTAGCTACAATTT 3′; Reverse:5′TATGTTCTGAAGAGGGATTC 3′
mmu_MMP2	Forward:5′GCCTCATACACAGCGTCAATCTT3′; Reverse:5′CGGTTTATTTGGCGGACAGT3′
mmu_TNF-a	Forward:5′TACTGAACTTCGGGGTGATTGGTCC3′; Reverse:5′CAGCCTTGTCCCTTGAAGAGAACC3′

Animal experiments

All animal studies involving mice were performed in accordance with the relevant guidelines and regulations and with the approval of the Niedersächsisches Landesamt für Verbraucherschutz und Lebensmittelsicherheit (LAVES, Germany, TVA-ID 21/03636 and 2022/294). For all *in vivo* experiments, male C57BL/6NCrl wild-type mice (Charles River Laboratories, Germany) aged 8–10 weeks were used.

For the proof-of-principle study, mice were intravenously injected with PBS, 5 mg of Perimag SPIONs, 5 mg of SPION-LNA-21 conjugate, or LNA-21 (20 mg/kg body weight), and organs were harvested after 7 days.

For the efficacy study, mice were either intravenously injected with PBS, LNA-21 (2.5 mg/kg body weight), or SPION-LNA-21 conjugate (2.5 mg/kg body weight with respect to LNA-21 concentration) (group A); intravenously injected with SPION-LNA-21 conjugate (2.5 mg/kg body weight with respect to LNA-21 concentration), followed by application of an AMF (25 mT, 397 Hz for 30 min; group B); or injected with SPION-LNA-21 conjugate (2.5 mg/kg body weight with respect to LNA-21 concentration) and treated with a combination of an external magnetic belt placed on the heart during injection, and for a further 30 min, followed by subsequent application of an AMF (25 mT, 397 Hz for 30 min; group C). Organs were harvested after 2 days.

To induce systemic hypertension for the therapeutic study, subcutaneous implantation of osmotic minipumps (ALZET) delivering Ang II at 3 mg/kg BW/day was performed. The operation was performed under general anesthesia with isoflurane (2%–4% isoflurane in 0.8 L/min oxygen). After subcutaneous infiltration analgesia with lidocain and bupivacain (each 2 mg/kg BW), the minipump (ALZET micro-osmotic pump model 1002) was implanted in a subcutaneous pouch on the back. Analgesia was complemented with four carprofen injections (10 mg/kg BW) before surgery and 12, 24, and 48 h after operation. On the third and tenth day after pump implantation, mice were either treated with pure LNA-21 (2.5 mg/kg body weight) or with the SPION-LNA-21 conjugate (2.5 mg/kg body weight with respect to LNA-21 concentration). To enhance cardiac targeting, a neodymium permanent magnet was placed over the heart for 30 min during and after injection to enrich the nanoparticles in the myocardium before exposure to an AMF (25 mT, 397 Hz) for 30 min. Control animals received PBS injections, as well as sham-operated controls.

Cardiac function was assessed by echocardiography (Vevo2100, Fujifilm/VisualSonics, Canada) under general anesthesia with isoflurane (see above). Echocardiography data were analyzed using standard imaging protocols (M-mode and B-mode) for global cardiac volumes and function using Vevo LAB 3.2.0 (Fujifilm Visualsonics, Inc).

Isolation of neonatal cardiomyocytes from mice

To isolate cardiomyocytes, hearts from 0.5- to 2-day-old mice were removed and washed twice in PBS (with added 100 U/mL Pen/Strep). After transferring up to 40 minced mouse hearts into a

gentleMACS C tube (Miltenyi Biotec), two additional washes with PBS were performed. Two enzyme mixtures, enzyme mixture 1: 125 μL enzyme P + 4600 μL buffer X, and enzyme mixture 2: 50 μL buffer Y + 25 μL enzyme A + 200 μL enzyme D, were mixed together and transferred to the gentleMACS C Tube, and the tissue was dissociated via the gentleMACS Octo Dissociator (Miltenyi Biotec). Next, the tubes were removed, and culture medium (MEM [Bioconcept], 5% FBS [Gibco], 292 mg/L L-glutamine [Sigma-Aldrich], 350 mg/L $NaHCO_3$ [Sigma-Aldrich], 1 mL/L vitamin B12 [Sigma-Aldrich], 5 mL BrdU [Sigma-Aldrich], and 10 mL P/S [Promocell]) was added. The suspension was homogenized and transferred to a 70 μm MACS SmartStrainer. After washing, the cells were centrifuged at 600 × g for 5 min at room temperature. The supernatant was removed, and the cell pellet was resuspended in medium. The suspension was placed in a Petri dish and incubated for 90 min at 37°C and 1% CO_2. During the incubation time, the required cell culture plates were coated with 0.1% gelatin (Sigma-Aldrich) and incubated for 45 min. Next, the cardiomyocytes were obtained from the supernatant. After further washing the Petri dish with medium, the cells were seeded onto the plates.

Histology

For histological assessment of cardiac fibrosis in mice, paraffin-embedded sections of the LV were stained with Picro-Sirius Red, and the collagen content was calculated as the percentage of fibrotic areas in the heart. Quantifications of microscopic images were performed with the BZ-X800 Analyzer (Keyence).

For murine cardiomyocyte size measurement, cardiomyocyte cell membranes in the myocardium were visualized by wheat germ agglutinin staining coupled to Alexa Fluor 488 (Invitrogen). The area of cardiomyocytes was calculated using ImageJ Fiji.

For biodistribution analysis of SPIONs, iron content was stainend in liver, spleen, and heart tissue via Perl's Prussian blue staining according to the manufacturer's instructions (Abcam).

Plasma sampling and biochemical analysis

EDTA-plasma samples were drawn from the mice and centrifuged at 3000 × g for 10 min. The supernatant was stored at −80°C until analysis. Laboratory parameters were determined on a Cobas 8000 Modul c701and Cobas c111 using standard methods (Roche): creatinine, urea, ALT, and AST.

DATA AND CODE AVAILABILITY

The data underlying this article are available in the article and in its online supplemental information.

ACKNOWLEDGMENTS

The authors acknowledge valuable discussions with Frank Ludwig and measurement support provided by Deike Hicken. Furthermore, the authors acknowledge DLS measurement support from Sedef Ersoy, WGA measurement support from Nila Stieber, NMR measurements by Linn Müggenburg and Jörg Fohrer, and and laboratory parameter measurement support from Lichtinghagen, Leifheit-Nestler, and MartinaThiele. This project received funding from the European Union's Horizon 2020 research and innovation program under grant agreement no. 825670, named Cardioregenix (to T.T.) and financial support from the German Research Foundation under grant no. VI 892/4-1 (to T.V.). A patent application regarding the RNA delivery technology has been filed. T.T. is founder and CSO/CMO of Cardior Pharmaceuticals GmbH, a wholly-owned subsidiary of Novo Nordisk Europe A/S.

AUTHOR CONTRIBUTIONS

T.T. and A.K. conceived the project. F.K. and T.T. designed the biological part of the experiments, and A.K. and G.D. designed the chemical part. The synthesis of the SPION-LNA-21 conjugate system was developed and performed throughout the project by K.H., whereas synthesis for the angiotensin study was performed by A.S. *In vitro/ex vivo* experiments were performed and analyzed by F.K. together with L.P.J.H., K.H., and S.G. F.K., L.P.J.H., K.H., J.B., G.B., K.J., and C.B. designed and performed the *in vivo* studies. F.K., L.P.J.H., and A.J. carried out the histological analysis. T.V. examined the magnetic properties of the SPION system (ACS/MPS) and provided the expertise to design the SPION subtype for the synthesis. A.G., S.T., and A.P. performed RNA isolations and qPCRs throughout the project. F.K., G.D., A.K., and T.T. supervised the research. F.K., K.H., L.P.J.H., and T.T. wrote the manuscript. All authors read and approved the final paper.

DECLARATION OF INTERESTS

T.T. is founder and CSO/CMO of Cardior Pharmaceuticals GmbH, a wholly owned subsidiary of Novo Nordisk Europe A/S.

SUPPLEMENTAL INFORMATION

Supplemental information can be found online at https://doi.org/10.1016/j.omtn.2026.102902.

REFERENCES

1. Täubel, J., Hauke, W., Rump, S., Viereck, J., Batkai, S., Poetzsch, J., Rode, L., Weigt, H., Genschel, C., Lorch, U., et al. (2021). Novel antisense therapy targeting microRNA-132 in patients with heart failure: results of a first-in-human Phase 1b randomized, double-blind, placebo-controlled study. Eur. Heart J. *42*, 178–188. https://doi.org/10.1093/eurheartj/ehaa898.

2. Bauersachs, J., Solomon, S.D., Anker, S.D., Antorrena-Miranda, I., Batkai, S., Viereck, J., Rump, S., Filippatos, G., Granzer, U., Ponikowski, P., et al. (2024). Efficacy and safety of CDR132L in patients with reduced left ventricular ejection fraction after myocardial infarction: Rationale and design of the HF-REVERT trial. Eur. J. Heart Fail. *26*, 674–682. https://doi.org/10.1002/ejhf.3139.

3. Huang, X., Kong, N., Zhang, X., Cao, Y., Langer, R., and Tao, W. (2022). The landscape of mRNA nanomedicine. Nat. Med. *28*, 2273–2287. https://doi.org/10.1038/s41591-022-02061-1.

4. Winkle, M., El-Daly, S.M., Fabbri, M., and Calin, G.A. (2021). Noncoding RNA therapeutics—challenges and potential solutions. Nat. Rev. Drug Discov. *20*, 629–651. https://doi.org/10.1038/s41573-021-00219-z.

5. Huang, C.-K., Kafert-Kasting, S., and Thum, T. (2020). Preclinical and Clinical Development of Noncoding RNA Therapeutics for Cardiovascular Disease. Circ. Res. *126*, 663–678. https://doi.org/10.1161/CIRCRESAHA.119.315856.

6. Bajan, S., and Hutvagner, G. (2020). RNA-Based Therapeutics: From Antisense Oligonucleotides to miRNAs. Cells *9*, 137. https://doi.org/10.3390/cells9010137.

7. Lucht, N., Friedrich, R.P., Draack, S., Alexiou, C., Viereck, T., Ludwig, F., and Hankiewicz, B. (2019). Biophysical Characterization of (Silica-coated) Cobalt Ferrite Nanoparticles for Hyperthermia Treatment. Nanomaterials *9*, 1713. https://doi.org/10.3390/nano9121713.

8. Engelmann, U.M., Buhl, E.M., Draack, S., Viereck, T., Ludwig, F., Schmitz-Rode, T., and Slabu, I. (2018). Magnetic Relaxation of Agglomerated and Immobilized Iron Oxide Nanoparticles for Hyperthermia and Imaging Applications. IEEE Magn. Lett. *9*, 1–5. https://doi.org/10.1109/LMAG.2018.2879034.

9. Mancuso, L., Knobloch, T., Buchholz, J., Hartwig, J., Möller, L., Seidel, K., Collisi, W., Sasse, F., and Kirschning, A. (2014). Preparation of Thermocleavable Conjugates Based on Ansamitocin and Superparamagnetic Nanostructured Particles by a Chemobiosynthetic Approach. Chem. Eur J. *20*, 17541–17551. https://doi.org/10.1002/chem.201404502.

10. Wang, L.L., Balakrishnan, A., Bigall, N.C., Candito, D., Miethe, J.F., Seidel, K., Xie, Y., Ott, M., and Kirschning, A. (2017). A Bio-Chemosynthetic Approach to Superparamagnetic Iron Oxide–Ansamitocin Conjugates for Use in Magnetic Drug Targeting. Chem. Eur J. *23*, 2265–2270. https://doi.org/10.1002/chem.201604903.

11. Ullah, S., Seidel, K., Türkkan, S., Warwas, D.P., Dubich, T., Rohde, M., Hauser, H., Behrens, P., Kirschning, A., Köster, M., et al. (2019). Macrophage entrapped silica coated superparamagnetic iron oxide particles for controlled drug release in a 3D cancer model. J. Controlled Release *294*, 327–336. https://doi.org/10.1016/j.jconrel.2018.12.040.

12. Norris, M.D., Seidel, K., and Kirschning, A. (2019). Externally Induced Drug Release Systems with Magnetic Nanoparticle Carriers: An Emerging Field in Nanomedicine. Adv. Ther. *2*, 1800092. https://doi.org/10.1002/adtp.201800092.

13. Carter, T.J., Agliardi, G., Lin, F.Y., Ellis, M., Jones, C., Robson, M., Richard-Londt, A., Southern, P., Lythgoe, M., Zaw Thin, M., et al. (2021). Potential of Magnetic Hyperthermia to Stimulate Localized Immune Activation. Small *17*, 2005241. https://doi.org/10.1002/smll.202005241.

14. Yang, C.-T., Korangath, P., Stewart, J., Hu, C., Fu, W., Grüttner, C., Beck, S.E., Lin, F.-H., and Ivkov, R. (2020). Systemically delivered antibody-labeled magnetic iron oxide nanoparticles are less toxic than plain nanoparticles when activated by alternating magnetic fields. Int. J. Hyperthermia *37*, 59–75. https://doi.org/10.1080/02656736.2020.1776901.

15. Grüttner, C., Müller, K., Teller, J., and Westphal, F. (2013). Synthesis and functionalisation of magnetic nanoparticles for hyperthermia applications. Int. J. Hyperthermia *29*, 777–789. https://doi.org/10.3109/02656736.2013.835876.

16. Kut, C., Zhang, Y., Hedayati, M., Zhou, H., Cornejo, C., Bordelon, D., Mihalic, J., Wabler, M., Burghardt, E., Gruettner, C., et al. (2012). Preliminary Study of Injury From Heating Systemically Delivered, Nontargeted Dextran–Superparamagnetic Iron Oxide Nanoparticles in Mice. Nanomedicine *7*, 1697–1711. https://doi.org/10.2217/nnm.12.65.

17. Wang, G., Li, C., Li, J., and Jia, X. (2009). Catalyst-free water-mediated N-Boc deprotection. Tetrahedron Lett. *50*, 1438–1440. https://doi.org/10.1016/j.tetlet.2009.01.056.

18. Wang, J., Liang, Y.-L., and Qu, J. (2009). Boiling water-catalyzed neutral and selective N-Boc deprotection. Chem. Commun. *5144*, 5144–5146. https://doi.org/10.1039/b910239f.

19. Knipp, R.J., Estrada, R., Sethu, P., and Nantz, M.H. (2014). Thermally induced substrate release via intramolecular cyclizations of Amino esters and Amino carbonates. Tetrahedron *70*, 3422–3429. https://doi.org/10.1016/j.tet.2014.03.092.

20. Thum, T., Gross, C., Fiedler, J., Fischer, T., Kissler, S., Bussen, M., Galuppo, P., Just, S., Rottbauer, W., Frantz, S., et al. (2008). MicroRNA-21 contributes to myocardial disease by stimulating MAP kinase signalling in fibroblasts. Nature *456*, 980–984. https://doi.org/10.1038/nature07511.

21. Hinkel, R., Ramanujam, D., Kaczmarek, V., Howe, A., Klett, K., Beck, C., Dueck, A., Thum, T., Laugwitz, K.-L., Maegdefessel, L., et al. (2020). AntimiR-21 Prevents Myocardial Dysfunction in a Pig Model of Ischemia/Reperfusion Injury. J. Am. Coll. Cardiol. *75*, 1788–1800. https://doi.org/10.1016/j.jacc.2020.02.041.

22. Gupta, S.K., Itagaki, R., Zheng, X., Batkai, S., Thum, S., Ahmad, F., Van Aelst, L.N., Sharma, A., Piccoli, M.-T., Weinberger, F., et al. (2016). miR-21 promotes fibrosis in an acute cardiac allograft transplantation model. Cardiovasc. Res. *110*, 215–226. https://doi.org/10.1093/cvr/cvw030.

23. Grüttner, C., Müller, K., Teller, J., Westphal, F., Foreman, A., and Ivkov, R. (2007). Synthesis and antibody conjugation of magnetic nanoparticles with improved specific power absorption rates for alternating magnetic field cancer therapy. J. Magn. Magn Mater. *311*, 181–186. https://doi.org/10.1016/j.jmmm.2006.10.1151.

24. Kuri, P., Ellwanger, K., Kufer, T.A., Leptin, M., and Bajoghli, B. (2017). A high-sensitivity bi-directional reporter to monitor NF-κB activity in cell culture and zebrafish in real time. J. Cell Sci. *130*, 648–657. https://doi.org/10.1242/jcs.196485.

25. Romero-Palomo, F., Festag, M., Lenz, B., Schadt, S., Brink, A., Kipar, A., Steinhuber, B., Husser, C., Koller, E., Sewing, S., et al. (2021). Safety, Tissue Distribution, and Metabolism of LNA-Containing Antisense Oligonucleotides in Rats. Toxicol. Pathol. *49*, 1174–1192. https://doi.org/10.1177/01926233211011615.

26. Thum, T., Chau, N., Bhat, B., Gupta, S.K., Linsley, P.S., Bauersachs, J., and Engelhardt, S. (2011). Comparison of different miR-21 inhibitor chemistries in a cardiac disease model. J. Clin. Investig. *121*, 461–463. https://doi.org/10.1172/JCI45938.

27. Yan, L., Su, Y., Hsia, I., Xu, Y., Vincent-Chong, V.K., Mojica, W., Seshadri, M., Zhao, R., and Wu, Y. (2023). Delivery of anti-microRNA-21 by lung-targeted liposomes for pulmonary fibrosis treatment. Mol. Ther. Nucleic Acids *32*, 36–47. https://doi.org/10.1016/j.omtn.2023.02.031.

28. Zhang, J., Jiao, J., Cermelli, S., Muir, K., Jung, K.H., Zou, R., Rashid, A., Gagea, M., Zabludoff, S., Kalluri, R., and Beretta, L. (2015). miR-21 Inhibition Reduces Liver Fibrosis and Prevents Tumor Development by Inducing Apoptosis of CD24+ Progenitor Cells. Cancer Res. *75*, 1859–1867. https://doi.org/10.1158/0008-5472.CAN-14-1254.

29. Gomez, I.G., MacKenna, D.A., Johnson, B.G., Kaimal, V., Roach, A.M., Ren, S., Nakagawa, N., Xin, C., Newitt, R., Pandya, S., et al. (2015). Anti–microRNA-21 oligonucleotides prevent Alport nephropathy progression by stimulating metabolic pathways. J. Clin. Investig. *125*, 141–156. https://doi.org/10.1172/JCI75852.

30. Kölling, M., Kaucsar, T., Schauerte, C., Hübner, A., Dettling, A., Park, J.-K., Busch, M., Wulff, X., Meier, M., Scherf, K., et al. (2017). Therapeutic miR-21 Silencing Ameliorates Diabetic Kidney Disease in Mice. Mol. Ther. *25*, 165–180. https://doi.org/10.1016/j.ymthe.2016.08.001.

31. Lorenzen, J.M., Haller, H., and Thum, T. (2011). MicroRNAs as mediators and therapeutic targets in chronic kidney disease. Nat. Rev. Nephrol. *7*, 286–294. https://doi.org/10.1038/nrneph.2011.26.

32. Buntz, A., Killian, T., Schmid, D., Seul, H., Brinkmann, U., Ravn, J., Lindholm, M., Knoetgen, H., Haucke, V., and Mundigl, O. (2019). Quantitative fluorescence imaging determines the absolute number of locked nucleic acid oligonucleotides needed for suppression of target gene expression. Nucleic Acids Res. *47*, 953–969. https://doi.org/10.1093/nar/gky1158.

33. Tay, Z.W., Chandrasekharan, P., Chiu-Lam, A., Hensley, D.W., Dhavalikar, R., Zhou, X.Y., Yu, E.Y., Goodwill, P.W., Zheng, B., Rinaldi, C., and Conolly, S.M. (2018). Magnetic Particle Imaging-Guided Heating in Vivo Using Gradient Fields for Arbitrary Localization of Magnetic Hyperthermia Therapy. ACS Nano *12*, 3699–3713. https://doi.org/10.1021/acsnano.8b00893.

34. Draack, S., Schilling, M., and Viereck, T. (2021). Magnetic particle imaging of particle dynamics in complex matrix systems. Phys. Sci. Rev. *8*, 213–237. https://doi.org/10.1515/psr-2019-0123.

35. Janssen, K.-J., Zhong, J., Viereck, T., Schilling, M., and Ludwig, F. (2022). Quantitative temperature visualization with single harmonic-based magnetic particle imaging. J. Magn. Magn Mater. *563*, 169915. https://doi.org/10.1016/j.jmmm.2022.169915.

OMTN, Volume *37*

Supplemental information

Time- and spatially resolved

LNA delivery via thermally

controlled SPION technology

Franziska Kenneweg, Katharina Hempel, Lukas Philipp Joachim Höhne, Gerald Dräger, Jonas Blume, Thilo Viereck, Anastasia Stohwasser, Sonja Groß, Gwen Büchler, Karina Jansen, Malte Juchem, Christian Bär, Angelika Pfanne, Annette Just, Sabrina Thum, Anika Gietz, Andreas Kirschning, and Thomas Thum

Supplemental methods

Chemical syntheses:

General information

All materials and reagents were purchased from commercial distributors (Sigma Aldrich, tci, Alfa Aesar, Acros Organics, Carl Roth) and were not further purified unless otherwise stated. Specially prepared reagents were labelled as such. For LC-MS as well as preparative and semi-preparative HPLC, double-distilled water and 0.1 % formic acid were used. Reactions that had to take place in the absence of air and moisture were carried out using the Schlenk technique.

Dialysis

Dialysis of the aqueous reaction solutions was carried out in dialysis tubes of the Membra-Cel™ dialysis membrane type with an exclusion limit of 14,000 Daltons from Carl Roth. The dialysis took place over four to five days, depending on the test specification, at room temperature and with gentle stirring in 5 litre beakers.

Chromatography

Silica gel 60M (particle size 40-63 µm) was purchased from Macherey-Nagel for column chromatography and LiChroprep® RP-18 (40-63 µm) from Merck for reversed-phase chromatography. The respective eluents were purchased in HPLC quality. For thin-layer chromatography, ALUGRAM® Xtra SIL G/UV254 ready-to-use DC films from Macherey-Nagel and ALUGRAM® RP-18W/UV254 ready-to-use DC films from Macherey-Nagel were used. UV-active compounds were visualized at a wavelength of 254 nm using a Macherey-Nagel type 60 fluorescent indicator. Fluorescent compounds

were visualized with the above-mentioned fluorescence indicator at a wavelength of 366 nm. Self-prepared coloring reagents such as potassium permanganate, bromocresol green, vanillin and ninhydrin were used for UV-inactive compounds.

Nuclear magnetic resonance spectroscopy

^{1}H and ^{13}C NMR spectra were recorded at room temperature using the DPX 400 (Bruker), AMX 400 (Bruker), Ascend 400 Avance III HD (Bruker), DRX 500 (Bruker) or Ascend 600 (Bruker) devices. For the ^{1}H NMR spectra, calibration was performed using the residual proton signal of the respective solvent used [δ($CDCl_3$) = 7.26 ppm, δ(CD_3OD) = 3.31 ppm, δ(DMSO-d6) = 2.50 ppm]. The chemical shift δ is given in ppm and the coupling constant J in Hz. The following abbreviations or combinations of these were used for the multiplicities of the signals: s = singlet, d = doublet, t = triplet, q = quartet, m = multiplet, b = broad signal. The ^{13}C NMR spectra were calibrated using the chemical shift of the respective solvent used [δ($CDCl_3$) = 77.16 ppm, δ(CD_3OD) = 49.00 ppm, δ(DMSO-d6) = 39.53 ppm]. The chemical shift is given in ppm. The NMR spectra were analyzed using TopSpin 4.0.8 from Bruker.

High-resolution mass spectrometry

High-resolution mass spectrometry was carried out on the following devices:

- HR UPLC-MS: Waters QTof Premier (ESI- and APCI-MS/MS) with UPLC system (Waters Acquity™ incl. TUV UV-detector)

- HR HPLC-MS: Micromass LCT Premier with HPLC system (Waters Alliance 2695)

- UPLC-MS: Xevo™ QTof MS (Waters Zspray™, ESI) with UPLC system, (Waters Acquity™, incl. photodiode array detector)

The calculated and detected masses are shown.

Aldehyde synthesis

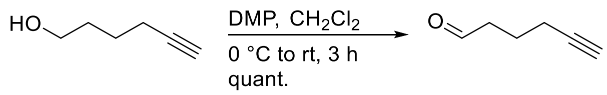

The Dess-Martin-periodinane reagent (2.16 g, 5.10 mmol, 1.00 eq.) in dichloromethane (27.6 mL) was cooled to 0 °C. 5-Hexin-1-ol (0.50 g, 5.10 mmol, 1.00 eq.) was added dropwise and the reaction mixture was warmed up to room temperature after 5 minutes. After 18 hours, the reaction mixture was filtered through a pad of Celite™ (ethyl acetate) and the solvent was removed under reduced pressure. After purification by column chromatography (pentane:diethyl ether 6:1), the aldehyde (0.48 g, 5.00 mmol, 98 %) was obtained as a colorless oil and directly employed in the next step.

Rf = 0.28 (pentane:diethyl ether 10:1);

ESI-HRMS: m/z calculated for C6H8ONa [M+Na]+: 119.1188, found: 119.1197.

Ester synthesis

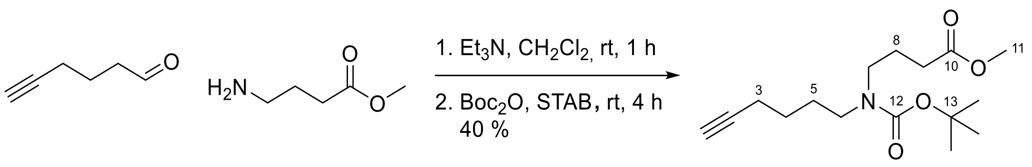

4-Aminobutyric acid methyl ester (585 mg, 5.00 mmol, 1.00 eq.) and triethylamine (1.7 mL, 12.50 mmol, 2.50 eq.) were added to a solution of the aldehyde from above (0.48 g, 5.00 mmol, 1.00 eq.) dissolved in dichloromethane (50 mL). The reaction mixture was stirred for 2 hours before di-tert-butyl dicarbonate (1.28 mL, 6.00 mmol, 1.20 eq.) and sodium triacetoxy-borohydride (STAB) (2.65 g, 12.50 mmol, 2.50 eq.) were added. After stirring for 18 hours at rt, the reaction was terminated by adding a saturated sodium hydrogen carbonate solution (50 mL). After phase separation, the aqueous phase was extracted with dichloromethane (3 x 50 mL). The combined organic phases were dried over Na2SO4, filtered and the solvent removed under reduced pressure. After purification by column chromatography (petroleum ether:ethyl acetate 10:1), the desired ester (668 mg, 2.25 mmol, 45 %) was obtained as a colorless oil.

$\boldsymbol{R}_{\mathbf{f}}$ = 0.33 (Petroleum ether/ethyl acetate 6:1);

^{1}H-NMR (400 MHz, $CDCl_3$) δ = 3.67 (s, 3H, 11-*H*), 3.19-3.16 (m, 4H, 6-*H*, 7-*H*), 2.31 (t, *J* = 7.39 Hz, 2H, 9-*H*), 2.23-2.19 (m, 2H, 3-*H*), 1.94 (s, 1H, 1-*H*), 1.87-1.80 (m 2H, 8-*H*), 1.66-1.59 (m, 2H, 5-*H*), 1.53-1.48 (m, 2H, 4-*H*), 1.44 (s, 9H, 14-*H*, 15-*H*, 16-*H*) ppm (see figure S1).

^{13}C-NMR (100 MHz, $CDCl_3$) δ = 173.8 (C10), 155.7 (C12), 84.3 (C2), 79.5 (C13), 68.7 (C1), 51.7 (C11), 46.6 (C7), 46.3 (C6), 31.4 (C9), 28.6 (C14, C15, C16), 27.6 (C5), 25.80 (C4), 23.9 (C8) 18.3 (C3) ppm (see figure S2).

ESI-HRMS: *m/z* calculated for $C_{16}H_{27}NO_4$ $[M+Na]^+$: 320.1838, found: 320.1831.

Fragment A synthesis

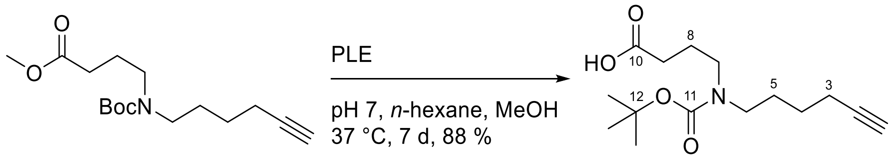

The ester from above (300 mg, 1.01 mmol, 1.00 eq.) was placed in hexane (5.05 mL) and methanol (0.51 mL) before a phosphate buffer solution (pH = 7.4, 50.5 mL) and PLE (48 mg, 720 U) was added. The reaction mixture was heated to 37 °C and cooled to room temperature after seven days. The pH was adjusted to 6 by adding hydrochloric acid (6 M) and the reaction mixture was extracted with ethyl acetate (3 x 60 mL). The combined organic phases were washed with an aqueous sodium hydrogen carbonate solution (5 %, 3 x 200 mL), dried over Na2SO4, filtered and the solvent removed under reduced pressure. Fragment A (252 mg, 0.89 mmol, 88 %) was obtained as a colorless oil.

$\boldsymbol{R_f}$ = 0.64 (Petroleum ether/ethyl acetate 1:1);

^{1}H-NMR (400 MHz, $CDCl_3$) δ = 3.26-3.18 (m, 4H, 6-*H*, 7-*H*), 2.35 (t, *J* = 7.30 Hz, 2H, 9-*H*), 2.23-2.20 (m, 2H, 3-*H*), 1.95 (s, 1H, 1-*H*), 1.86-1.84 (m, 2H, 8-*H*) 1.66-1.61 (m, 2H, 5-*H*) 1.54–1.48 (m, 2H, 4-*H*), 1.45 (s, 9H, 13-*H*, 14-*H*, 15-*H*) ppm (see figure S3).

^{13}C-NMR (100 MHz, $CDCl_3$) δ = 178.7 (C10), 156.0 (C11), 53.7 (C2), 51.9 (C12), 51.4 (C1), 33.9 (C7), 33.7 (C6), 31.4 (C9), 28.8 (C5), 28.7 (C13, C14, C15), 26.4 (C4), 24.4 (C8), 24.0 (C3) ppm (see figure S4).

ESI-HRMS: *m/z* calculated for $C_{15}H_{24}NO_4$ [M-H]: 282.1705, found: 282.1701.

Fragment B synthesis

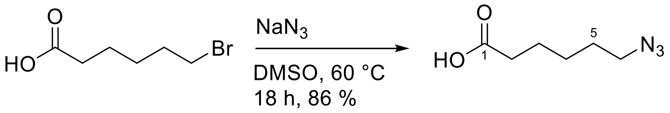

6-Bromohexanoic acid (1.00 g, 5.13 mmol, 1.0 eq.) was dissolved in DMSO (10.3 mL) at room temperature. Sodium azide (1.67 g, 25.63 mmol, 5.0 eq.) was added slowly and the reaction mixture was heated to 60 °C. After 18 hours, the reaction was terminated by adding dist. water (10 mL). After addition of dichloromethane (10 mL), the phases were separated and the aqueous phase was extracted with dichloromethane (3 x 15 mL). The combined organic phases were dried over Na_2SO4, filtered and the solvent removed under reduced pressure. After purification by column chromatography (CH2Cl2:MeOH 20:1), fragment B (0.66 g, 4.20 mmol, 82 %) was obtained as a colorless oil.

$\boldsymbol{R_f}$ = 0.50 (dichloromethane :methanol 6:1);

^{1}H-NMR (400 MHz, $CDCl_3$) δ = 11.37 (bs, 1H, 1-O*H*), 3.27 (t, J = 6.84 Hz, 2H, 2-*H*), 2.36 (t, J = 7.40 Hz, 2H, 6-*H*), 1.64 (m, 4H, 4-*H*, 5-*H*), 1.42 (m, 2H, 3-*H*) ppm (see figure S5).

^{13}C-NMR (100 MHz, $CDCl_3$) δ = 180.2 (C1), 51.3 (C6), 33.9 (C2), 28.6 (C5), 26.2 (C4), 24.2 (C3) ppm (see figure S6).

ESI-HRMS: *m/z* calculated for $C_6H_{10}N_3O_2$ $[M+H]^+$: 156.0773, found: 156.0769.

LNA-Fragment A conjugate synthesis

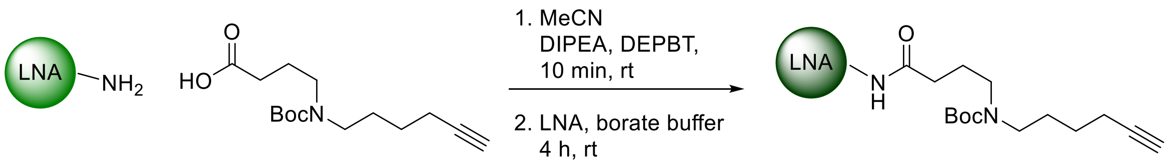

Fragment A (200 mM in acetonitrile, 5 µL), diisopropylethyl amine (DIPEA; 200 mM in acetonitrile, 5 µL) and 3- (diethoxyphosphoryloxy)-1,2,3-benzotriazine-4 (3H)-one (DEPBT; 200 mM in acetonitrile, 5 µL) were mixed in a thermal shaker for 10 minutes at room temperature. Borate buffer (0.5 M, pH = 9.5, 8 µL), LNA-21 (10 nmol in 5 µL H_2O) and dist. H_2O (5 µL) were added and the reaction mixture was left at room temperature for 4 hours without mixing. After that time the reaction was terminated by addition of ethanol (91 µL) and an aqueous sodium chloride solution (5 M, 2 µL) and the solution was incubated overnight at -20 °C. After centrifugation (14.8 rpm, 4 °C, 30 min), the supernatant was decanted, the pellet of the LNA-bearing Fragment A was taken up in ethanol (100 µL) and centrifuged again (14.8 rpm, 4 °C, 15 min). The decantation, take up in ethanol (100 µL) and centrifugation (14.8 rpm, 4 °C, 15 min) was repeated once more before the pellet was dried under vacuum.

SPION-Fragment B conjugate synthesis

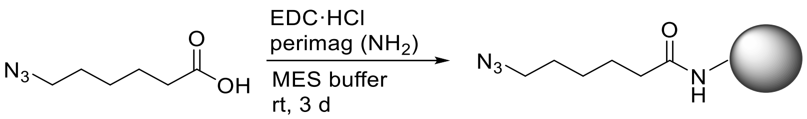

Fragment B (1.2 mg, 7.64 µmol) and (3-dimethylamino-propyl)-ethyl-carbodiimide hydrochloride (EDC-HCl; 1.5 mg, 7.82 µmol) were placed in aqueous MES buffer (0.5 M, 0.3 mL) and mixed in a thermal shaker for 10 minutes at 50 °C. Perimag®-NH_2 (10 mg iron/mL, 1 mL) was added and the reaction solution was mixed in a thermal shaker

for three days at room temperature. The solution was then transferred to a dialysis tube and dialyzed against deionized water for five days. The water was changed three times a day. After completion of dialysis, SPION-Fragment B conjugate (10 mg iron/5.5 mL H_2O) was transferred to Sarstedt tubes and stored at 4 °C.

SPION-LNA -21 conjugate synthesis

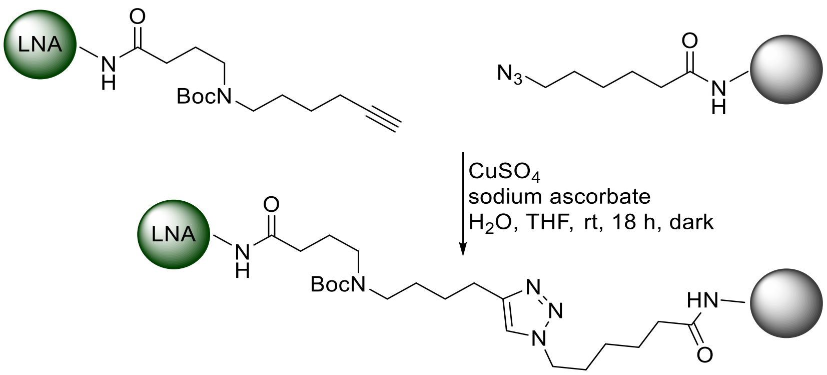

LNA conjugate (25 nmol) was dissolved in H_2O (75 µL) and added to the SPION bearing fragment (10 mg iron/5.5 mL H_2O). THF (2 µL), $CuSO_4$ (cat.) and sodium ascorbate (cat.) were added successively before the reaction mixture was shaken for 18 hours in the absence of light. The solution was then transferred to a dialysis tube and dialyzed against deionized water for four days. The water was exchanged three times a day. After completion of dialysis, SPION-LNA conjugate (10 mg iron/7.5 mL H_2O) was transferred to Sarstedt tubes and stored at 4 °C.

FITC-C6 synthesis

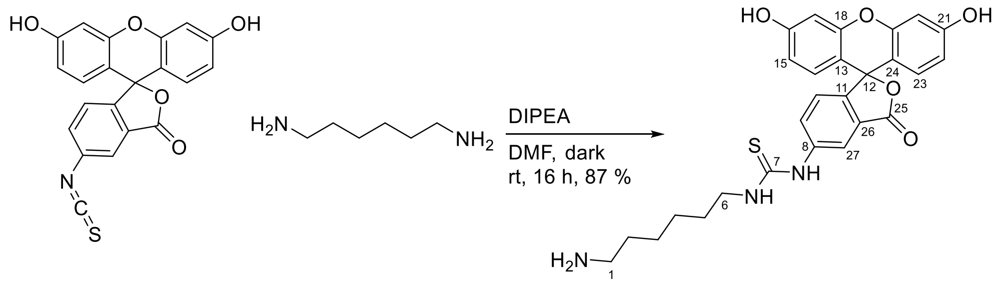

1.6-Diaminohexane (126 µL, 0.96 mmol, 5.00 eq.) was dissolved in dimethylformamide (4.82 mL). N,N-Di*iso*propylethylamine (328 µL, 1.93 mmol, 10.00 eq.) and a solution of fluorescein isothiocyanate (75 mg, 0.19 mmol, 1.00 eq.) in dimethylformamide (1.93 mL) was added slowly. The reaction mixture was stirred for 16 hours at room temperature in the absence of light. The reaction was terminated by removal of the solvent under reduced pressure. FITC-C6 (97 mg, 0.19 mmol, 87 %) was obtained as a reddish oil.

$\boldsymbol{R}_\mathbf{f}$ = 0.20 (Reversed phase chromatography, dist. water:methanol 3:7);

^{1}H-NMR (400 MHz, CD_3OD) *δ* =8.50-8.44 (s, 1H, 27-*H*), 8.09-8.05 (s, 1H, 9-*H*), 7.20-7.16 (d, *J* = 6.71 Hz, 1H, 10-*H*), 6.84-6.78 (m, 1H, N*H*), 6.71-6.66 (d, *J* = 2.63 Hz, 2H, 14-*H*, 23-*H*), 6.60-6.56 (dd, *J* = 9.51, 2.96 Hz, 15-*H*, 17-*H*, 20-*H*, 22-*H*), 3.69-3.61 (bs, 2H, 6-*H*), 2.66-2.65 (s, 2H, 1-*H*), 1.76-1.65 (s, 2H, NH_2), 1.53-1.42 (m, 4H, 2-*H*, 5-*H*), 1.39-1.25 (m, 4H, 3-*H*, 4-*H*) ppm (see figure S7).

^{13}C-NMR (100 MHz, CD_3OD) *δ* = 182.7 (C7), 179.3 (C25), 176.0 (C16, C21),171.7 (C18, C19), 169.4 (C11), 155.3 (C8), 151.6 (C8), 143.1 (C14, C23), 142.0 (C10), 130.4 (C26), 121.1 (C27), 115.5 (C15, C22), 112.6 (C13, C24), 103.6 (C17, C20), 103.4 (C12), 45.8 (C6), 40.7 (C1), 36.5 (C2), 33.1 (C5), 29.7 (C4), 23.7 (C3) ppm (see figure S8).

ESI-HRMS: *m/z* calculated for $C_{27}H_{28}N_3O_5S$ $[M+H]^+$: 506.5968, found: 506.5969.

Test system synthesis (with fluorescein-label)

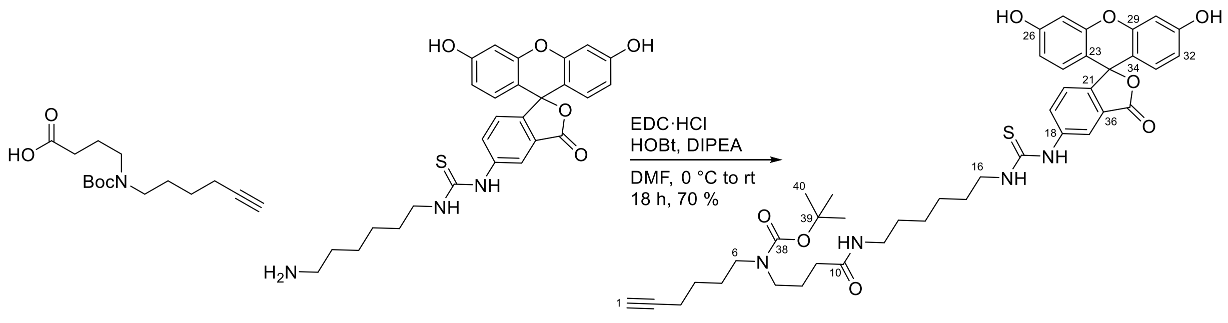

FITC-C6 (30 mg, 0.06 mmol, 1.00 eq.) was dissolved in dimethylformamide (594 µL). Element A (252.3 mg, 0.89 mmol, 15.00 eq.) was placed in dimethylformamide (8.91 mL) and cooled to 0 °C. EDC-HCl (34 mg, 0.18 mmol, 1.50 eq.), HOBt-H_2O (19.3 mg, 0.14 mmol, 1.20 eq.) and *N,N*-diisopropylethylamine (60.6 µL, 0.36 mmol, 3.00 eq.) were added and the solution was stirred for 30 minutes before it was slowly dropped into the FITC-C6 solution. Stirring was continued at room temperature for 18 hours. Then, the solvent was removed under reduced pressure and test conjugate (46 mg, 0.06 mmol, 70 %) was obtained as a reddish oil.

***R*f** = 0.40 (Reversed phase chromatography, dist. water:methanol 3:7);

^{1}H-NMR (400 MHz, $(CD_3)_2SO$) *δ* = 8.50-8.49 (s, 1H,37-*H*), 8.30-8.20 (s, 1H, N*H*), 7.80-7.71 (s, 1H, 19-*H*), 7.18-7.09 (d, *J* = 2.92 Hz, 20-*H*), 6.68-6.65 (m, 1H, N*H*), 6.65-6.60 (m, 2H, 24-*H*, 33-*H*), 6.60-6.54 (m, 2H, 27-*H*, 30-*H*), 6.52-6.45 (m, 2H, 25-*H*, 32-*H*), 3.53-3.42 (m, 2H, 16-*H*), 2.79-2.58 (m, 7H, 1-*H*, 6-*H*, 7-*H*, 11-*H*), 2.42-2.36 (s, 4H, 3-*H*; 9-*H*), 2.04-1.95 (m, 2H, 8-*H*), 1.65-1.40 (m, 6H, 4-*H*, 5-*H*, 15-*H*), 1.38-1.28 (bs, 9H, 40-*H*, 41-*H*, 42-*H*), 1.28-1.20 (bs, 6H, 12-*H*, 13-*H*, 14-*H*) ppm (see figure S9).

^{13}C-NMR (100 MHz, $(CD_3)_2SO$) *δ* = 180.2 (C17), 168.9 (C10), 166.3 (C35), 153.4 (C26, C31, C38), 141.3 (C28, C29), 129.5 (C21), 110.5 (C18), 102.3 (C19), 78.8 (C24, C33), 43.6 (C36, C20), 35.1 (C37), 31.3 (C25, C32), 29.1 (C23, C34), 28.9 (C27, C30), 28.7

(C22), 28.6 (C2), 28.0 (C12, C15), 27.9 (C39, C1), 26.6 (C6, C7), 26.2 (C16), 26.1 (C40, C41, C42), 25.7 (C11), 25.6 (C4, C5, C13, C14), 25.1 (C9), 22.1 (C8), 14.0 (C3) ppm (see figure S10).

ESI-HRMS: *m/z* calculated for $C_{42}H_{49}N_4O_8S$ $[M+H]^+$: 769.3271, found: 769.3271.

Quantification of SPION conjugate

The native perimag® particles carry an unmodified dextran layer(OH). The perimag®-NH2 used in this work were subsequently functionalized by the manufacturer and contained 5-10 nmol amino groups per 1 mg iron/mL.In order to determine the exact degree of functionalization of these commercial batches, an SPDP assay was performed. Perimag®-NH_2 (10 mg iron/mL) was stirred in the presence of a phosphate buffer (pH = 7.4, 3.02 mL) and SPDP (20 mM, 11 mg) at room temperature for 18 hours. The particles were then washed with phosphate buffer (3 x 1 mL) via a MACS® column on a QuadroMACS™ separator. 100 µL of the SPDP-modified solution was removed and DTT (50 mM, 771 µg) was added. Then the solution was stirred for 15 minutes at room temperature, the particles were centrifuged at constant temperature and at 14.8 rpm over a period of 15 minutes. The absorbance at 343 nm was measured from the supernatant. The difference between the absorbance of the supernatants of SPDP-modified particles and the perimag®-NH_2 was used to calculate the degree of functionalization.

The same method was used to quantify the degree of functionalization of SPION conjugate. Here, the sites to which the conjugate has not bound are reacted with SPDP, thus indirectly determining the degree of functionalization.

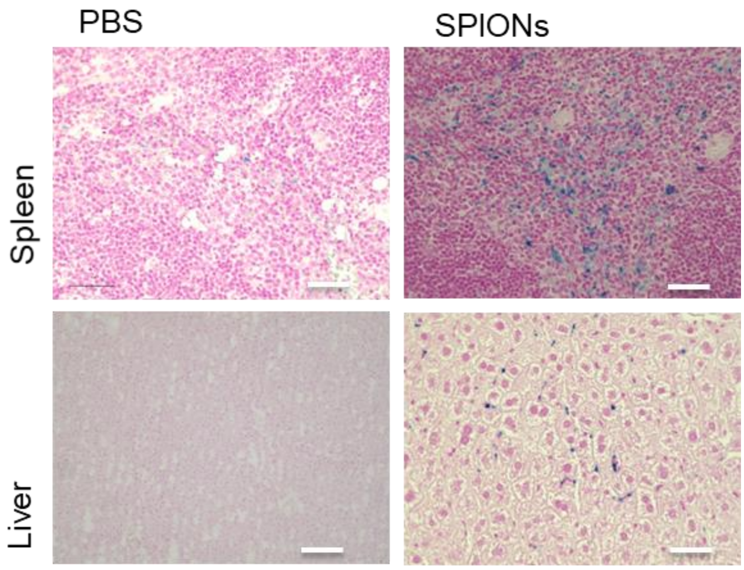

Figure S1: Biodistribution of SPIONs. Mice were intravenously injected with PBS or SPIONS (5 mg iron) and organs were harvested after 7 days. Microscopic images were taken of liver and spleen tissue that were stained with perl´s prussian blue. Representative images of n= 5-7 animals per group. Scale bar=50µm

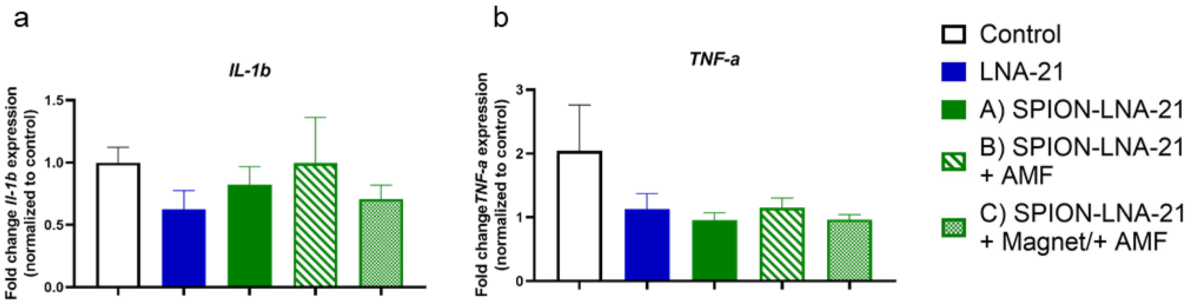

Figure S2: Evaluation of immune response. Mice were either intravenously injected with PBS (N=6), LNA-21 (2.5 mg/kg body weight, N=6) or SPION-LNA-21 conjugate (2.5 mg/kg body weigh, n=5) (group A) or injected with SPION-LNA-21 conjugate (2.5 mg/kg body weight, n=5) and an alternating magnetic field (AMF, 25 mT, 397 Hz) was

applied (group B) or injected with SPION-LNA-21 conjugate (2.5 mg/kg body weight, n=8) and a combination of an external magnetic belt on the heart during the injection and subsequent application of an AMF (group C) was used. Organs were harvested after two days and gene expression of inflammatory markers interleukin-1b (IL-1b) and tissue necrosis factor alpha (TNF-a) were measured in heart tissue. Data are presented as mean ±SEM; One-way ANOVA with Tukey´s multiple comparison test

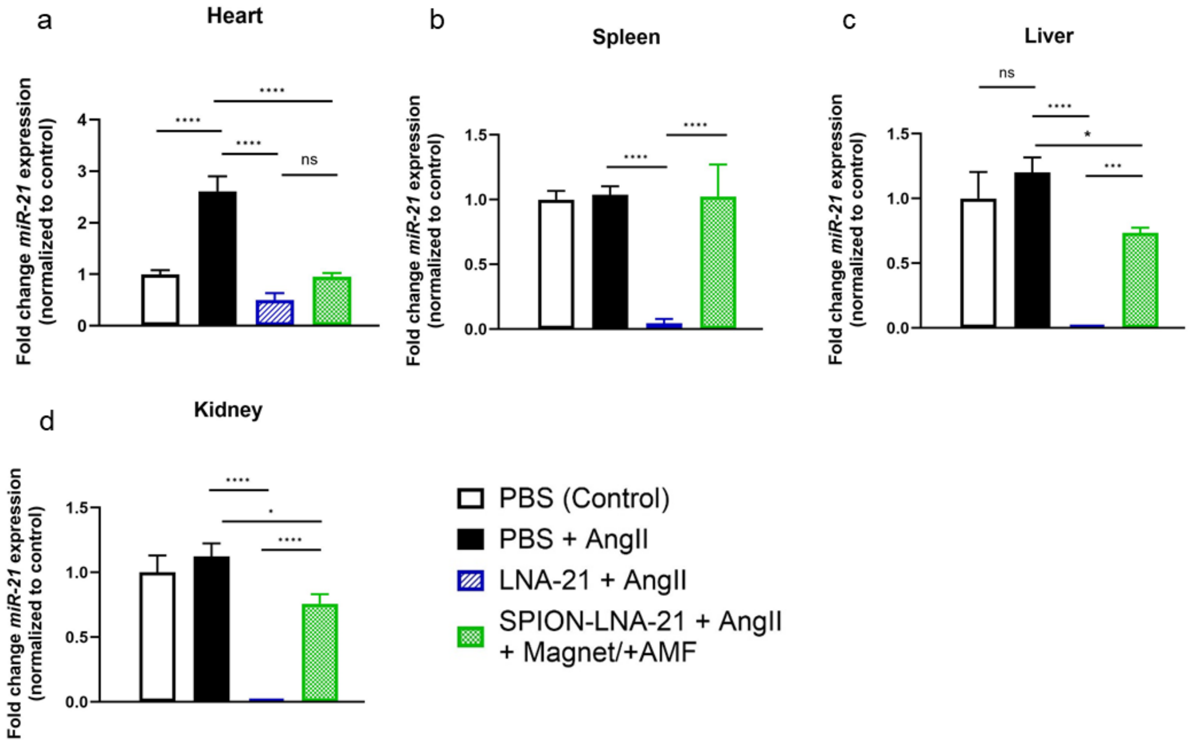

Figure S3: Evaluation of heart-specific delivery of LNA-21 in a therapeutic mouse model. On day 0 an osmotic pump was subcutaneously implanted that constantly releases angiotensin-II (3 mg/kg BW/day) over a time period of 14 days. Control mice were sham-operated. On day three and day ten mice were intravenously injected with PBS, LNA-21 (2.5 mg/kg body weight) or SPION-LNA-21 conjugate (2.5 mg/kg body weight) with a magnetic belt on the heart during the injection and subsequent application of an alternating magnetic field (AMF, 25 mT, 397 Hz) to release the LNA.

Organs were harvested after 14 days and miRNA-21 expression levels were measured in heart (**a**), spleen (**b**), liver (**c**) and kidney (**d**). Data are presented as mean ±SEM; one-way ANOVA with Tukey multiple comparison test; N= 6 in the PBS group, n=7 in the PBS+AngII group, n=9 in the LNA-21+AngII group and n=5 in the SPION-LNA-21 + AngII group

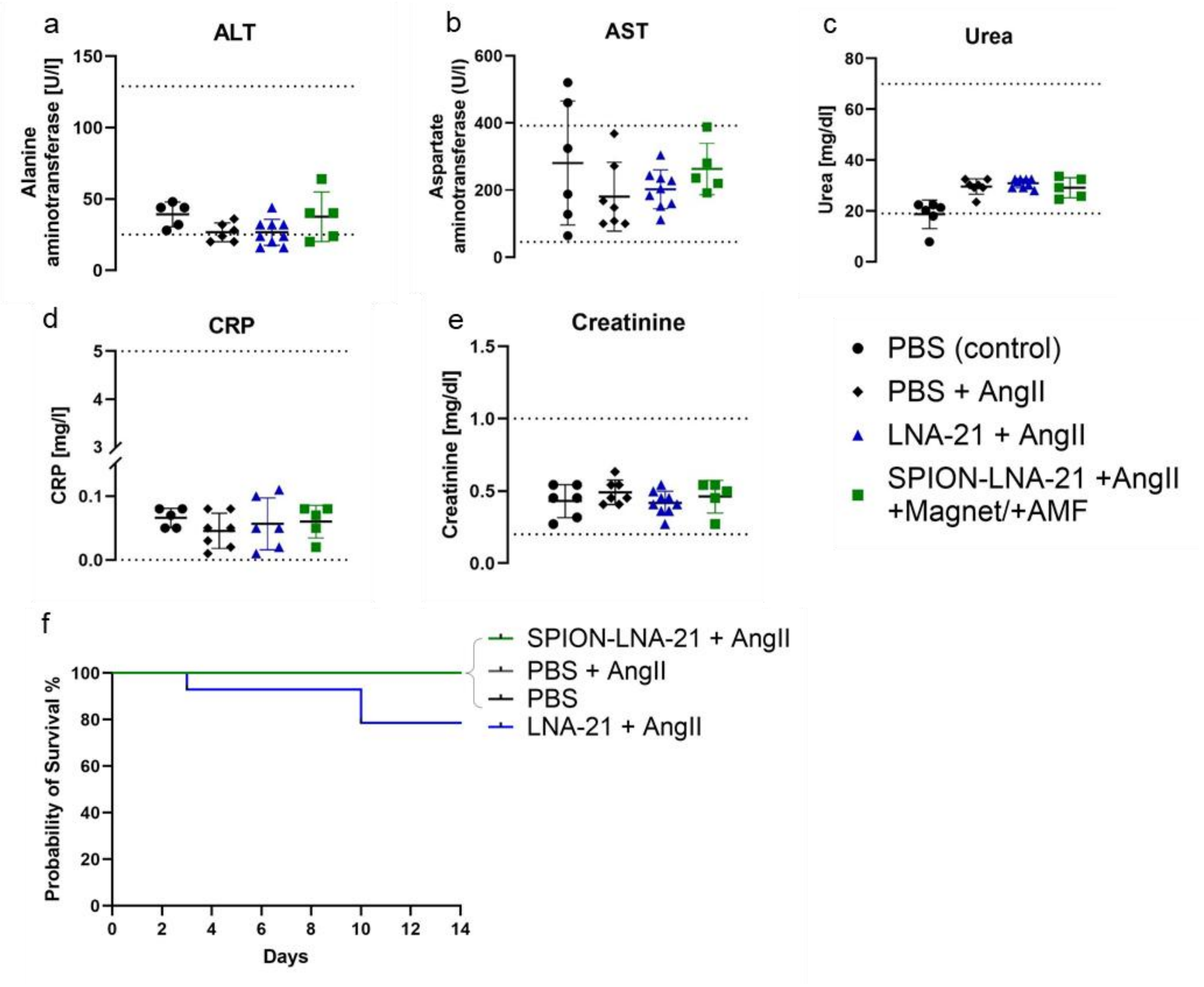

Figure S4: Analysis of plasma markers for renal and liver cytotoxicity. On day 0 an osmotic pump was subcutaneously implanted that constantly releases angiotensin-II (3 mg/kg/day) over a time period of 14 days. Control mice were sham-operated. On day three and day ten mice were intravenously injected with PBS, LNA-21 (2.5 mg/kg body weight) or SPION-LNA-21 conjugate (2.5 mg/kg body weight) with a magnetic belt on the heart during and post injection for 30 min and subsequent application of an

alternating magnetic field (AMF, 25 mT, 397 Hz) for 30 min to release the LNA. Mice were sacrificed after 14 days. Plasma concentrations of liver (ALT= Alanine aminotransferase (**a**), AST = Aspartate aminotransferase (**b**),) and kidney damage (creatinine (**c**) and urea (**d**)) as well as C-reactive protein (CRP; (**e**)) as a marker for inflammation were measured via ELISA. Data are presented as mean ±SEM; N= 5-9 animals per group. (**f**) The probability of survival was analyzed vie Kaplan-Meier-curve. N= 5-9 animals per group

NMR Spectra:

Ester

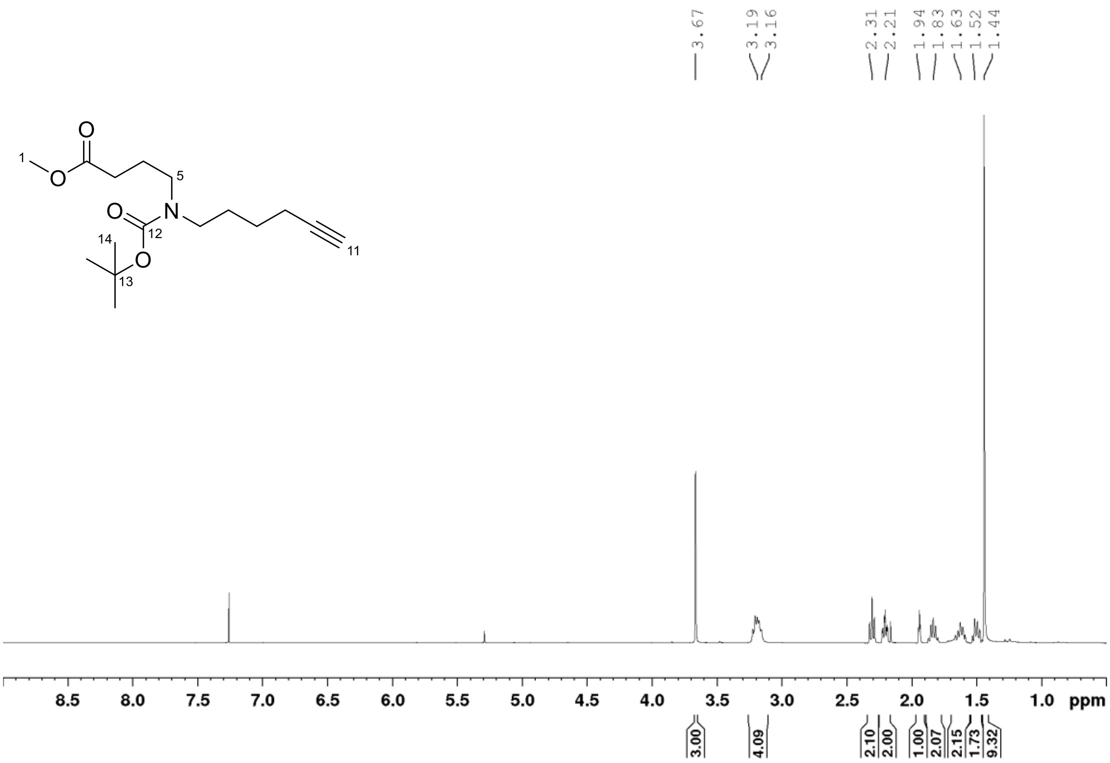

Figure S5: ^{1}H-NMR spectrum (400 MHz) of ester in $CDCl_3$.

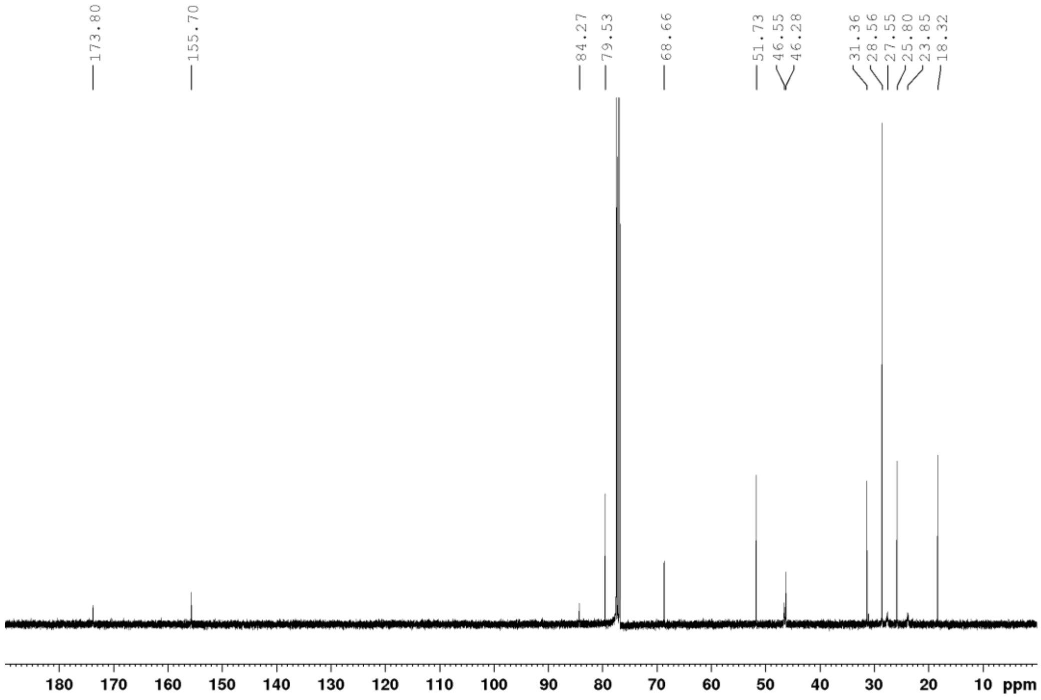

Figure S6: ^{13}C-NMR spectrum (100 MHz) of ester in $CDCl_3$.

Fragment A

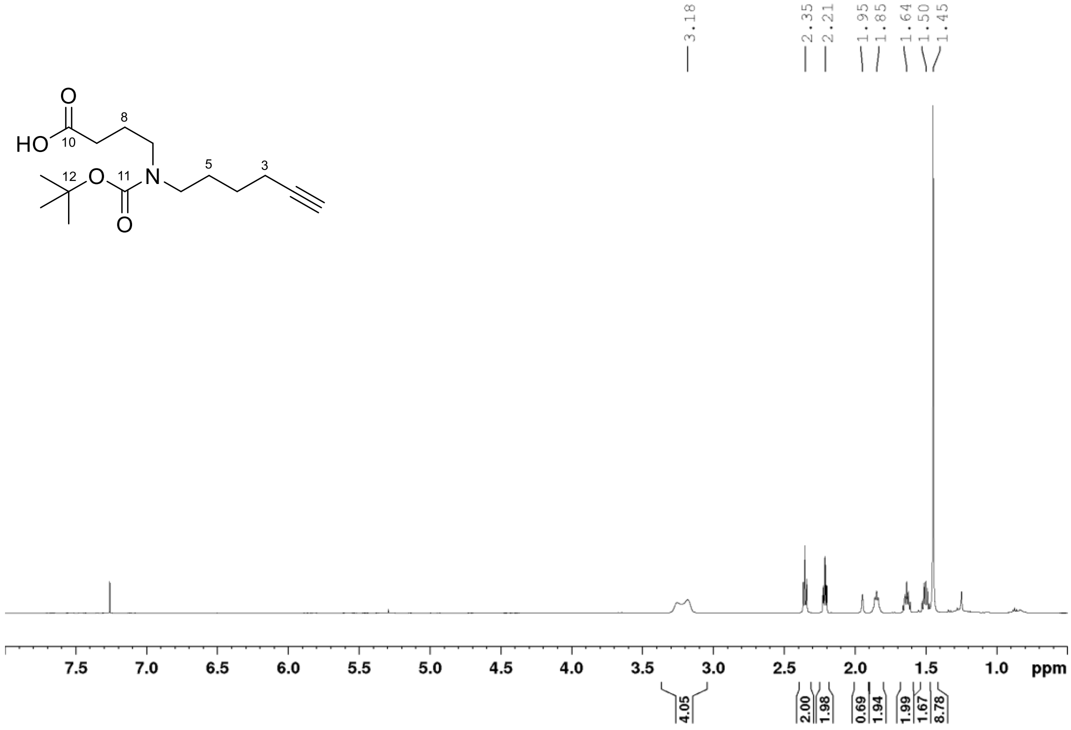

Figure S7: ^{1}H-NMR spectrum (400 MHz) of fragment A in $CDCl_3$.

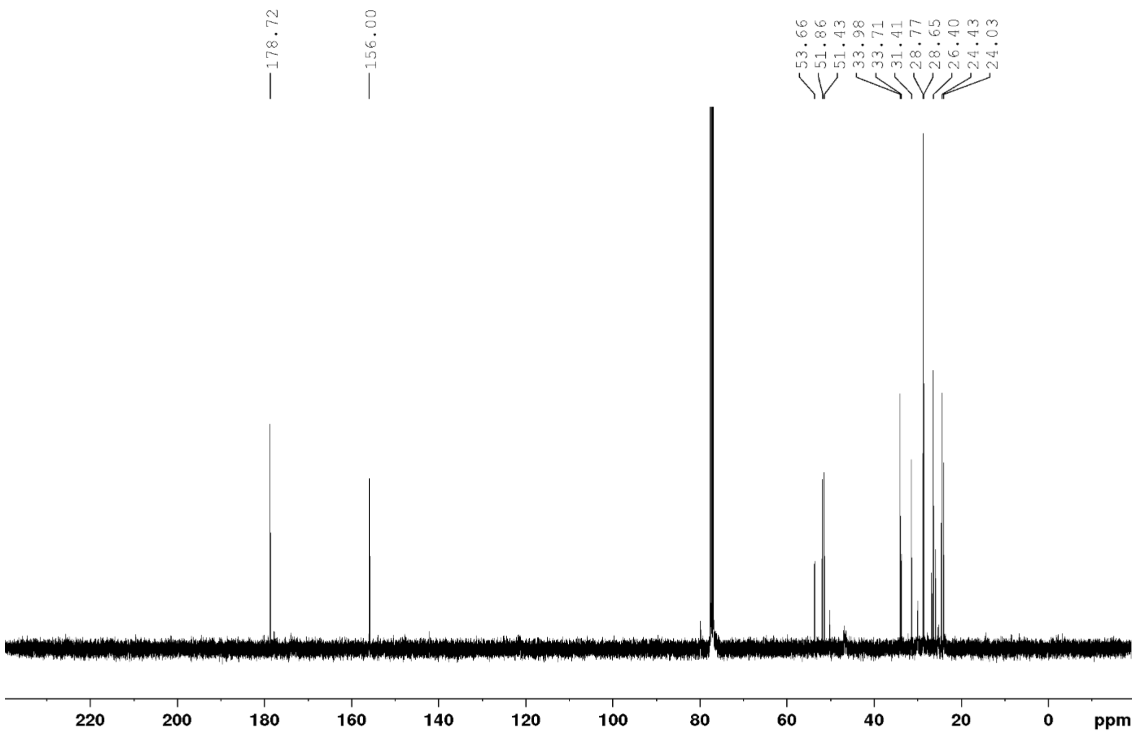

Figure S8: ^{13}C-NMR spectrum (100 MHz) of fragment A in $CDCl_3$.

Fragment B

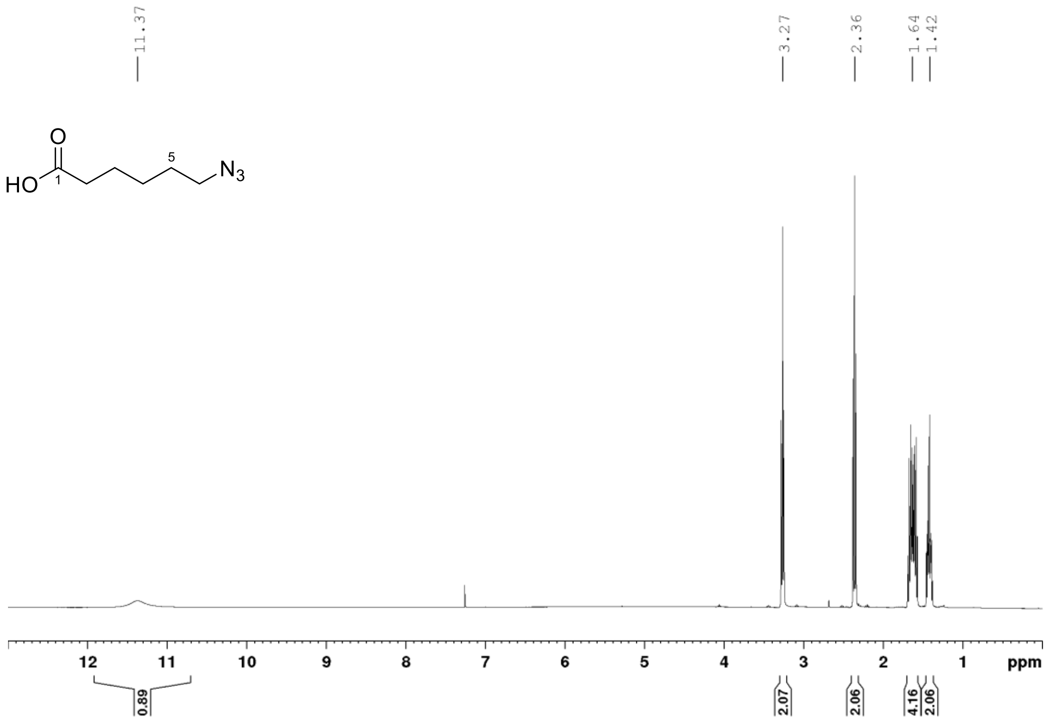

Figure S9: ^{1}H-NMR spectrum (400 MHz) of fragment B in $CDCl_3$.

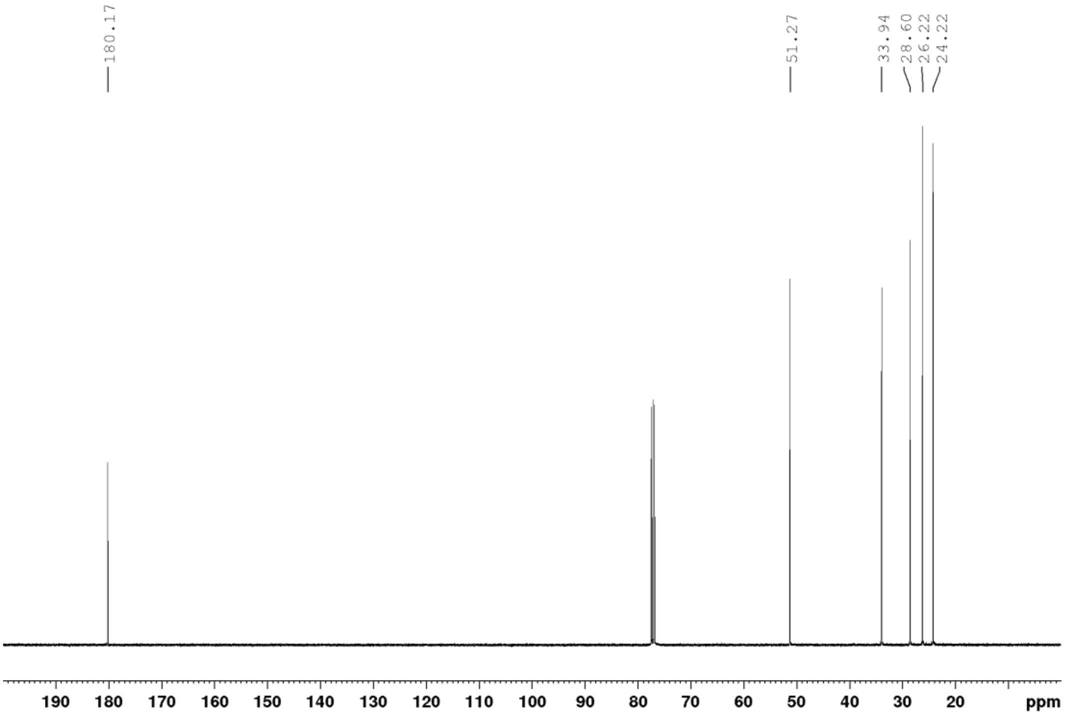

Figure S10: ^{1}H-NMR spectrum (100 MHz) of fragment B in $CDCl_3$.

FITC-C6

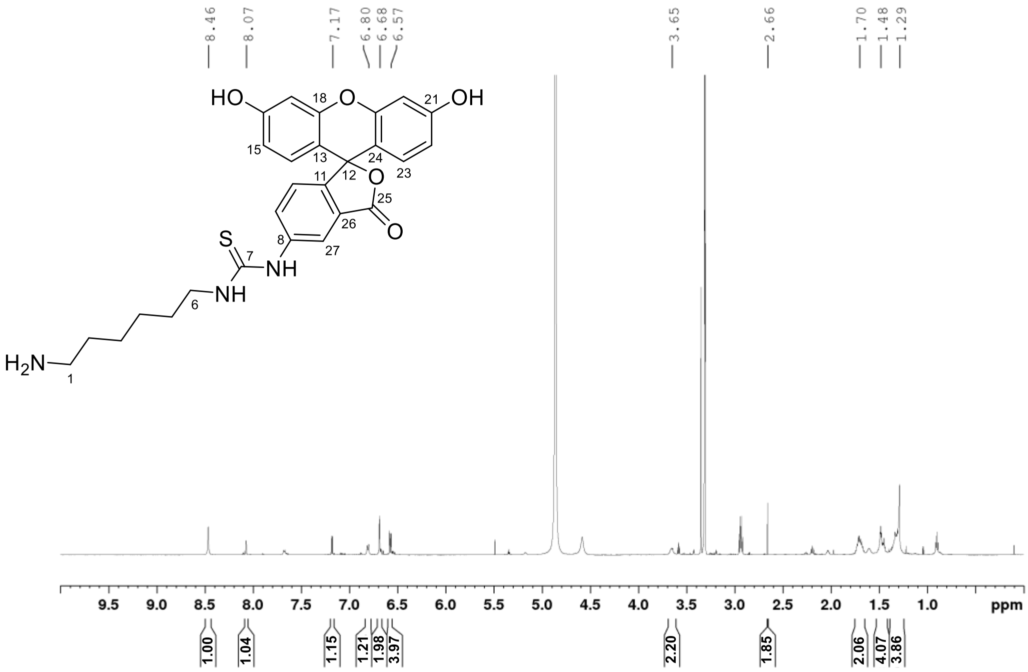

Figure S11: ^{1}H-NMR spectrum (400 MHz) of FITC-C6 in D_3COD.

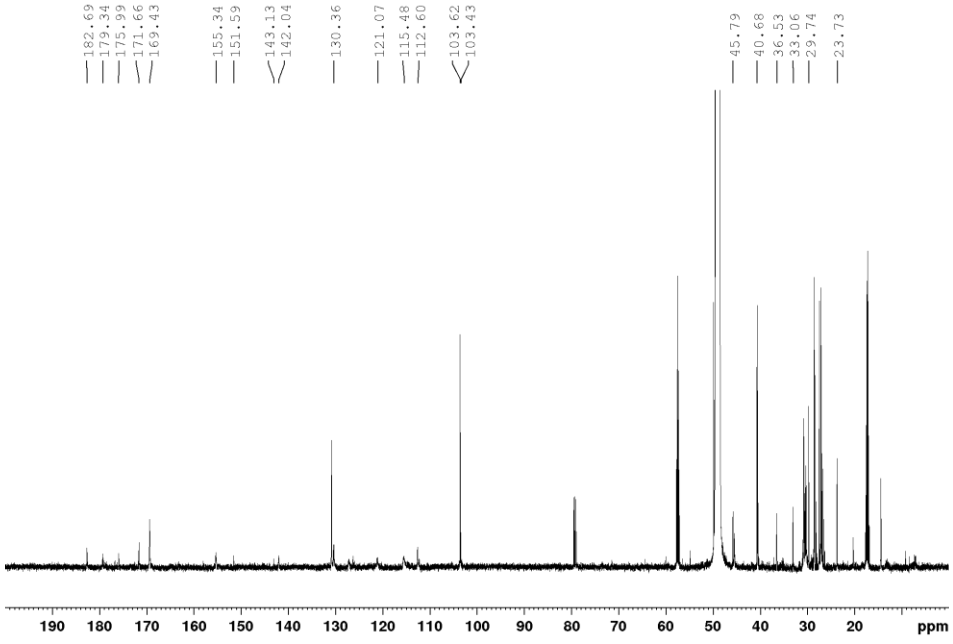

Figure S12: ^{13}C-NMR spectrum (100 MHz) of FITC-C6 in D_3COD.

Test substrate

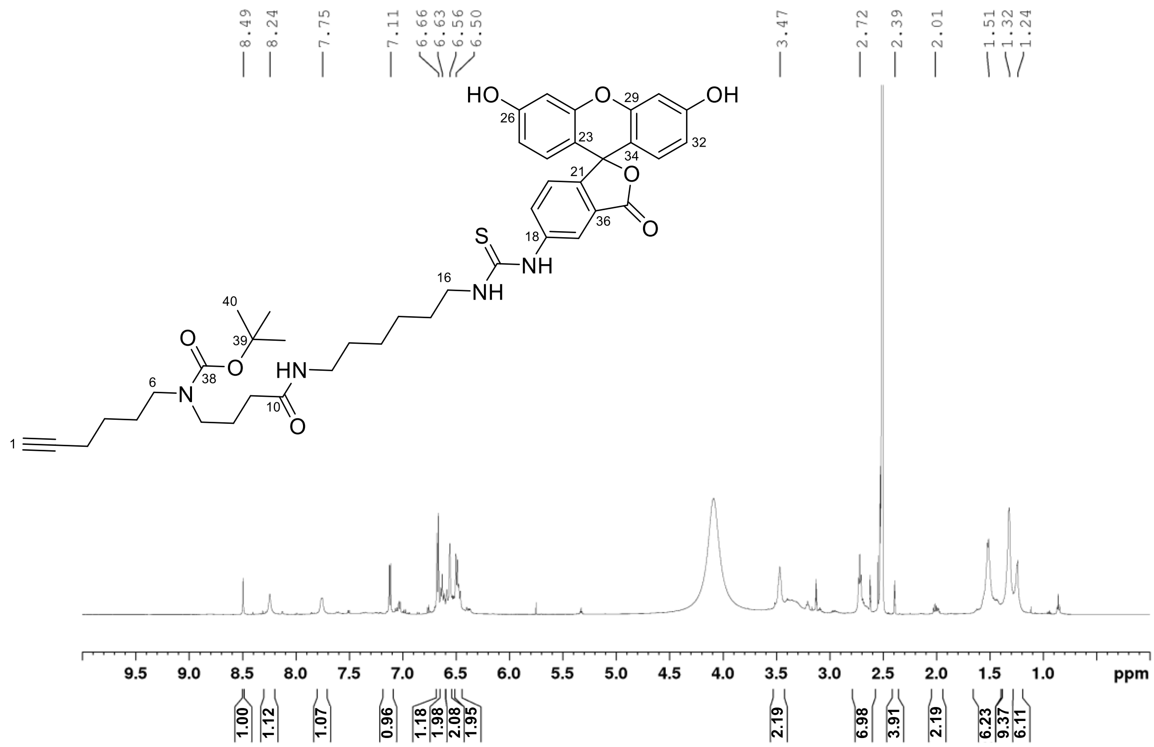

Figure S13: ^{1}H-NMR spectrum (400 MHz) of test substrate $(D_3C)_2SO$.

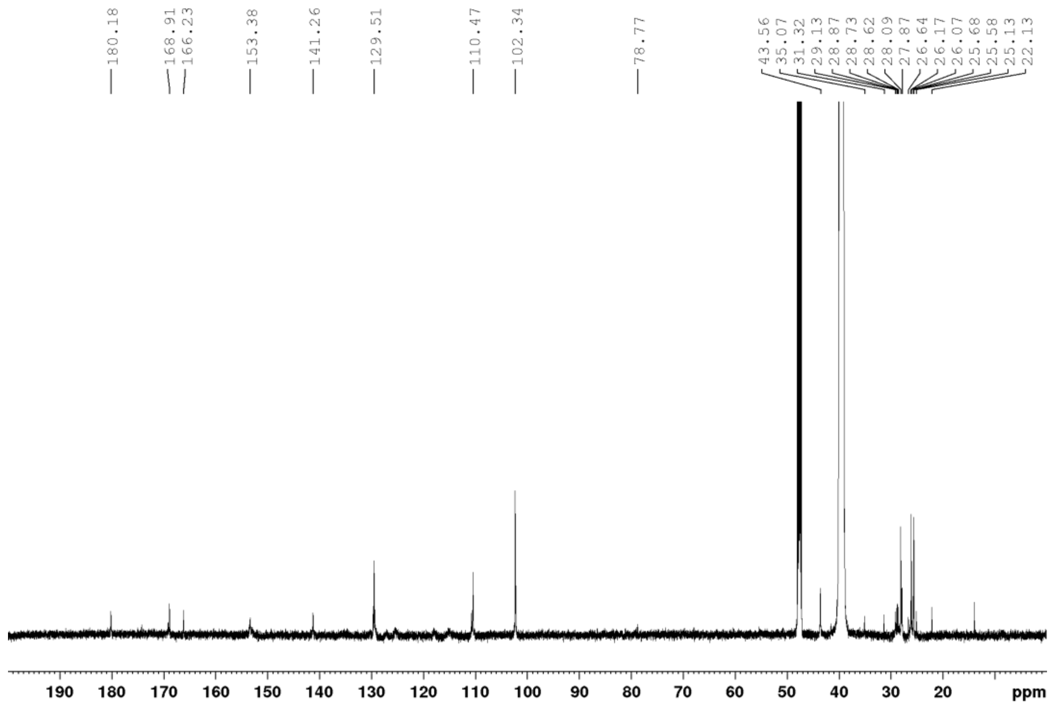

Figure S14: ^{13}C-NMR spectrum (100 MHz) of test substrate in $(D_3C)_2SO$.
